# Transforming Community‐Based Rehabilitation Services: A National Redesign Using Experience‐Based Co‐Design

**DOI:** 10.1111/hex.70330

**Published:** 2025-06-23

**Authors:** Shamala Thilarajah, Karina Dancza, Zhen Zhen Chen, Clara X. Q. Wong, Clement C. Yan, Susan Niam, Yee Sien Ng, Elizabeth Lynch, Leonid Churilov, Wei Kang Tan, Emelin Tan, Li Khim Kwah

**Affiliations:** ^1^ Department of Physiotherapy Singapore General Hospital Singapore Singapore; ^2^ Chief Allied Health Officer's Office, Ministry of Health Singapore Singapore; ^3^ Health and Social Sciences Cluster Singapore Institute of Technology Singapore Singapore; ^4^ Department of Occupational Therapy National University Hospital Singapore Singapore; ^5^ Department of Physiotherapy Tan Tock Seng Hospital Singapore Singapore; ^6^ Department of Physiotherapy Sengkang General Hospital Singapore Singapore; ^7^ Department of Rehabilitation Medicine Singapore General Hospital Singapore Singapore; ^8^ Caring Futures Institute Flinders University Adelaide Australia; ^9^ University of Melbourne Melbourne Australia; ^10^ Health Analytics Division Ministry of Health Singapore Singapore; ^11^ Department of Respiratory Therapy Tan Tock Seng Hospital Singapore Singapore

**Keywords:** community participation, community‐based rehabilitation, experience‐based co‐design, health services research, patient‐centred care

## Abstract

**Background:**

To understand the experiences of clients, caregivers and staff in community day rehabilitation centres (DRCs) and identify areas for improvement in service processes and the clinical care of stroke, frailty and hip fracture, a national quality improvement project was undertaken using experience‐based co‐design (EBCD). The goal was to prioritise and co‐design system‐level changes for community‐based rehabilitation care in Singapore.

**Methods:**

The EBCD methodology comprised of eight stages: (1) site observations/time‐motion studies, (2) interviews, (3) development of trigger film, (4) staff feedback events, (5) client/caregiver feedback events, (6) joint workshop, (7) small co‐design groups and (8) celebration event. In addition, we collected surveys and case note reviews to capture the perceived and actual delivery of guideline‐based care for stroke, frailty and hip fracture.

**Results:**

Over a period of 2.6 years, we engaged over 80 clients and caregivers and 250 staff from 20 DRCs in the EBCD process. Triangulation of data from the site observations, interviews, surveys and case note reviews identified four themes: (1) Best practice care, (2) Person‐centred care, (3) Allied health professional needs and (4) Service design. Person‐centred care was desired by clients, caregivers and staff but was sometimes hindered by factors like tight scheduling and high turnover. Care was partially aligned with international guidelines, though some strongly recommended interventions were inconsistently delivered. Staff interviews and site observations revealed potential for more direct client care, teaching, research and quality improvement, with fewer administrative duties. New care models were sought, including social connections beyond DRCs, clearer maintenance rehabilitation criteria and financial incentives for transitioning to maintenance rehabilitation. A 12‐min trigger film based on client/caregiver interviews was used during the feedback events and workshops. Three co‐design workgroups were formed to develop clinical practice guidelines for stroke rehabilitation, a workplace learning framework for allied health, and community rehabilitation recommendations.

**Conclusion:**

EBCD was successfully used to identify gaps and co‐design system‐level solutions to improve community‐based rehabilitation care in Singapore. Further solutions at the organisational and individual levels are needed.

**Patient or Public Contribution:**

Our study used EBCD to actively involve clients, caregivers and healthcare staff. Clients with lived experience of stroke, frailty and hip fracture identified key priorities and contributed to qualitative data interpretation during the feedback event. Notably, a trigger film incorporating clients' and caregivers' experiences of community rehabilitation services was developed and verified for accuracy during the feedback event, before being played at the joint workshop for all. During the joint workshop, priority statements for discussion were collaboratively identified, and solutions were co‐developed. Clients and caregivers subsequently contributed to the development of the clinical practice guidelines for stroke rehabilitation. Throughout the project, the insights from clients and caregivers helped to ensure that our study's findings were relevant and actionable. We acknowledge the invaluable role of our patient and public partners in shaping this study and driving meaningful healthcare improvements.

## 1 Introduction

In the past decade, there has been increasing recognition of the need to transform community‐based rehabilitation services to address the growing demand for such care [1]. Despite WHO's call to action, many countries struggled to prioritise rehabilitation within their health systems, facing challenges such as fragmented governance, inadequate funding and workforce shortages [2]. Even in high‐income countries, where one in three individuals may require rehabilitation services in their lifetime, access remained uneven [3]. In Singapore, as part of the Ministry of Health (MoH)'s Healthcare 2020 Masterplan, a series of reforms were set out to expand the capacity of intermediate and long‐term care (ILTC) rehabilitation services in anticipation of the needs of our rapidly ageing population. These reforms included increasing beds and building more ILTC facilities; increasing intakes of medical, nursing and allied health degree programmes; introducing career progression and compensation packages for healthcare staff; and raising government subsidy rates for long‐term care services [4]. A care model termed as the National One‐Rehab framework (‘One‐Rehab’) had also been introduced to ensure people received access to the right rehabilitation care at the right time and right place [5].

The One‐Rehab framework provided guidance on the rehabilitation care needs and care pathways for six conditions: stroke, deconditioning, hip fracture, musculoskeletal conditions, amputations and spinal cord injury. Allied health professionals (AHPs) used standardised outcome measures to compare client outcomes across sites and national benchmarks formed, similar to other countries [6]. This data can reduce unwarranted variations in healthcare outcomes, allowing for sites to evaluate the quality of care they provide, and exchange and share good practices [7]. To ensure clients received effective rehabilitation from acute to the community, the Community Rehabilitation Transformation Workgroup (CRTW) was formed. The workgroup's vision was to co‐design community rehabilitation reforms with the people who accessed and delivered rehabilitation services. Co‐design is an emerging methodology for policy formulation and health systems design, particularly for large‐scale national reforms [8]. This study employed experience‐based co‐design (EBCD) [9, 10], a participatory action research method, to shape health system design and policies aimed at enhancing clinical care in the ILTC sector.

Therefore, our study aimed to answer the following questions:

a. What are the experiences of clients, caregivers and staff in Singapore's centre‐based day rehabilitation services?
b. What is the current state of clinical care and organisational practices in centre‐based day rehabilitation services?
c. What areas do clients, caregivers and staff prioritise that can improve their experiences of centre‐based day rehabilitation services?

## 2 Methods

### 2.1 Study Design and Setting

Using the EBCD methodology, we conducted a national quality improvement project with 20 different day rehabilitation centres (DRCs), which formed 66% of the market share in the ILTC sector. The CRTW was formed under the National One Rehabilitation Steering Committee (NORSC) established by the MOH, Singapore, to re‐examine rehabilitation models in response to the rapidly ageing population and increasing demand for community rehabilitation services. The CRTW was led by 2 senior advisers, chaired by 2 co‐leads and included 15 members from academia, clinical research, community service and senior DRC staff/champions, supported by a secretariat team from the MOH Chief Allied Health Officer's Office (CAHOO). Before the start of the project, the CRTW also consulted and engaged with rehabilitation leaders in the hospitals and ILTC sector, including directors of allied health, head of departments and managers and 23 medical subject matter experts for feedback on the project plan, and nomination of senior DRC staff/champions who can effect change in the workplace.

**Figure 1:**
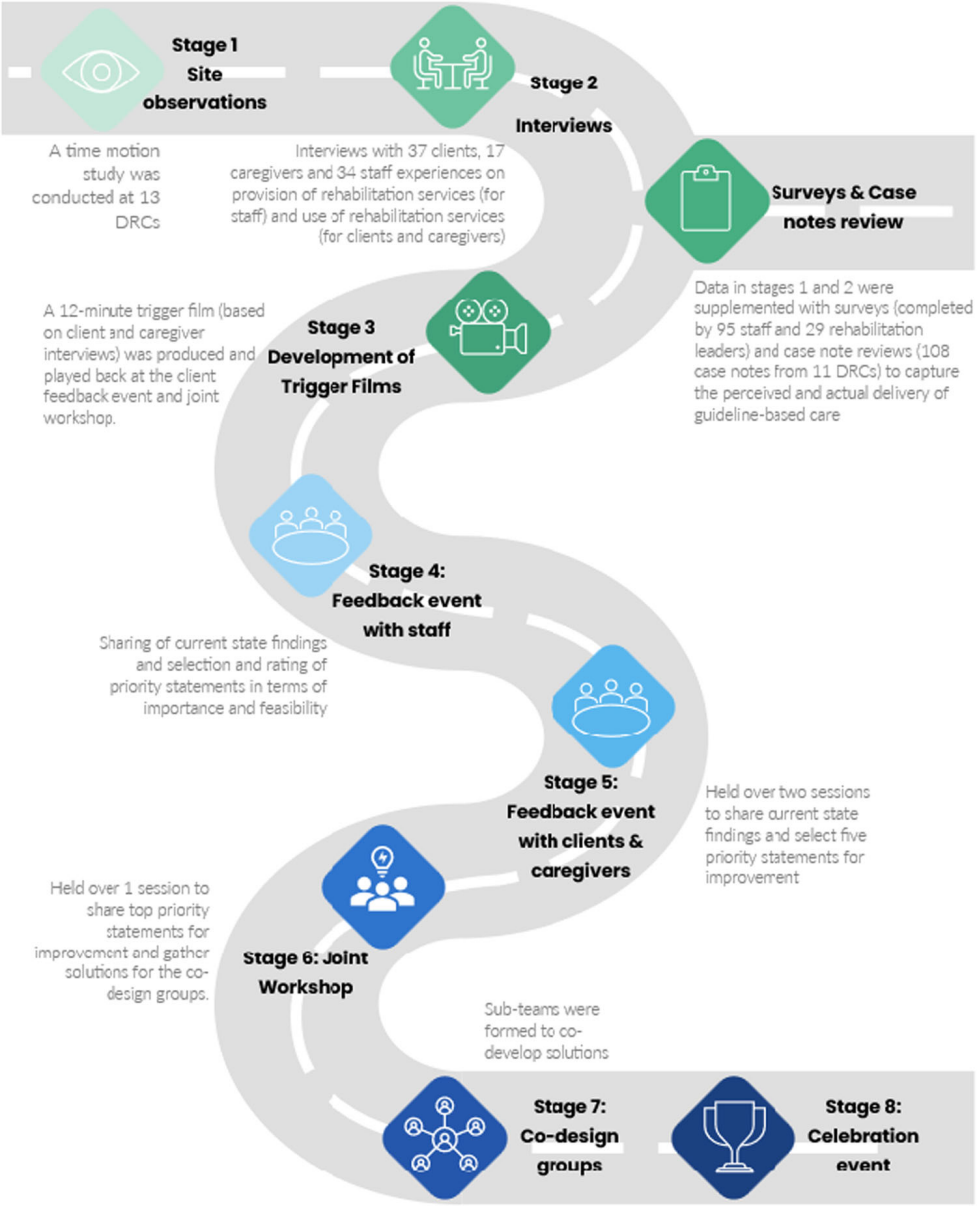
Process of EBCD methodology supplemented with surveys and case note reviews.

EBCD is a form of participatory action research approach that integrates ethnographic research and health service design methods with consumer engagement [9, 10]. Used in both research and quality improvement studies in the United Kingdom, Australia, New Zealand, Sweden, South Africa and Canada [10], it facilitates service improvements by bringing service users and providers together to share experiences, identify and prioritise issues, and co‐design solutions to improve service delivery [9]. There are eight stages in the EBCD process: (1) site observations/time‐motion studies; (2) interviews with clients, caregivers and staff; (3) development of trigger film; (4) staff feedback event; (5) client and caregiver feedback event; 6) joint workshop with clients, caregivers and staff; (7) small co‐design groups; and (8) celebration event [10]. To ensure a comprehensive overview of clinical care and service processes, we supplemented EBCD with surveys and case note reviews (Figure 1). We referred to official documents, published studies and high‐quality clinical practice guidelines to guide our methods for EBCD and templates for site observations/time‐motion studies, interviews, surveys and case note reviews, feedback events and joint workshop (Supporting Information S1).

### 2.2 Funding and Background of Facilitators

The Singapore MoH provided the staffing that helped to support the different stages of the EBCD process. Clients and caregivers were also reimbursed for their time and effort and received a SGD$20 voucher for their participation in the feedback events and joint workshop. The study methods were designed by K.L.K. and S.T., both PTs with PhDs and 20 years of work experience. K.L.K., S.T. and K.D. (an occupational therapist [OT] with a PhD and 23 years of work experience) led the facilitation for the feedback events, joint workshop and co‐design groups. All three facilitators have extensive workshop facilitation experience, with K.L.K. and S.T. having led co‐design workshops with stroke survivors, caregivers and healthcare professionals [11] and K.D. having led co‐design workshops with occupational therapy students and staff [12, 13].

### 2.3 Site Observations/Time‐Motion Studies

Participation in the site observations/time‐motion studies was optional. For DRCs that were willing to participate, junior and senior therapists were observed at the DRC for the day by non‐therapists. A secretariat team member was randomly allocated to observe one therapist for 15‐min blocks across 9–10 h from 8 AM to 6 PM, depending on their working hours. Using a pre‐determined classification of activities which included six categories (clinical, clinical‐related administration, operations‐related administration, teaching/supervisory, research and quality, and others), the secretariat team member would observe and record the activity in which the therapist is engaged in for each 15‐min block (Supporting Information S1 and S2). To provide further context, DRCs vary in terms of the caseload and manpower allocation (Supporting Information S8). Typically, most centres opened for 9–10 h on weekdays and some opened for 4 h on weekends in Singapore. Site observations for this project were only conducted on weekdays.

### 2.4 Interviews

Interview methods were reported in accordance with the Consolidated Criteria for Reporting Qualitative Research (COREQ) checklist [14]. CRTW members and the secretariat team (*n* = 19; 15 females and 4 males) conducted the semi‐structured interviews with clients, caregivers and staff. A 3‐h training session led by K.D., S.T. and K.L.K. was held with interviewers prior to and included training on the principles of reflexivity and practice of conducting the interviews. CRTW members identified eligible participants for the interviews. These included any AHP who had provided centre‐based day rehabilitation services for clients with stroke, deconditioning or hip fracture in the past 6 months, clients and caregivers who had used centre‐based day rehabilitation services in the past 6 months, and clients and caregivers who were referred but did not attend centre‐based day rehabilitation services in the past 6 months. Interviews took approximately 30–60 min to conduct and were done face‐to‐face or via Zoom at the centres or participants' preferred locations. Audio and/or video recordings were collected during the interviews as per consent from participants and transcribed verbatim. Field notes were made by interviewers on a standardised template. The number of interviews was guided by information power, with data reviewed iteratively to assess richness and relevance [15]. To minimise bias, we ensured interviewers had no prior relationship with the clients, caregivers and/or staff being interviewed. Interview questions centred on the experiences of delivering and/or accessing day rehabilitation services (e.g., positive experiences, areas that can be improved and what best practice care meant to them) (Supporting Information S3).

### 2.5 Surveys and Case Note Reviews

Two web‐based/online surveys were conducted—the clinical survey and the organisational survey. Surveys were conducted from January 2022 to March 2022. The surveys were developed on Qualtrics and disseminated through the CRTW network. Participants accessed the surveys via email links and QR codes. Participants for the clinical survey were physiotherapists (PTs), OTs or Speech and Language Therapists (SLTs) who had provided clinical care to at least one client with stroke, deconditioning or hip fracture in the past 6 months. Participants for the organisational survey were rehabilitation leaders and/or nominated champions within the DRC. The clinical survey questions focused on the frequency of recommended assessments and treatments used by PTs, OTs and SLTs as part of their clinical care for clients with stroke, deconditioning and hip fracture. The organisational survey questions focused on the current service processes in place (e.g., availability of resources/services and protocols, capacity of physicians and AHPs working at DRC, practice of goal setting and reviewing of clients on different rehabilitation models [‘Active Rehabilitation’ and ‘Maintenance Rehabilitation’], practice of skills sharing and availability of processes for skills sharing) (Supporting Information S1 and S4).

Participation in the case note reviews was voluntary. Each participating site was asked to provide approximately four case notes on patients with strokes, three case notes on patients with deconditioning and three case notes on patients with hip fracture case notes completed within the last year. All reviews were conducted on‐site at the DRCs. While the clinical survey captured perceived delivery of recommended care, the case note reviews captured actual delivery of recommended care. Using a standardised protocol to extract and code data, four members of the CAHOO team carried out the case note reviews (Supporting Information S1 and S5).

### 2.6 Feedback Events, Joint Workshop and Co‐Design Groups

**Figure 2:**
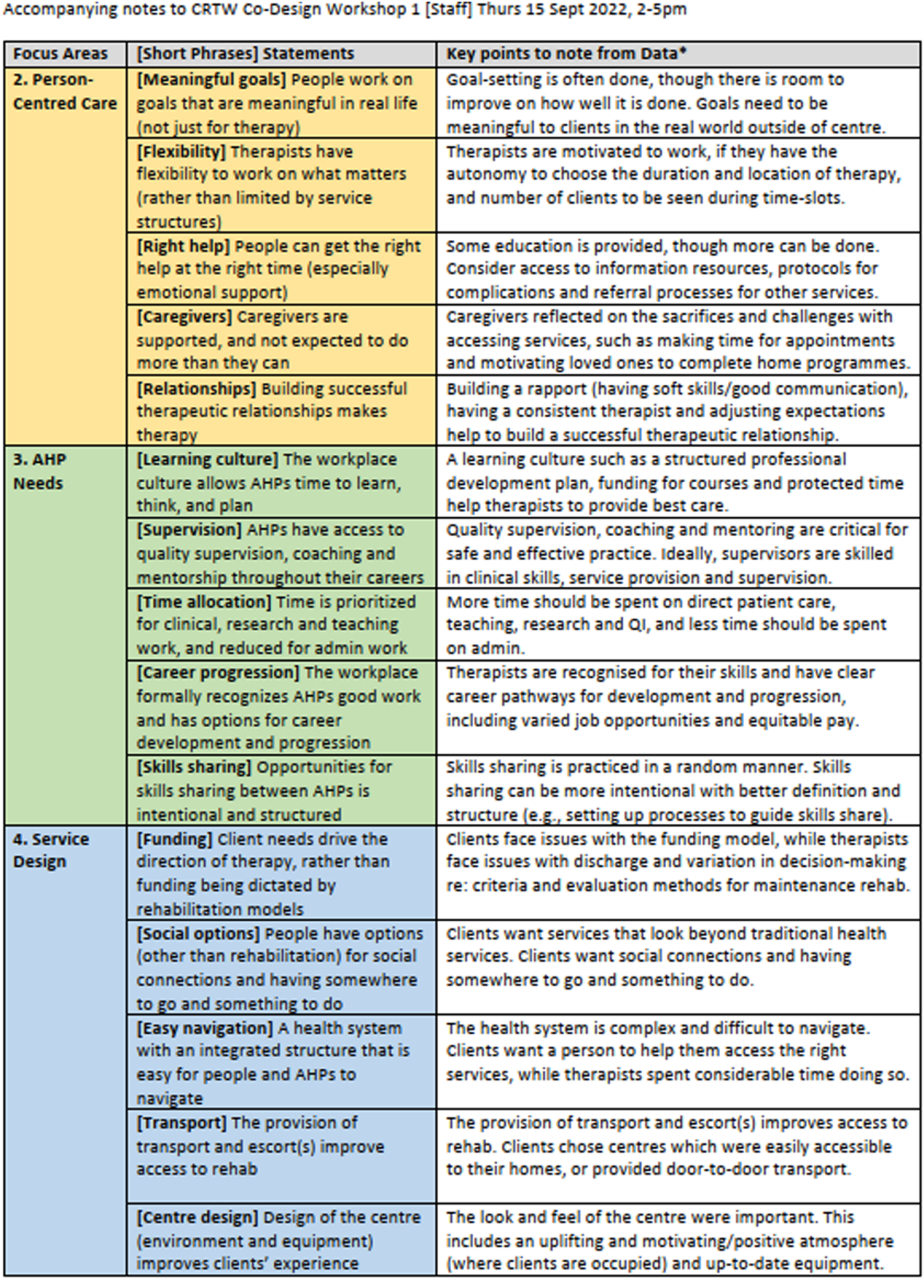
Priority statements used during the feedback events and joint workshop. AHP, Allied Health Professional*Data includes data captured from surveys, interviews, case note reviews and time motion studies

A series of events was conducted to gather feedback and prioritise improvements for community rehabilitation services. These included a staff feedback event in September 2022, two client and caregiver feedback events in October 2022, and a joint workshop in April 2023, each lasting 3 h and held face‐to‐face. The events were designed to be accessible and inclusive, with locations chosen due to their suitability for wheelchair users. If clients had cognitive and/or language deficits but wanted to participate, they were accompanied by their caregivers/family members. When clients were sharing, they were also given the space and time to communicate in their groups or with the entire room. This was ensured by the lead facilitators, who also conducted training for the CRTW team members in charge of facilitating small group discussions at the respective tables during the feedback event and the joint workshop. All facilitators were provided with accompanying materials to help guide the small group discussions and assist clients and caregivers with their queries (Supporting Information S6). Materials for the event and workshop involving clients and caregivers were also produced in print (with enlarged font) for ease of access. During these events, participants were presented with data from the prior stages of the EBCD, summarised into four themes: (1) Best practice care, (2) Person‐centred care, (3) AHP needs and (4) service design and 15 priority statements (Figure 2). The 15 priority statements stemmed from the 17 sub‐themes of the interviews (Supporting Information S7) and corroborated with data from the surveys and care note reviews. As best practice care (comprising two sub‐themes) was deemed a non‐negotiable priority for improvement, it was not included as a priority statement for rating during the feedback events. Before the staff feedback event, all staff were also sent a 40‐min video summarising the EBCD results from the interviews, surveys and case note reviews. This allowed staff time to review the findings, reflect on their practices and prepare questions to ask. During the staff feedback event, staff members engaged in three rating exercises: first, to select their top five priority statements; second, rate each of the 15 statements in terms of importance on a scale of 1–10; third, identify the most important 10 priority statements and rate them in terms of importance and feasibility using a graph theory‐based voting system, similar to the process used in an Australian case study that prioritised stroke guideline recommendations for implementations [16]. Statements that scored high in terms of importance and feasibility indicated that staff deemed them as most important in delivering the best care to clients and caregivers and most feasible to solve based on current resources (i.e., without additional funding or staffing within 12–18 months). Of the 15 priority statements, clients and caregivers were asked to select their top five priority statements for improving their rehabilitation experiences. A 12‐min trigger film was used to stimulate discussion and reflection on community rehabilitation experiences. The trigger film was played at the feedback event with clients and caregivers and at the joint workshop (Figure 1). The trigger film showed clients and caregivers sharing their experiences of day rehabilitation services (based on their responses to the interview questions). These were also shown in line with the themes of best practice care, person‐centred care and service design previously identified. The secretariat team edited the film based on the guidance of existing EBCD toolkits from the Australian Healthcare and Hospitals Association and the King's Fund, the Point of Care Foundation (Supporting Information S1). Consent was obtained for any clients or caregivers featured in the film, and audio/video recordings were used as per their consent. A medical social worker was present to provide emotional support. During the joint workshop, results from the rating exercises were presented. Participants then went into one of three breakout groups where discussions were facilitated by the CRTW team members. Discussions focused on the priority statements that were found to be important to staff, clients and caregivers, had the most spillover (i.e., if solved, would impact two or more priority statements) and were under the remit of the CRTW. The breakout group sessions focused on understanding the current state regarding the priority statements (e.g., available resources) and generating solutions. Discussion findings were then presented back to the wider group for more feedback/input. The process aimed to incorporate diverse perspectives and ensured that the most important and feasible improvements were identified for implementation. After the joint workshop, co‐design groups were formed to work on national/system‐level solutions generated from the joint workshop. Further details of the feedback events and joint workshop (such as the agendas, format and accompanying materials) can be found in Supporting Information S6.

### 2.7 Ethical Considerations

Ethical approval for the surveys was obtained from the Singapore Institute of Technology Review Board (SIT‐IRB: 2021176). The rest of the studies (i.e., site observations, interviews, case note reviews, feedback events and the joint workshop) were classified as a national quality improvement project by the Singapore MoH and were therefore exempt from ethical approval. The team adhered to the local standards for conducting quality improvement projects.

### 2.8 Statistical Analysis

We used mixed‐method data analyses, incorporating quantitative and qualitative methods (i.e., site observations, surveys, case note reviews, interviews and feedback events). For the site observations, surveys and case note reviews, we used descriptive statistics to summarise the data and reported categorical variables as proportions and percentages. *χ* ^2^ tests compared differences between groups, with significance at *p* < 0.05. Participant characteristics for the interviews were analysed in a similar manner. Template analysis was used to systematically code and interpret the interview data [17]. An initial coding template was developed and refined iteratively. Each interview transcript was double‐coded by two of three interviewers (K.D., S.T. and K.L.K.), with discrepancies resolved through discussion with the third interviewer. Analytical memos documented coding decisions and theme refinements. Findings were supported by illustrative participant quotations, with consistency maintained between raw data and thematic interpretations. Broad themes and sub‐themes were distinguished during reporting. Preliminary thematic summaries were presented to participants during feedback events, providing an opportunity for participant validation within the EBCD process. Priority statements were aggregated using a graph theory‐based voting system, considering importance and feasibility. Analyses were conducted using Microsoft Excel (Microsoft Corp, Redmond, Washington State, the United States), Stata (Release 14; StataCorp LP, College Station, Texas, the United States) and NVivo 10 (Lumivero, Denver, Colorado, the United States).

## 3 Results

The CRTW project involving 20 DRCs began in August 2021 and took 2.6 years to conduct. Seven of eight EBCD stages were completed, with the final celebration event set for July 2025. The project included 44 staff in site observations, 88 participants (54 clients and caregivers, and 34 staff) in interviews, 124 staff (95 AHPs and 29 rehabilitation leaders) in surveys, and 108 case note reviews. Additionally, 77 participants (27 clients and caregivers and 50 staff) attended feedback events, 38 participants (13 clients and caregivers and 25 staff) attended the joint workshop, and 60 participants (12 clients and caregivers and 48 staff) were part of the co‐design groups.

a. ***Experiences of clients, caregivers and staff in centre‐based day rehabilitation services***

**Figure 3:**
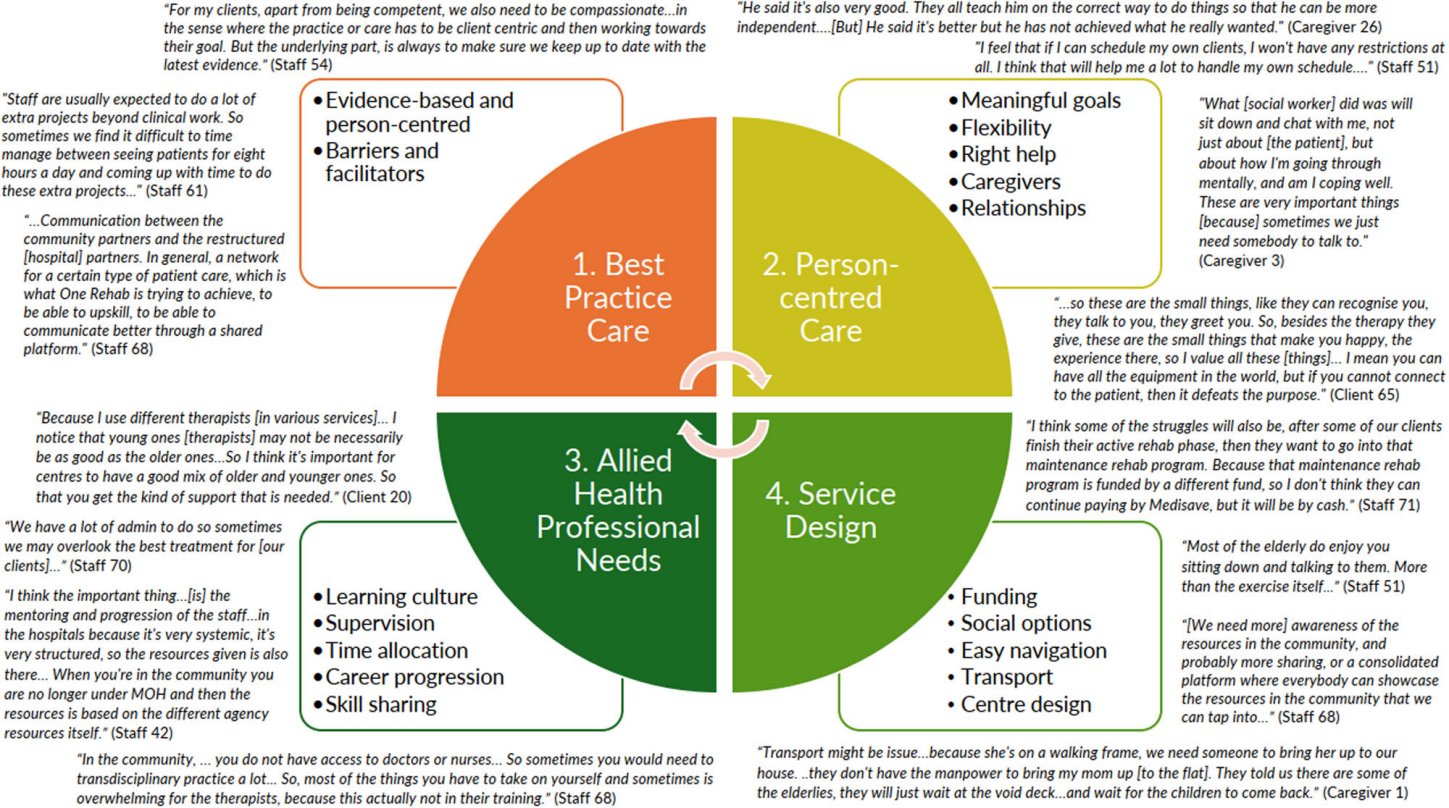
Summary of interview quotes matched to the 4 broad themes of (1) Best practice care, (2) Person‐centred care, (3) Allied Health Professional needs and (4) Service design and 17 sub‐themes.

Results from the interviews are presented in four broad themes: (1) Best practice care, (2) Person‐centred care, (3) AHP needs and (4) Service design and 17 sub‐themes to describe the experiences of clients, caregivers and staff in centre‐based day rehabilitation services. Interview quotes were matched to each of the sub‐themes (Figure 3). Additional data from the interviews (including data on the characteristics of interview participants and more quotes) can be found in Supporting Information S7.

**Figure 4:**
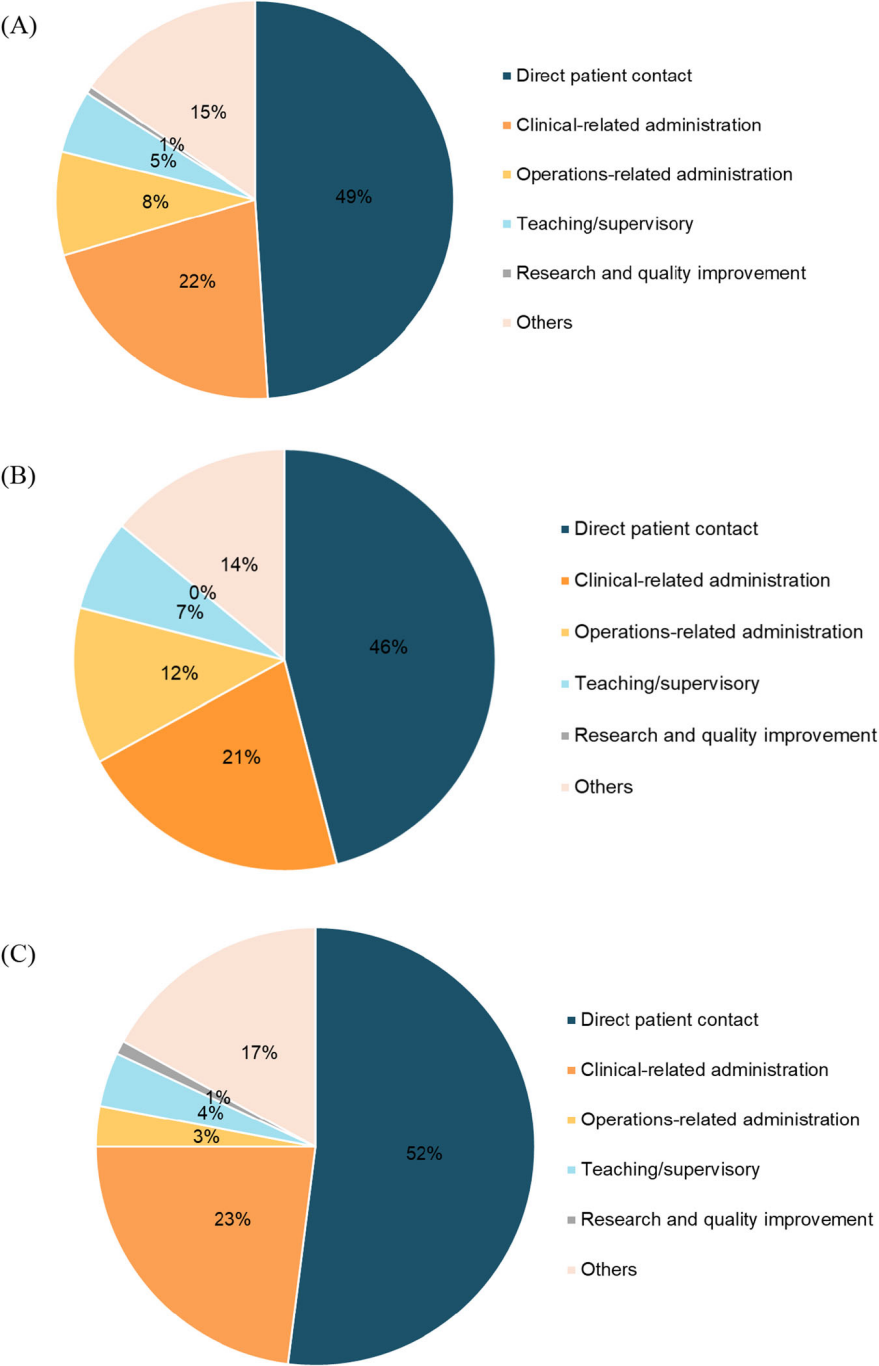
Proportion of working hours spent on activities by (A) All therapists, (B) Senior therapists and (C) Junior therapists.

**Figure 5:**
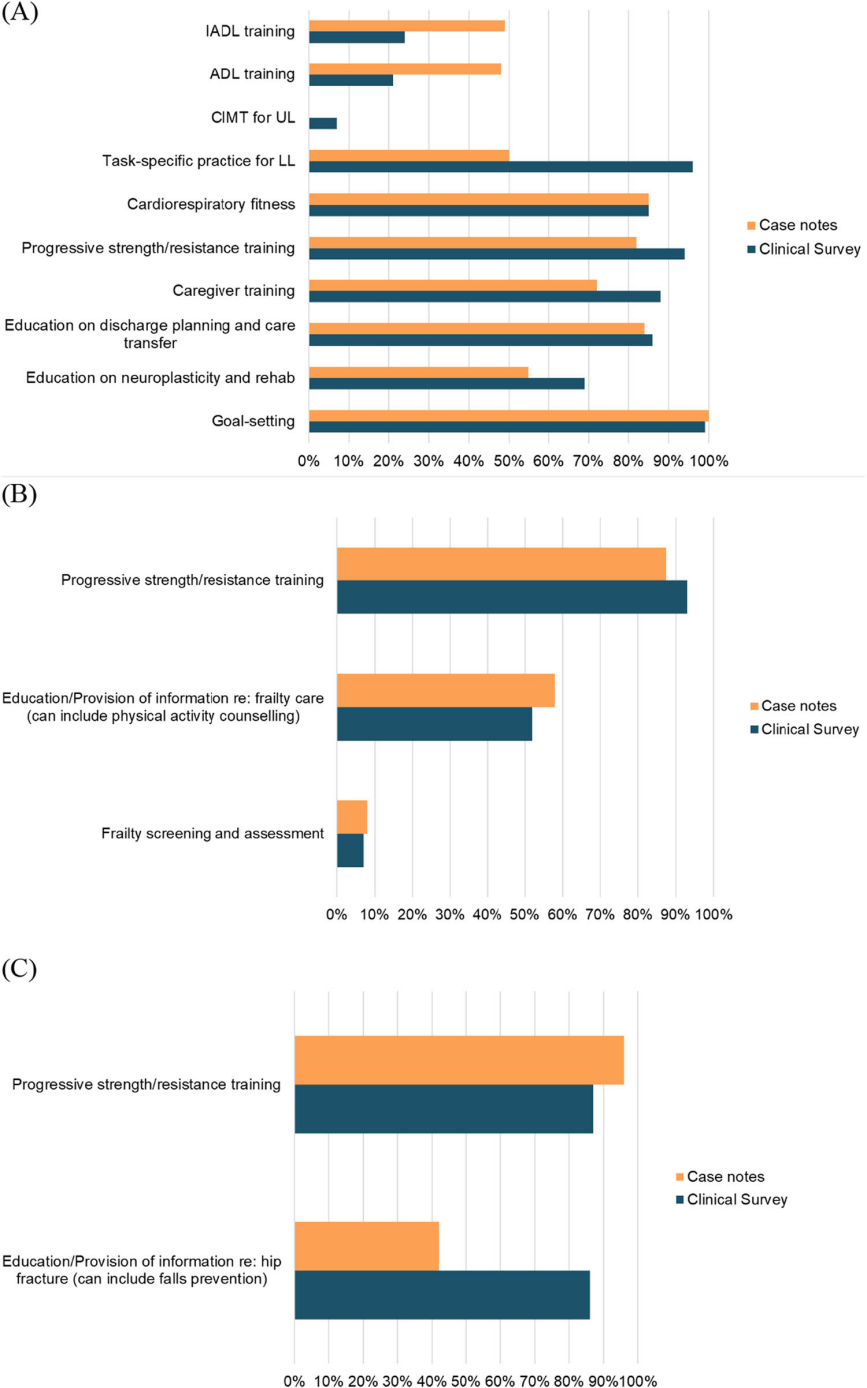
Frequency of perceived delivery and actual delivery of strongly recommended treatments for (A) Stroke, (B) Frailty and (C) Hip fracture (based on 95 clinical survey responses and 108 case note reviews). IADL, Instrumental activities of daily living; ADL, Activities of daily living; UL, Upper limb; LL, Lower limb.

**Figure 6:**
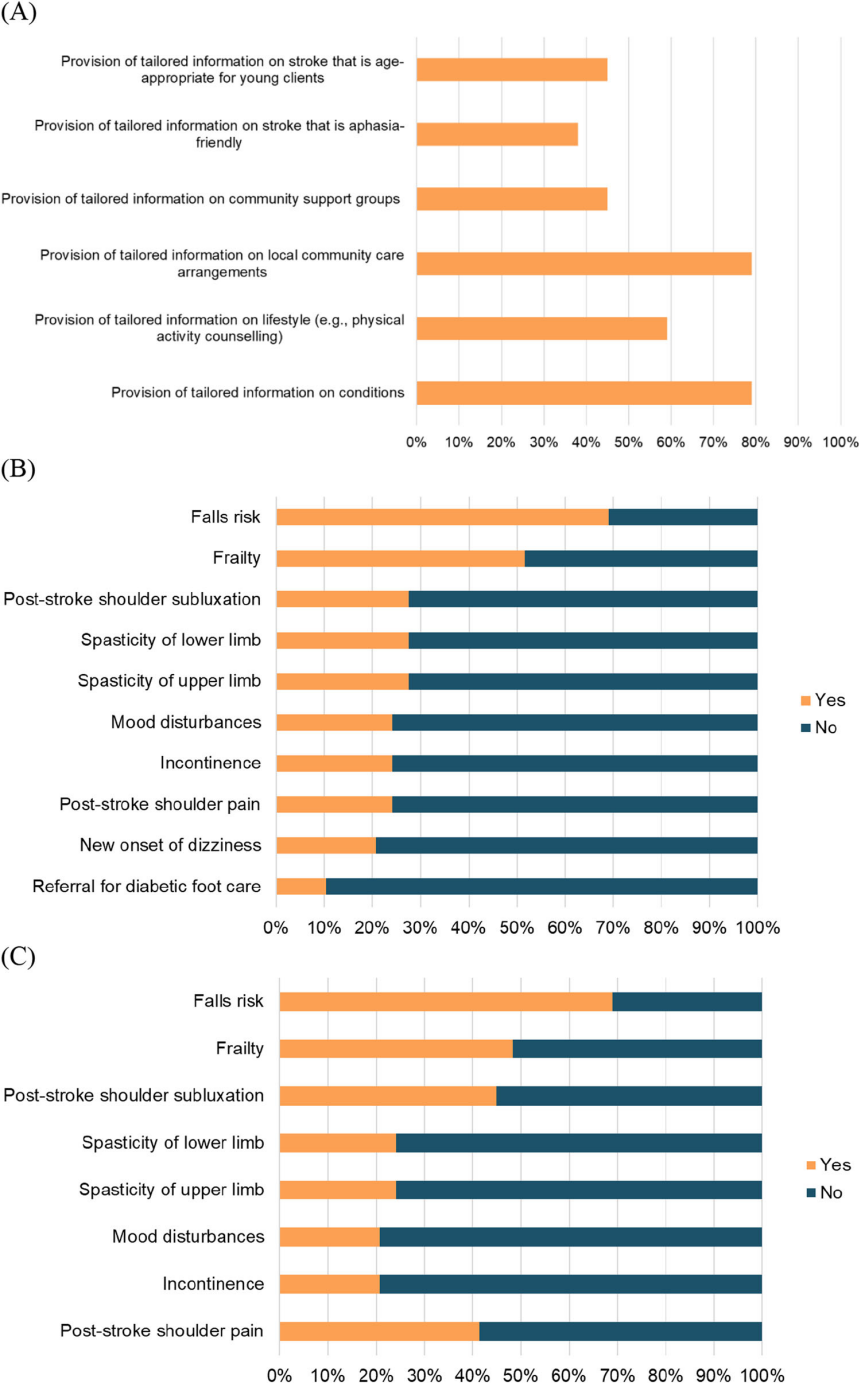
Frequency of organisational processes in terms of (A) Information provision, (B) Protocols to identify complications and (C) Protocols to manage complications (based on 29 organisational survey responses, equivalent to 29 DRCs).

1. *Best practice care:* Best practice care was defined by an evidence‐based and individualised approach, underpinned by care and compassion. Staff emphasised the need for guidelines, particularly for diverse populations in the community settings, to remain aligned with the latest research and deliver care that truly addressed each client's unique needs. Multiple factors influenced the delivery of best practice care, including the presence/absence of time, access to training and resources, coaching and senior support, and peer exchanges to share insights and reinforce skills.
2. *Person‐centred care:* Person‐centred care in rehabilitation focused on achieving meaningful goals, with clients and caregivers appreciating therapists who worked on real‐world outcomes. Therapists' flexibility to adjust their approach based on clients' individual needs contributed to their job satisfaction. However, challenges existed in providing the right help at the right time, with clients and caregivers often struggling to navigate the complex health system. Education and information resources can improve this aspect. Caregivers faced significant challenges and sacrifices in supporting their loved ones through rehabilitation. The therapeutic relationship between clients, caregivers and therapists was crucial for motivation and successful outcomes, though time constraints can hinder this process. There was a need for further professional development in rapport‐building/soft skills.
3. *AHP needs:* AHPs emphasised the importance of dedicated time within their workday for learning, reflection and planning to stay current with best practices and deliver personalised therapy. When faced with time constraints or multiple clients per session, learning took a backseat, forcing therapists to rely on repetitive activities or routine exercises to manage their workload. AHPs expressed a strong desire for mentorship, particularly from senior professionals outside their immediate work environment. Working in isolation presented challenges, particularly for junior staff in smaller organisations. Career development disparities between acute and community care settings further complicated retention, as community settings often offered limited advancement and lower compensation, which discouraged experienced practitioners. Rotational opportunities across specialisations were seen as a pathway to broaden skills and ensure a balanced career trajectory. The absence of multidisciplinary teams in smaller settings required AHPs to adopt transdisciplinary roles, which can lead to role strain. However, clients did appreciate the holistic support provided by these AHPs, acknowledging the additional functional and psychological aid integrated into their care.
4. *Service design:* Funding challenges significantly impacted the continuity and quality of rehabilitation care, as high costs for long‐term therapy often led clients to delay transitioning to maintenance programmes, prolonging active rehabilitation unnecessarily and limiting access for new clients. Compared to active rehabilitation, maintenance rehabilitation required more out‐of‐pocket costs from clients. Therapists viewed the compartmentalised funding as a barrier to comprehensive care, especially in DRCs with varying financial systems that complicate service navigation for caregivers. Social engagement was crucial in addressing client isolation, yet structured activities were limited, prompting interest in community centres offering shared‐interest activities, particularly for clients with stroke. Navigating the health system was challenging due to repetitive assessments and poor information transfer across institutions, disrupting care continuity. A centralised platform for community resources could improve information‐sharing and support. Transportation issues further complicated service accessibility, with delays leading to missed appointments and decreased client satisfaction, highlighting the need for reliable transportation. Additionally, the physical design of DRCs influenced client perceptions; caregivers noted that improvements in aesthetics could enhance motivation and foster positive attitudes towards rehabilitation.

b. ***Current state of clinical care and organisational practices in centre‐based day rehabilitation services***
  Results from the site observations, surveys and case note reviews described the current state of clinical care and organisational practices in centre‐based day rehabilitation services. Site observation data indicated that therapists spent almost half of their working hours on direct patient contact, a third of their hours on clinical‐related and operations‐related administration, and less than 10% on teaching/supervisory, research and quality improvement. There was no significant difference in terms of the proportion of time spent on activities between the senior and junior therapists (Figure 4).
  While some aspects of clinical care provided were in line with international clinical practice guidelines (Supporting Information S1) (e.g., progressive strength/resistance training for stroke, frailty and hip fracture, cardiorespiratory fitness and goal setting for stroke), some interventions with strong recommendations were not consistently delivered (e.g., instrumental activities of daily living training and activities of daily living training, and constraint‐induced movement therapy for stroke, and frailty screening and assessment). These were gained from the surveys and case note reviews and allowed us to capture congruence between perceived delivery and actual delivery of guideline‐based care (Figure 5). Of importance to note is that results were based on what were reported by therapists (from the surveys) and what were recorded in the case notes (from the case note reviews). Although there were reports and records of guideline‐based care, the quality of care provided might not be optimal. For example, while 99% of survey responses indicated often/always doing goal setting and 100% of case notes documented the presence of goal setting, it was not always done well, as evidenced by the interview quotes under the sub‐theme of meaningful goals and broad theme of person‐centred care (Figure 3 and Supporting Information S7).
  Some clients received education on their rehabilitation care and journey, and this can be encouraged so that clients receive the right help at the right time. Awareness, access and availability of education/information resources can be considered. These resources can also include ready‐made information, referral processes (e.g., for return to work/driving) and protocols for complications. Surveys and case note reviews indicated variation in the frequency of education/provision of information regarding care on stroke, frailty and hip fracture, while less than 50% of DRCs reported the provision of tailored stroke information that is age‐appropriate for young clients and aphasia‐friendly or contained details on community support groups (Figure 6). While more than 50% of DRCs had protocols to identify and manage fall risk, protocols on identifying and managing other complications (e.g., frailty, post‐stroke shoulder subluxation and shoulder pain, spasticity, mood disturbances and incontinence) were not available (Figure 6). These are potentially areas of care that can be easy targets for quick solutions. Note that the most frequently documented complication in the case notes was pain (25/108; 23%) and fatigue (15/108; 14%) for all conditions, and spasticity for stroke (5/44; 11%). Considering the shortage of AHPs in the community, it was encouraging to see that skills sharing was currently being practised with most AHPs reporting either practising skills sharing randomly or in a well‐defined manner (81/95; 85%) and most rehabilitation leaders reporting similarly (23/29; 79%). To improve the practice of skills sharing, sites can explore developing or exchanging information regarding processes, such as structured training, competency checklists for tasks, or a list of tasks to be skill‐shared across professional boundaries, since only 60% of DRCs reported having such processes in place. Additional data from the surveys and case note reviews (including data on skills sharing and the characteristics of survey respondents) can be found in Supporting Information S8.
c. ***Areas of prioritisation and consensus for improvement by clients, caregivers and staff***

**Figure 7:**
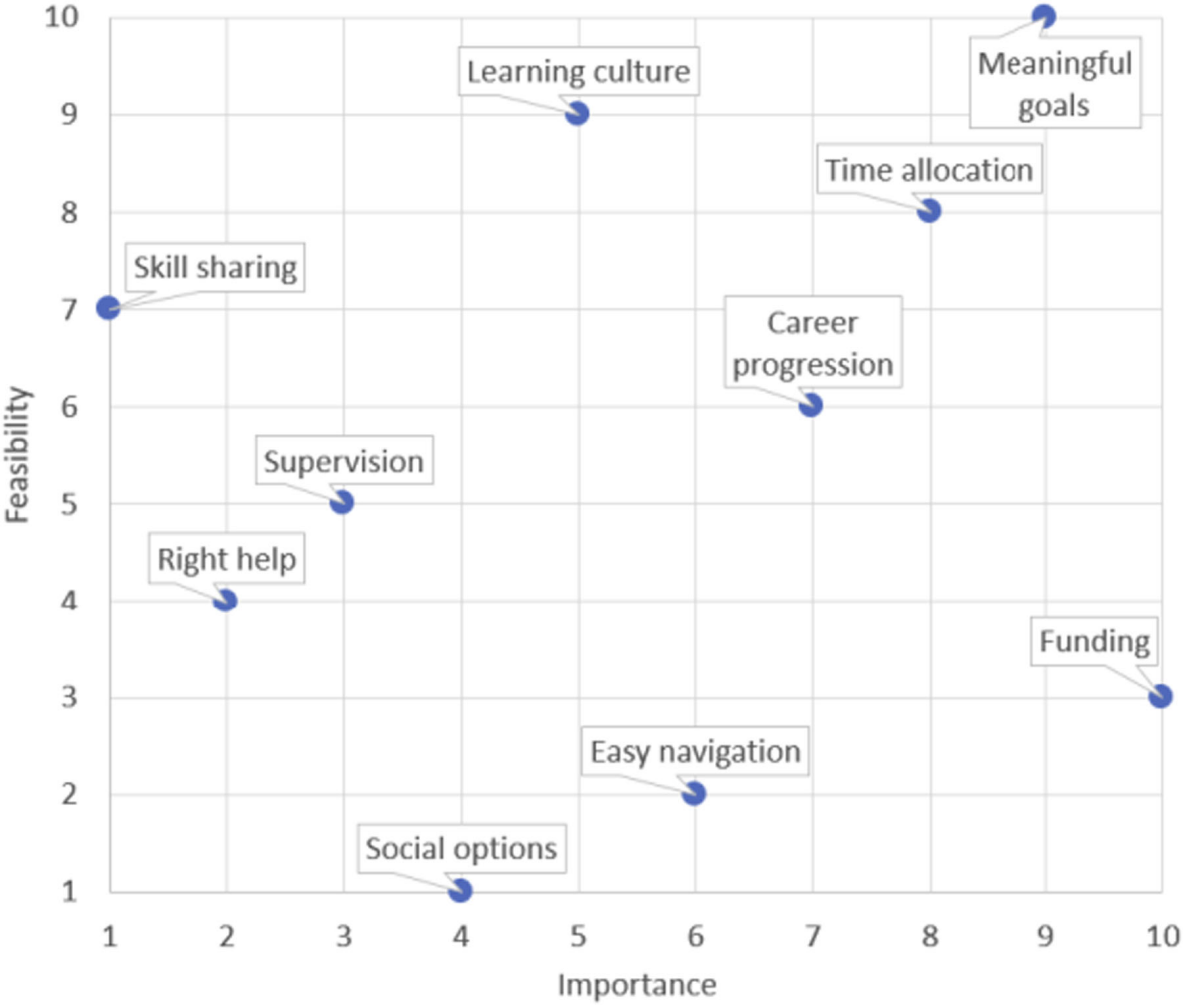
Scatter plot of aggregated ratings of the shortlisted 10 priority statements by staff (statements in the top right quadrant were deemed to be most important and most feasible to solve based on current resources).

Frequency of priority statements chosen in the top five by clients, caregivers and staff is presented in Table 1, while results of the graph theory‐based voting system are shown in Figure 7. Funding and meaningful goals were the top priority statements selected by all, followed by relationships, flexibility and transport by clients and caregivers, and time allocation, career progression and learning culture by staff. Additionally, meaningful goals, time allocation and career progression were rated by staff to be the most important and feasible areas to focus on. During the joint workshop, priority statements that were discussed in‐depth included meaningful goals, right help (tailored information), skill sharing, learning culture, supervision, easy navigation, flexibility and social options. Following the feedback events and joint workshop, three working groups were formed. The first group ‘Clinical Practice Guidelines’ focused on the development of stroke rehabilitation guidelines and resources to guide meaningful goal‐setting practices and care with strong recommendations. The second group ‘Professional Development’ worked on a workplace learning framework which defined the roles, needs and opportunities for the ongoing professional development of therapists to enhance person‐centred care through structured supervision, coaching/mentoring, education and skills‐sharing opportunities. The third group ‘Community rehabilitation and support services’ focused on remodelling the pain points at a systems and policy level, with the recommendation of a national benchmark for referral processing time for admission to outpatient rehabilitation and a service dashboard for agencies to monitor capacity for community service providers and guidance on appropriate staffing ratios in centres.

**Table 1:** Frequency of priority statements chosen in the top five by clients, caregivers and staff.

[Frequency rank] Clients and caregivers*	[Frequency rank] Staff
[1] Funding	[1] Meaningful goals
[2] Relationships	[2] Funding
[3] Flexibility	[3] Time allocation
[3] Meaningful goals	[4] Career progression
[4] Transport	[5] Learning culture
[5] Easy navigation	[6] Easy navigation
[6] Learning culture	[7] Social options
[7] Supervision	[7] Flexibility
[7] Time allocation	[8] Right help
[7] Social options	[9] Supervision
[7] Right help	[10] Centre design
[8] Caregivers	[11] Transport
[9] Centre design	[12] Caregivers
[10] Skill sharing	[13] Skill sharing
	[14] Relationships

*Note:* A small frequency rank indicates top ranking (i.e., more people selected the priority statement in the top five), while a big frequency rank indicates bottom ranking (i.e., fewer people selected the priority statement in the top five). Similar rank numbers indicate the same number of people selecting the priority statement in the top five.

* Only 14 priority statements as staff requested for career progression not to be considered by clients and caregivers.

## 4 Discussion

We applied the EBCD methodology to foster collaboration among clients, caregivers and staff in transforming community‐based rehabilitation services. To our knowledge, our study is the first to employ EBCD on a national level to highlight the experiences of clients, caregivers and staff in multiple sites/rehabilitation centres and co‐design system‐level solutions. Over 2.6 years, we engaged 80+ clients/caregivers and 250 staff from 20 DRCs in the EBCD process. Data analysis revealed four themes: (1) Best practice care, (2) Person‐centred care, (3) AHP needs and (4) Service design. Person‐centred care was desired but hindered by scheduling and turnover. Care was partially aligned with guidelines, with inconsistent delivery of some interventions. Site observations showed potential for more time to be allocated to direct care, teaching and research. New models were sought, including social connections beyond DRCs and clearer rehabilitation criteria. Three workgroups formed to develop system‐level solutions in the form of stroke rehabilitation guidelines, workplace learning framework and process transformation.

Over the years, EBCD has been largely used for quality/service improvement [10]. We discussed two issues often highlighted in EBCD studies: cohort retention and power dynamics between all stakeholders [10]. Similar to previously published EBCD studies, we faced issues with cohort retention, with less than half of the clients and caregivers returning for the joint workshop after the feedback events, and different participants joining during the interviews, feedback events and joint workshop. However, considering the breadth of the work across multiple sites and involvement of clients with different conditions, we saw this as an enhancement of the process with the diverse experiences and perspectives shared [18] and a better reflection of the current community‐based rehabilitation landscape. We made several efforts to balance the power dynamics between all stakeholders (including that between staff and CAHOO). All stages of the EBCD process were optional, surveys to staff were anonymous, and clients, caregivers and staff were allowed to vote for their top five priority statements independently. Interestingly, when staff requested for ‘career progression’ not to be voted upon by clients and caregivers, it was because of concern that ‘career progression’ might not be prioritised if clients and caregivers did not select it to be one of the top five priority areas, demonstrating the acknowledgement of power (or perceived power) accorded to clients and caregivers. In the next phase, we have continued to engage stroke survivors and caregivers in the ‘Clinical Practice Guidelines’ workgroup, where we are adapting the Australian Stroke Foundation guidelines that have been developed using the GRADE methodology (Grading of Recommendations, Assessment, Development and Evaluation) [19, 20]. As consumer preference is one of the four factors that guide the strength of recommendations in the guidelines, some recommendations have been changed based on the input of stroke survivors and caregivers (e.g., from ‘weak for’ to ‘strong for’). We believe that the use of such power‐sharing structures and collective ownership, as mentioned in prior studies [21, 22], has helped to balance out the power dynamics between all stakeholders.

This comprehensive exercise allowed us to capture an accurate picture of the current community rehabilitation landscape and highlighted what an ideal experience of community‐based rehabilitation service would look like. It was apparent that the experiences of clients, caregivers and staff were influenced by a complex interaction of factors. Not only was the use of multiple sources of data needed to provide a clearer picture, but solutions were likely going to be most effective when targeted at multiple levels (i.e., individual, organisation and population levels). For example, meaningful goal setting, often a strong recommendation in guidelines and central to person‐centred care, was hard to capture. Although it was often/always done, as evidenced by survey responses and case note reviews, interview data revealed that it was not always done well. Additionally, the ability to provide person‐centred care depended too on the clinician's expertise (e.g., soft skills and technical knowledge) and the flexibility of the service structure (e.g., community ambulation might not be possible if there are back‐to‐back time slots for clients to be seen at the centre). Without solutions targeted at the individual level and/or organisational level, such as structured supervision and coaching/mentoring, and flexible therapy time slots, working on meaningful goals might not be feasible. We encourage staff from the centres to review our study findings and look at what they can improve within their own organisations, as well as to use the generated data to guide future quality improvement and research projects. We also encourage cross‐sectoral partnerships between the acute sector, ILTC sector and academic institutions as this will be useful for facilitating the cross‐exchange of expertise, knowledge and skills and help test interventions and expedite data collection and analysis for future research work.

Our solutions had been focused on a systems/population level in the form of stroke rehabilitation guidelines, workplace learning framework and process transformation. Since the commencement of the project, several national initiatives had also been introduced, which were synergistic with our solutions. These included the implementation of continuing professional education (CPE) (where AHPs are required to participate in CPE activities and acquire a certain number of CPE points for renewal of their practising certificates [23]), Support GoWhere (a comprehensive online platform/centralised hub where individuals and families can easily find and understand various government assistance schemes and support services available to them in Singapore) [24], and the network of active ageing centres formed to facilitate social prescription as part of the HealthierSG plan to promote preventive care [25]. These national initiatives will no doubt expedite solutions targeted at AHP needs (e.g., priority statements ‘learning culture’ and ‘time allocation’), person‐centred care (e.g., priority statements ‘right help’) and service design (e.g., priority statements ‘social options’ and ‘easy navigation’). Whether our solutions and these national initiatives are successful in improving the experiences of community‐based rehabilitation care in Singapore remains to be seen in the outcomes of future quality improvement and research projects.

### 4.1 Strengths and Limitations

The strengths of our study included the novel use of EBCD to develop healthcare policies, the engagement of multiple stakeholders across the ILTC sectors, and the collection of data to facilitate future research and/or quality improvement projects. Despite the strengths of our study, there were three limitations. First, we took longer than other EBCD studies, lasting 20 months for data collection and analysis, due to the complexity of the healthcare system, involvement of multiple stakeholders, including government agencies, and the triangulation of data from multiple sources. Sustaining the interest and engagement of the champions and stakeholders was challenging. However, we found that regular engagement via meetings and workshops, dissemination of EBCD results via a 40‐min video, and the return of site‐specific case note review results to organisations for their own quality improvement projects helped to mitigate disengagement. Second, we did not have people with lived experience as team members in the CRTW workgroup. Giving clients and caregivers a more active role in the research process right from the start can help to ensure a better balance of the power dynamics and aid knowledge mobilisation [22]. Although steps were taken in our study to ensure clients and caregivers' opinions were heard (e.g., separate client and caregiver feedback events, trigger film only consisting of client and caregiver narratives, and trained facilitators to support clients and caregivers in small group discussions), this can be further improved by providing training to clients and caregivers [26], and setting up and liaising with networks of Patient and Public Involvement groups [27, 28]. Third, the clinical survey had a low response and was not completed by 19% (22/117) of participants who attempted the clinical survey, likely due to its length. For future evaluations of clinical care, extracting key data from electronic documentation may streamline the process [29].

## 5 Conclusions

EBCD was implemented as part of a national quality improvement project in Singapore, marking the first government‐led co‐design effort to enhance community rehabilitation. Further interventions at the organisational and individual levels are needed to ensure the successful implementation of the stroke rehabilitation clinical practice guidelines, the workplace learning and support framework for allied health, and process transformation.

## Author Contributions

**Shamala Thilarajah:** conceptualisation, methodology, project administration, investigation, formal analysis, visualisation, data curation, validation, software, writing – original draft, writing – review and editing. **Karina Dancza:** methodology, investigation, formal analysis, data curation, validation, software, writing – original draft, writing – review and editing. **Zhen Zhen Chen:** methodology, project administration, investigation, formal analysis, visualisation, data curation, validation, software, writing – review and editing. **Clara X. Q. Wong:** methodology, project administration, investigation, formal analysis, visualisation, data curation, validation, software, writing – review and editing. **Clement C. Yan:** validation, writing – review and editing. **Susan Niam:** conceptualisation, supervision, funding acquisition, resources, methodology, validation, writing – review and editing. **Yee Sien Ng:** conceptualisation, supervision, funding acquisition, resources, methodology, validation, writing – review and editing. **Elizabeth Lynch:** methodology, writing – review and editing. **Leonid Churilov:** methodology, writing – review and editing. **Wei Kang Tan:** investigation, formal analysis, data curation, validation, software, writing – review and editing. **Emelin Tan:** methodology, project administration, investigation, formal analysis, visualisation, data curation, validation, software, writing – review and editing. **Li Khim Kwah:** conceptualisation, methodology, project administration, investigation, formal analysis, visualisation, data curation, validation, software, writing – original draft, writing – review and editing.

## Consent

Informed consent was obtained from all participants involved in this quality improvement project, as it aligned with ethical principles and institutional guidelines for such initiatives.

## Conflicts of Interest

The authors declare no conflicts of interest.

## Supporting information


**Supporting Information 1.** Resources used to guide methods for project.

**Supporting Information 2.** Site observations – Classification of observed activity.

**Supporting Information 3.** Interviews – Questions for staff, clients and caregivers.

**Supporting Information 4.** Surveys – Clinical and organisational surveys.

**Supporting Information 5.** Case note reviews – Template.

**Supporting Information 6.** Details of the feedback events and joint workshop.

**Supporting Information 7.** Additional data from interviews.

**Supporting Information 8.** Additional data from surveys and case note reviews.

## Data Availability

The authors have nothing to report.
