# Supplementary material for: Transforming Community‐Based Rehabilitation Services: A National Redesign Using Experience‐Based Co‐Design
Source: Health Expect. 2025 Jun 23;28(3):e70330. doi: 10.1111/hex.70330 (PMC12183464; doi:10.1111/hex.70330)
Supplement: Supplementary file 1 — Supporting Information 1. Resources used to guide methods for project. [file HEX-28-e70330-s004.pdf]

Supplementary materials 1. Resources used to guide methods for project

We referred to the following materials to guide our methods for EBCD and templates for feedback events, joint workshop, time motion study, interviews, surveys and case note reviews: a) EBCD toolkits from the Australian Healthcare and Hospitals Association (AHHA) (1) and the Kings Fund, the Point of Care Foundation (2), b) published time-motion studies (3, 4), c) published EBCD studies (5, 6), d) systematic reviews of clinical practice guidelines (CPGs) on stroke, frailty and hip fracture (7-9) (to identify high quality CPGs), e) high quality CPGs on stroke (10), frailty (11) and hip fracture (12, 13), region-specific CPG on frailty (14), consensus guidelines and systematic review of screening and assessment tools for frailty (15, 16), systematic reviews of interventions to prevent falls and improve mobility for hip fracture (17-19) (to extract recommended assessments and treatments for stroke, frailty and hip fracture), and f) the Stroke Foundation National Stroke Audit – Rehabilitation Services Report 2020 (20). In addition, we also referred to the Ministry of Health service requirement document for centre based care in Singapore (21) to ensure the questions in the surveys and case note reviews were contextualised to the local setting.

The guidelines and systematic reviews guided the assessments and treatments listed in the clinical survey. To ensure relevance, we only extracted assessments and treatments that could be delivered within the setting of the Day Rehabilitation Centres (DRCs) in Singapore. We referred to CPGs on frailty to guide questions on clinical practices as there was no CPG specific to deconditioning, considering deconditioning was a broad term which included a multitude of conditions. In addition, we also referred to consensus guidelines and systematic reviews of assessment tools for frailty, and systematic reviews on interventions and exercises to prevent falls and improve mobility in hip fracture as details such as screening and assessment tools for frailty and interventions for frailty and hip fracture were missing from CPGs.

References

1. AHHA. Experience based co-design: a toolkit for Australia Australia: Australian Healthcare and Hospitals Association (AHHA); 2017 [Available from: https://ahha.asn.au/experience-based-co-design-toolkit [Accessed 29 August, 2021].

2. POC. EBCD: Experience-based co-design toolkit United Kingdom: The Kings Fund, The Point of Care Foundation; 2016 [Available from: https://www.pointofcarefoundation.org.uk/resource/experience-based-co-design-ebcd-toolkit/ [Accessed 29 August, 2021].

3. Clarke DJ, Burton LJ, Tyson SF, Rodgers H, Drummond A, Palmer R, et al. Why do stroke survivors not receive recommended amounts of active therapy? Findings from the ReAcT study, a mixed-methods case-study evaluation in eight stroke units. Clinical Rehabilitation. 2018;32(8):1119-32.

4. Schneider A, Williams DJ, Kalynych C, Wehler M, Weigl M. Physicians' and nurses' work time allocation and workflow interruptions in emergency departments: a comparative time-motion study across two countries. Emergency Medicine Journal. 2021;38(4):263-8.

5. Jones F, Gombert-Waldron K, Honey S, Cloud G, Harris R, Macdonald A, et al. Using co-production to increase activity in acute stroke units: the CREATE mixed-methods study 2020;8(35).

6. van Deventer C, Robert G, Wright A. Improving childhood nutrition and wellness in South Africa: involving mothers/caregivers of malnourished or HIV positive children and health care workers as co-designers to enhance a local quality improvement intervention. BMC Health Services Research. 2016;16(a):358.

7. Jolliffe L, Lannin NA, Cadilhac DA, Hoffmann T. Systematic review of clinical practice guidelines to identify recommendations for rehabilitation after stroke and other acquired brain injuries. BMJ Open. 2018;8(2):e018791.

8. Mehta P, Lemon G, Hight L, Allan A, Li C, Pandher SK, et al. A systematic review of clinical practice guidelines for identification and management of frailty. The Journal of Nutrition, Health and Aging. 2021;25(3):382-91.

9. Gimigliano F, Liguori S, Moretti A, Toro G, Rauch A, Negrini S, et al. Systematic review of clinical practice guidelines for adults with fractures: identification of best evidence for rehabilitation to develop the WHO's Package of Interventions for Rehabilitation. Journal of Orthopaedics and Traumatology. 2020;21(1):20.

10. SF. Clinical guidelines for stroke management Australia: Stroke Foundation; 2019 [Available from: https://informme.org.au/en/Guidelines/Clinical-Guidelines-for-Stroke-Management [Accessed 09 September, 2020].

11. Dent E, Morley JE, Cruz-Jentoft AJ, Woodhouse L, Rodriguez-Manas L, Fried LP, et al. Physical frailty: ICFSR international clinical practice guidelines for identification and management. The Journal of Nutrition, Health and Aging. 2019;23(9):771-87.

12. NICE. Hip fracture: Management. The United Kingdom NICE; 2020 [Available from: https://www.nice.org.uk/guidance/cg124 [Accessed 09 September 2020].

13. ANZFHR. Australian and New Zealand guideline for hip fracture care - Improving outcomes in hip fracture management of adults. New South Wales, Sydney, Australia: Australian and New Zealand Hip Fracture Registry (ANZHFR) Steering Group; 2014 [Available from: https://anzhfr.org/guidelines-and-standards/ [Accessed 09 September 2020].

14. Dent E, Lien C, Lim WS, Wong WC, Wong CH, Ng TP, et al. The Asia-Pacific clinical practice guidelines for the management of frailty. Journal of the American Medical Directors Association. 2017;18(7):564-75.

15. Ruiz JG, Dent E, Morley JE, Merchant RA, Beilby J, Beard J, et al. Screening for and managing the person with frailty in primary care: ICFSR consensus guidelines. The Journal of Nutrition, Health and Aging. 2020;24(9):920-7.

16. Clegg A, Rogers L, Young J. Diagnostic test accuracy of simple instruments for identifying frailty in community-dwelling older people: a systematic review. Age and Ageing. 2015;44(1):148-52.

17. Hopewell S, Adedire O, Copsey BJ, Boniface GJ, Sherrington C, Clemson L, et al. Multifactorial and multiple component interventions for preventing falls in older people living in the community. Cochrane Database of Systematic Reviews. 2018;7:CD012221.

18. Sherrington C, Fairhall NJ, Wallbank GK, Tiedemann A, Michaleff ZA, Howard K, et al. Exercise for preventing falls in older people living in the community. Cochrane Database of Systematic Reviews. 2019;1:CD012424.

19. Diong J, Allen N, Sherrington C. Structured exercise improves mobility after hip fracture: a meta-analysis with meta-regression. British Journal of Sports Medicine. 2016;50(6):346-55.

20. SF. National Stroke Audit - Rehabilitation Services Report 2020 Australia: Stroke Foundation (SF); 2020 [Available from: https://informme.org.au/stroke-data/Rehabilitation-audits [Accessed 19 May 2021].

21. MOH. Home and centre-based care service requirements Singapore: Ministry of Health (MOH), Government of Singapore; 2021 [Available from: https://www.moh.gov.sg/resources-statistics/guidelines/home-and-centre-based-care-service-requirements [Accessed 29 August, 2021].
