# Supplementary material for: Transforming Community‐Based Rehabilitation Services: A National Redesign Using Experience‐Based Co‐Design
Source: Health Expect. 2025 Jun 23;28(3):e70330. doi: 10.1111/hex.70330 (PMC12183464; doi:10.1111/hex.70330)
Supplement: Supplementary file 2 — Supporting Information 2. Site observations – Classification of observed activity. [file HEX-28-e70330-s006.pdf]

Supplementary materials 2. Site observations – Classification of observed activity

Activity	Details of activity
Clinical	• Client contact – Time spent with client physically<br>• Tele-consult – Time spent with client virtually<br>• Prepare/set-up equipment – Time spent setting up computer, environment, therapeutic equipment<br>• Call family – Time spent on telephone with family members or caregiver to seek information, update on progress, discuss goals/care plans.<br>• Handover cases to therapy associates – Time spent speaking to therapy associates to hand over patients or receive handover from therapy associates
Clinical-related administration	• Clinical documentation – Time spent doing documentation<br>• Read case notes – Time spent reading case notes<br>• Multi-disciplinary rounds - Time spent attending multi-disciplinary meetings (physical or virtual)<br>• Family conference – Multidisciplinary meeting with family (physical or virtual)<br>• Check for referrals – Time spent checking for referrals<br>• Write client memos/fill up application forms – Time spent writing memo (e.g., memo to other healthcare professional, memo for purchase of walking aids) or time spent on seniors' mobility and enabling fund (SMF) application<br>• Talk to doctor/nurse/other health care professionals – Time spent communicating with other health care professionals (physical or virtual) for the purpose of clinical care
Operations-related administration	• On the telephone – Time spent communicating with other staff for matters not related to client (e.g., discussion on manpower, projects)<br>• Meetings – Time spent attending physical or virtual meetings for matters not related to client<br>• Stats recording – Time spent doing daily workload stats<br>• Roll call – Time spent doing roll call<br>• Billing – Time spent doing billing<br>• Audits – Time spent conducting audits on client management or documentation<br>• All other admin work – Time spent doing any other work that is not related to client (e.g., leave application, projects)
Teaching/ Supervisory	• Train junior staff – Time spent seeing clients together with junior staff for mentoring purpose<br>• Case discussion with junior staff – Time spent discussing cases with junior staff for mentoring purpose<br>• Case discussion with senior staff – Time spent discussing cases with senior staff for mentoring purpose<br>• AHPC log forms (for AHPC supervisees) – Time spent by AHPC supervisee to enter AHPC log forms<br>• Prepare AHPC forms (for AHPC supervisors) – Time spent by AHPC supervisor to complete AHPC forms for supervisees

	• Attend inservices/courses/e-learning – Time spent attending professional education activities (physical or virtual)<br>• Conduct inservices/courses/e-learning – Time spent delivering professional education activities (physical or virtual)
Research and quality improvement	• Research related work – Time spent doing any research related work<br>• Quality improvement related work – Time spent doing any quality improvement related work
Others	• Toilet break – Time taken to walk to location<br>• Meal break – Time taken to walk to location<br>• Water break – Time taken to walk to location
