# Supplementary material for: Transforming Community‐Based Rehabilitation Services: A National Redesign Using Experience‐Based Co‐Design
Source: Health Expect. 2025 Jun 23;28(3):e70330. doi: 10.1111/hex.70330 (PMC12183464; doi:10.1111/hex.70330)
Supplement: Supplementary file 3 — Supporting Information 3. Interviews – Questions for staff, clients and caregivers. [file HEX-28-e70330-s003.pdf]

Supplementary materials 3. Interviews – Questions for staff, clients and caregivers

Interview Script for Staff

Thank you for taking time out to participate in this study. The aim of this interview is to help us understand the experiences of staff providing community rehabilitation in Singapore. The project is part of a wider piece of work that aims to enhance the delivery of community rehabilitation services. We are using an approach called experience-based co-design (EBCD), where patients and staff work together to co-design services to improve patient and staff experiences. Today, we wish to understand your experience of delivering the community rehabilitation service. With your consent, we would like to audio record the interview. You will have the opportunity to review your interview transcript if you wish. Is this OK for you? Do you have any questions before we start?

<u>Participant characteristics</u>

1. What is your age? _____
2. What is your gender? Male/Female
3. What is your ethnicity? Chinese/Indian/Malay/Caucasian/Other
4. What is your current role in the organisation? PT/OT/SLT/Other, please specify:_____
5. What is your highest education qualification? PhD/Doctorate/Bachelor's degree/Diploma/ Other, please specify:_____
6. How many years of practice do you have as a health care professional?
7. How many years have you worked as a health care professional in this organisation? (Please only include the years where you are engaged in full-time, part-time or locum work.)

<u>Background</u>

8. Tell me what your role is in the service and the kind of work that you do here.

<u>Staff experience of working in the community rehabilitation service</u>

9. Can you share some positive experiences of working in this service?
10. Can you share any areas you would like to change to enhance your experiences of working in this service?
    (Prompt: What is it like working here? Can you share some memorable experiences? The experiences can be related to an encounter with a patient or staff at work.)

[Interviewer to summarise list of positive and negative experiences.]

<u>Staff perspectives on best practice/care</u>

11. What does best practice/care for your clients mean to you?
    (Prompt: What is your perspective on best practice/care for your clients? Other than alignment with clinical practice guidelines, how else does best practice/care look to you? Are you aware of any best practices overseas?)
12. What do you think helps you in delivering best practice/care for your clients?
    (Prompt: What are some facilitators that you face when trying to deliver best practice/care for your clients?)
13. What do you think limits you from delivering best practice/care for your clients?
    (Prompt: What are some barriers that you face when trying to deliver best practice/care for your clients?)

[Interviewer to summarise perspectives on best practice/care, and barriers and facilitators to delivering best practice/care.]

<u>Transforming the community rehabilitation service</u>

14. What do you think would help to improve staff experiences in this community rehabilitation service?
    (Prompt: Can you name three things/factors that you feel will improve your experience and the experience of other staff working in this community rehabilitation service?)
15. What do you think patients would identify as things that would help to improve patient experiences in this community rehabilitation service?
    (Prompt: Can you name three things/factors that you feel will improve patient experience in this community rehabilitation service?)

[Interviewer to summarise solutions.] Thank you so much for your time and responses.

Interview Script for Patients and Carers (who used community rehabilitation services)

Thank you for taking time out to participate in this study. The aim of this interview is to help us understand the experiences of patients receiving community rehabilitation in Singapore. The project is part of a wider piece of work that aims to enhance the delivery of community rehabilitation services. We are using an approach called experience-based co-design (EBCD), where patients and staff work together to redesign services to improve patient and staff experiences. Today, we wish to understand your experience of receiving the community rehabilitation service. With your consent, we would like to video record the interview. If you do not wish to be video recorded and only allow for audio recording, please let me know. If you consent to be video recorded, we will use a few short clips of the video to share in the next stage of the project where we work with patients, carers and staff to brainstorm solutions to improve the community rehabilitation service. We will show you the clips of the video to be shared, and you will be able to say yes or no to us sharing it. You will also have the opportunity to review your audio and/or interview transcript if you wish. Is this OK for you? Do you have any questions before we start?

Participant characteristics (for Patient)

1. What is your age? _____
2. What is your gender? Male/Female
3. What is your ethnicity? Chinese/Indian/Malay/Caucasian/Other, please specify:_____
4. What is your marital status? Single/Married/Divorced/Other, please specify:_____
5. What is your highest education qualification? PhD/Doctorate/Bachelor's degree/Diploma/ Other, please specify:_____
6. How many weeks of community rehabilitation service did you access? _____ weeks
7. Which of the following health care professionals did you see as part of the community rehabilitation service? Doctor/Nurse/Physiotherapist/Occupational Therapist/Speech and Language Therapist/Social Worker/Psychologist/Therapy Assistant/Other, please specify:_____

Participant characteristics (for Carer)

8. I am the __________ of person who has accessed community rehabilitation services. Spouse/Parent/Sibling/Others, please specify:_____
9. What is your age? _____
10. What is your gender? Male/Female
11. What is your ethnicity? Chinese/Indian/Malay/Caucasian/Other, please specify:_____
12. What is your marital status? Single/Married/Divorced/Other, please specify:_____
13. What is your highest education qualification? PhD/Doctorate/Bachelor's degree/Diploma/ Other, please specify:_____
14. What type of housing do you live in? HDB lift landing/HDB non-lift landing/ Private/Nursing home

Background:

15. Tell me about yourself and how you/person whom you care for came to use the community rehabilitation service.

Patient and carer experiences of using the community rehabilitation service

15. Can you share some positive experiences of your care at the community rehabilitation service? Can you give some examples?
16. Can you share some any areas you would like to change to make your experiences of your care at the community rehabilitation service better? Can you give some examples?
    (Prompt: What is it like receiving care here? Can you share some memorable experiences? The experiences can be related to an encounter with a patient or staff at work.)
17. Can you describe a typical session for you at the community rehabilitation service?
    (Prompt: Can you take me step by step through what a usual session would look like? If I was a fly on the wall, what would I see you spending most of your time doing?)

[Interviewer to summarise list of positive and negative experiences, and aspects of a typical session.]

Transforming the community rehabilitation service

18. What do you think would help to improve patient experiences in this community rehabilitation service?
    (Prompt: Can you name three things/factors that you feel will improve your experience and the experience of other patients in this community rehabilitation service? What would a great session look like?)

[Interviewer to summarise solutions.] Thank you so much for your time and responses.

Interview Script for Patients and Carers (who were referred, but did not use community rehabilitation services)

Thank you for taking time out to participate in this study. The aim of this interview is to help us understand the experiences of patients receiving community rehabilitation in Singapore. The project is part of a wider piece of work that aims to enhance the delivery of community rehabilitation services. We are using an approach called experience-based co-design (EBCD), where patients and staff work together to redesign services to improve patient and staff experiences. Today, we wish to understand your reasons for not using the community rehabilitation services. With your consent, we would like to video record the interview. If you do not wish to be video recorded and only allow for audio recording, please let me know. If you consent to be video recorded, we will use a few short clips of the video to share in the next stage of the project where we work with patients, carers and staff to brainstorm solutions to improve the community rehabilitation service. We will show you the clips of the video to be shared, and you will be able to say yes or no to us sharing it. You will also have the opportunity to review your audio and/or interview transcript if you wish. Is this OK for you? Do you have any questions before we start?

<u>Participant characteristics (for Patient)</u>

1. What is your age? _____
2. What is your gender? Male/Female
3. What is your ethnicity? Chinese/Indian/Malay/Caucasian/Other, please specify:_____
4. What is your marital status? Single/Married/Divorced/Other, please specify:_____
5. What is your highest education qualification? PhD/Doctorate/Bachelor’s degree/Diploma/ Other, please specify:_____
6. Which of the following health care professionals were you meant to see as part of the community rehabilitation service? Doctor/Nurse/Physiotherapist/Occupational Therapist/Speech and Language Therapist/Social Worker/Psychologist/Therapy Assistant/Other, please specify:_____

<u>Participant characteristics (for Carer)</u>

7. I am the __________ of person who has accessed community rehabilitation services. Spouse/Parent/Sibling/Other, please specify:_____
8. What is your age? _____
9. What is your gender? Male/Female
10. What is your ethnicity? Chinese/Indian/Malay/Caucasian/Other, please specify:_____
11. What is your marital status? Single/Married/Divorced/Other, please specify:_____
12. What is your highest education qualification? PhD/Doctorate/Bachelor’s degree/Diploma/ Other, please specify:_____
13. What type of housing do you live in? HDB lift landing/HDB non-lift landing/ Private/Nursing home

<u>Background:</u>

14. Tell me about yourself and why you/person whom you care for were referred for community rehabilitation service.

<u>Reasons for non-usage of community rehabilitation service:</u>

15. What are the reasons for not using the community rehabilitation service?
    (Prompt: Tell me why you chose not to use the community rehabilitation service. Do you think it is a service that you need to use?)
16. What are some things that might encourage you to use the community rehabilitation service?
    (Prompt: Is there anything else we can add to the service that might encourage you to use it?)

[Interviewer to summarise reasons and solutions.]

Thank you so much for your time and responses.

Interview questions have been adapted from Jones F et al (2020) Using co-production to increase activity in acute stroke units: the CREATE mixed-methods study. *Health Serv Deliv Res* 8: 35 and van Deventer et al (2016) Improving childhood nutrition and wellness in South Africa: involving mothers/caregivers of malnourished or HIV positive children and health care workers as co-designers to enhance a local quality improvement intervention. *BMC Health Services Research* 16: 358.
