# Supplementary material for: Transforming Community‐Based Rehabilitation Services: A National Redesign Using Experience‐Based Co‐Design
Source: Health Expect. 2025 Jun 23;28(3):e70330. doi: 10.1111/hex.70330 (PMC12183464; doi:10.1111/hex.70330)
Supplement: Supplementary file 4 — Supporting Information 4. Surveys – Clinical and organisational surveys. [file HEX-28-e70330-s001.pdf]

Supplementary materials 4. Surveys – Clinical and organisational surveys

Clinical Practice of Centre-based Day Rehabilitation (19 questions; 25 minutes)

This survey aims to describe the clinical practices of centre-based day rehabilitation services provided in Singapore, specifically for clients with stroke, deconditioning/frailty and hip fracture. Adherence to international clinical practice guidelines has been shown to improve client outcomes and reduce unwarranted variations in practice. Understanding the current state of clinical practice will help in identifying the strengths as well as the areas for improvement. This survey is disseminated to rehabilitation clinicians working in the community, specifically at centres who are participating in the National One Rehabilitation framework. Your participation today is a critical first step in the work to elevate the allied health workforce as we move towards the '3 Beyonds'.

The survey contains two sections:

Section A: Clinician Profile (4 questions, 5 mins)
Section B: Clinical Practice – Centre-based Day Rehabilitation (15 questions, 20mins)

SECTION A: Clinician Profile

1. What is your health profession?
   - ☐ Physiotherapist (PT)
   - ☐ Occupational Therapist (OT)
   - ☐ Speech and Language Therapist (SLT)

2. How many years of experience do you have as a health professional? (Please only include the years where you are engaged in full-time, part-time or locum work. The work can be clinical care, or non-clinical care (inclusive of time spent in operations/AIC).
   - ☐ Less than 5 years
   - ☐ 5 to 10 years
   - ☐ More than 10 years

3. Are you aware of **international** clinical practice guidelines for the following conditions?

Conditions	Yes	No
Stroke		
Deconditioning/Frailty		
Hip Fracture		

4. How often do you practice or adhere to the international clinical practice guidelines for the following conditions?

**Conditions**	**Never**	**Rarely (<25% of clients)**	**Some times (25-50% of clients**	**Often (>50% of clients)**	**Always (all clients)**
Stroke					
Deconditioning/Frailty					
Hip Fracture					

Section B: Clinical Practice – Centre-based Day Rehabilitation (15 questions, 20mins)

In this section, a set of assessments and interventions are listed for stroke, deconditioning/frailty and hip fracture. For each condition, you will be asked to rate the frequency in which you conduct the assessment and intervention. There is no right or wrong answer. Please answer as honestly as you can.

1. Below is a list of possible **assessments for stroke** used by physiotherapists, occupational therapists, or speech and language therapists. Please rate how often you use the following assessments, as part of your usual clinical care amongst the clients with stroke. Outcomes to be collected as part of One-Rehab are not included in the table.

Assessments for Stroke	Never	Rarely (<25% of clients)	Some times (25-50% of clients	Often (>50% of clients)	Always (all clients)
Option to appear for PT/OT/ST					
Mini–Mental State Examination (MMSE)					
Montreal Cognitive Assessment (MoCA)					
Hospital Anxiety and Depression Scale (HADS)					
Patient Health Questionnaire-4 (PHQ-4)					
Options to appear for PT only					
Mobility Scale for Acute Stroke (MSAS)					
Motor Assessment Scale (MAS)					
Berg Balance Scale (BBS)					
Timed Up and Go (TUG)					
5 x Sit-to-Stand Test (5 x STS)					
Step Test					
Options to appear for OT only					
Action Research Arm Test (ARAT)					
Fugl Meyer Assessment-Upper Extremity (FMA-UE)					
Nine Hole Peg Test (9HPT)					
Option to appear for ST only					
Boston Diagnostic Aphasia Examination					
Comprehensive Aphasia Test					
Western Aphasia Battery (WAB)					
Psycholinguistics Assessments of Language Processing Abilities (PALPA)					
IDDSI Functional Diet Scale					

2. Are there any other assessments for stroke that you use in your clinical practice (this includes psychosocial assessments such as the Stroke Impact Scale? If none, please input “none”.

3. Below is a list of possible **interventions for stroke** provided by physiotherapists, occupational therapists, or speech and language therapists. Please rate how often you provide the following interventions as part of your usual clinical care for clients with stroke. If your centre does not have the equipment or facilities, please score NA, Not Applicable.

**Interventions for Stroke**	**Never**	**Rarely(<25% of clients)**	**Sometimes (25-50% of clients**	**Often (>50% of clients)**	**Always (all clients)**	**NA**
Options to appear for PT/OT/ST						
Education on neuroplasticity and rehabilitation journey						
Education on discharge planning and transfer of care						
Caregiver training						
Education on self-management						
Provision of information re: peer support to client and/or family (e.g., local stroke support group)						
Goal-setting						
Options to appear for PT/OT						
Strength training and/or progressive resistance training for arm/leg weakness						
Electrical stimulation for less than antigravity strength in leg						
Electrical stimulation for less than antigravity strength in arm						
Sensory-specific training for sensory loss						
Cardiorespiratory fitness training (e.g., walking, arm or leg cycling at moderate intensity)						
Repetitive task-specific practice of sitting, standing up, standing and/or walking						
Repetitive task-specific practice of upper limb activity						
Use of virtual reality training for standing and/or walking						
Use of force platform for standing balance training						
Use of electromechanical assisted device for standing and/or walking (e.g., body weight support, robotics)						
Lower limb orthoses for walking						
Use of virtual reality training for upper limb activity						
Use of electromechanical assisted device for upper limb activity (e.g., robotics)						
Upper limb orthoses/splints for contracture						

Constraint-induced movement therapy for upper limb activity in those with some active wrist and finger extension						
Mental practice with active motor training for upper limb activity in those with mild to moderate arm weakness						
Mirror therapy as adjunct to routine therapy for upper limb activity in those with mild to moderate arm weakness and/or neglect						
Recommendation of acupuncture for pain						
Recommendation of acupuncture for activities of daily living and spasticity						
Referral for non-invasive brain stimulation (transcranial direct current stimulation or repetitive transcranial magnetic stimulation)						
Referral to upstream providers for Botulinum Toxin A for spasticity						
Adjunct therapies for clients who have received Botulinum Toxin A (e.g., electrical stimulation)						
Routine use of stretch for spasticity and/or contracture						
Electrical stimulation for those at risk of shoulder subluxation						
Shoulder strapping for those at risk of shoulder subluxation						
Electrical stimulation for those with shoulder pain						
Shoulder strapping for those with shoulder pain						
Referral to upstream providers for shoulder injections and/or Botulinum Toxin A for those with shoulder pain						
Multifactorial interventions, such as, an individually prescribed exercise program and advice on safety for falls						
Outdoors mobility training						
Visuoperceptual rehabilitation (e.g., eye patching, mental practice, visual scanning training)						
Gesture training, strategy training and/or errorless learning for limb apraxia						
Referral to other providers for driving simulation						

Assessment and/or assistance with return to work for those who wish to return to work						
Options to appear for OT/ST						
Meta-cognitive strategy +/- cognitive training for executive function						
Cognitive rehabilitation (remediation pen & paper tasks) for neglect						
Cognitive rehabilitation (remediation leveraging on technology apps)						
Cognitive rehabilitation (remediation functional tasks training)						
Cognitive rehabilitation (compensatory strategies)						
Options to appear for OT only						
Provide targeted OT interventions in the areas of self-care and instrumental ADLs (e.g., grocery shopping, paying bills etc.)						
Provide targeted OT interventions in the areas of productivity, social participation, and leisure						
Options to appear for ST only						
Behavioural approaches for dysphagia (e.g., swallowing exercises, environmental modifications, safe swallowing advice, and appropriate dietary modifications)						
Recommendation of acupuncture for dysphagia						
Surface Electromyography (sEMG) for dysphagia						
Routine use of Neuromuscular Electrical Stimulation (NMES) for dysphagia						
Intensive aphasia therapy (at least 45 minutes of direct language therapy for five days a week) in the first few months after stroke for dysphasia						
Individually tailored interventions incorporating articulatory-kinematic and rate/rhythm approaches for speech apraxia (e.g., use of modelling and visual cueing, PROMPT therapy, self-administered computer programs that use multimodal sensory stimulation)						
Non-speech oromotor exercises						

Behavioural speech practice for dysarthria						
Assistance/education of clients to maintain good oral and dental hygiene, particularly in those with swallowing difficulties						
Education of staff and/or carers to maintain good oral and dental hygiene, particularly in those with swallowing difficulties						

4. Are there any other interventions for stroke that you use in your clinical practice (this includes psychosocial interventions such as mindfulness training)? If none, please input “none”.

5. Below is a list of possible **assessments for deconditioning/frailty** used by physiotherapists, occupational therapists, or speech and language therapists. Please rate how often you use the following assessments as part of your usual clinical care for clients with deconditioning/frailty. Outcomes to be collected as part of One-Rehab are not included in the table. If an assessment is not usually conducted by you, and is conducted by another allied healthcare professional in the team, please score NA, Not Applicable.

**Assessments for Deconditioning/Frailty**	**Never**	**Rarely (<25% of clients)**	**Sometimes (25-50% of clients**	**Often (>50% of clients)**	**Always (all clients)**	**NA**
Fried’s Frailty Phenotype						
Clinical Frailty Scale (CFS)						
FRAIL scale						
PRISMA-7						
Tilburg Fragility Index (TFI)						
Edmonton Frailty Scale (EFS)						
Study of Osteoporotic Fractures (SOF) index						
Rapid Geriatric Assessment (RGA)						
Comprehensive Geriatric Assessment (RGA)						
Integrated Care for Older People (ICOPE) instrument						
Kihon checklist						
Mini-Metal State Examination (MMSE)						
Montreal Cognitive Assessment (MoCA)						
Short Physical Performance Battery (SPPB)						
Timed-Up-and-Go (TUG)						
Grip strength						
IDDSI Functional Diet Scale						
Functional Oral Intake Scale						

6. Are there any other assessments for deconditioning/frailty that you use in your clinical practice (this includes psychosocial assessments such as the Hospital Anxiety and Depression Scale)? If none, please input “none”.

7. Below is a list of possible **interventions for deconditioning/frailty** provided by physiotherapists, occupational therapists, or speech and language therapists. Please rate how often you provide the following interventions as part of your usual clinical care for clients with deconditioning/frailty. (If an intervention is not usually conducted by you and is conducted by another allied healthcare professional in the team, please score NA, Not Applicable.)

**Interventions for Deconditioning/Frailty**	**Never**	**Rarely (<25% of clients)**	**Sometimes (25-50% of clients**	**Often (>50% of clients)**	**Always (all clients)**	**NA**
Referral to medical specialists (if required)						
Referral to allied health professionals (e.g., dietician) (if required)						
Education/Provision of information re: frailty care (can include physical activity counselling)						
Caregiver training						
Strengthening/resistance training (e.g., use of body weight, therabands, free weights, machines using weights and/or pneumatic resistance)						
Power training (e.g., jumping or other form of plyometrics, ballistic training, complex training)						
High Intensity Interval Training (HIIT)						
Functional activities (e.g., sit-to-stands, transfers, squats, stairs)						
Balance training (e.g., line walking, tandem foot standing, standing on one leg, heel-toe walking)						
Aerobic training (e.g., overground/treadmill walking, cycling)						
Flexibility training (e.g., stretching)						
Dual-task training (e.g., walking and citing serial numbers)						
Tai Chi						
Dance						
Functional cognitive training for mild cognitive impairment (MCI)						
Home modifications (e.g.,EASE recommendations)						
Prescription of equipment (e.g., walking aid, shower chair)						
Options to appear for ST only						

Behavioural approaches for dysphagia (e.g., swallowing exercises, environmental modifications, safe swallowing advice, and appropriate dietary modifications)						
Assistance/education of clients to maintain good oral and dental hygiene, particularly in those with swallowing difficulties						
Education of staff and/or carers to maintain good oral and dental hygiene, particularly in those with swallowing difficulties						

8. Are there any other interventions for deconditioning/frailty that you use in your clinical practice (this includes psychosocial interventions such as mindfulness training)? If none, please input "none".

9. Below is a list of possible **assessments for hip fracture** provided by physiotherapists, or occupational therapists. Please rate how often you use the following assessments as part of your usual clinical care for clients with hip fracture. Outcomes to be collected as part of One-Rehab are not included in the table. If an assessment is not usually conducted by you and is conducted by another allied healthcare professional in the team, please score NA, Not Applicable.

**Assessments for hip fracture**	**Never**	**Rarely (<25% of clients)**	**Sometimes (25-50% of clients**	**Often (>50% of clients)**	**Always (all clients)**	**NA**
Hip muscle strength						
Other lower leg muscle strength						
Short Physical Performance Battery (SPPB)						
Timed-Up-and-Go (TUG)						
Stair Climb Test						
Six Minute Walk Test (6MWT)						

10. Are there any other assessments for hip fracture that you use in your clinical practice (this includes psychosocial assessments such as the Hospital Anxiety and Depression Scale)? If none, please input "none".

11. Below is a list of possible **interventions for hip fracture** provided by physiotherapists, or occupational therapists. Please rate how often you provide the following interventions as part of your usual clinical care for clients with hip fracture. If an intervention is not usually conducted by you and is conducted by another allied healthcare professional in the team, please score NA, Not Applicable.

**Interventions for hip fracture**	**Never**	**Rarely (<25% of clients)**	**Sometimes (25-50% of clients**	**Often (>50% of clients)**	**Always (all clients)**	**NA**
Referral to medical specialists (if required)						

Referral to allied health professionals (e.g., dietician) (if required)						
Education/Provision of information re: hip fracture (can include falls prevention)						
Caregiver training						
Group circuit class therapy						
Strengthening/resistance training (e.g., use of body weight, therabands, free weights, machines using weights and/or pneumatic resistance)						
Power training (e.g., jumping or other form of plyometrics, ballistic training, complex training)						
High Intensity Interval Training (HIIT)						
Functional activities (e.g., sit-to-stands, transfers, squats, stairs)						
Balance training (e.g., line walking, tandem foot standing, standing on one leg, heel-toe walking)						
Aerobic training (e.g., overground/treadmill walking, cycling)						
Flexibility training (e.g., stretching)						
Dual-task training (e.g., walking and citing serial numbers)						
Tai Chi						
Dance						
Home modifications (e.g., rails)						
Prescription of equipment (e.g., walking aid, shower chair)						
Prescription of hip protector						
Prescription of aids for communication (e.g., eyeglasses, hearing aids)						

12. Are there any other interventions for hip fracture that you use in your clinical practice (this includes psychosocial interventions such as mindfulness training)? If none, please input "none".

The next three questions relate to skills-sharing, where practitioners perform clinical tasks that traditionally sit in the scope of practice of another profession. Skills-sharing is synonymous with trans-disciplinary practice. Skill-sharing cannot be used as substitution of a profession in the team as the expertise of the skill-sharing profession is required for management of complex clients.

13. Are there opportunities at your centre to share skills between professions to bring more holistic care to the clients? (e.g., an Occupational Therapist prescribing a gait aid)

- Yes, we practice skills sharing in our workplace in a well-defined manner.
- Yes, we practice skills sharing in our workplace randomly as deem fit by individual therapists.
- I am not aware but I am open to explore skill-sharing.
- I do not believe in skill-sharing.

14. In your opinion, which of the following clinical areas are feasible to skills-share between rehabilitation professionals in your setting?

**Skills sharing**	**Yes**	**No**
Administering a cognitive assessment tool (e.g. Montreal cognitive Assessment (MoCA))		
Administering cognitive rehabilitation strategies		
Administering screening to identify hearing impairments		
Screening for malnutrition		
Screening for mood problems		
Screening for carer strain using standardised questionnaires		
Providing education on nutrition		
Providing education on foot care		
Standing balance retraining		
Transfers training		
Mobility training		
Application of functional electrical stimulation for prevention of shoulder subluxation		
Application of functional electrical stimulation for strengthening of lower limb muscles		
Measurement of joint range of motion		
Prescription of Personal Mobility Devices (e.g., electric scooters)		

15. Are there any other clinical areas that are feasible to skills-share between rehabilitation professionals in your setting? If none, please input “none”.

Your input will help the workgroup in identifying the needs of the community rehabilitation workforce. Thank you for being part of Community Rehabilitation Transformation!

Survey for Rehabilitation Leaders and/or Champions

Clinical Organisation – Centre-based Day Rehabilitation (14 questions; 20 minutes)

In this survey, we are interested in understanding the current clinical organisation at your centre for clients with stroke, deconditioning/frailty, and hip fracture. This survey will help us understand the system level changes that are required for therapists to practise best care for best outcomes for their clients. **This survey is intended for the site champions only. Site champions please complete survey based on your centre's practice rather than individual practice.** There is no right or wrong answer. Please answer as honestly as you can.

1. What is the target number of clients per day, that therapists are expected to see for day rehabilitation, at your centre (e.g., 12 per day)

Profession	Target Number
Physiotherapist	
Occupational Therapist	
Speech and Language Therapist	

2. What is the estimated number of clients with stroke, deconditioning or hip fracture seen per month at the day rehabilitation at your centre?

Condition	Estimated number of clients per month
Stroke	
Deconditioning	
Hip Fracture	

3. Which of the following resources/services are available at your site?
   * Tailored information refers to the provision of customised information according to the specific characteristics of the individual to whom the information is being provided.

**Resources/Services**	**Yes**	**No**
Home-based rehabilitation (this can be by a single discipline, e.g., Physiotherapy only)		
Early supported discharge in collaboration with upstream providers		
Group circuit class therapy		
Telehealth for consultation and/or therapy sessions		
Provision of tailored* information on conditions (e.g., stroke, frailty), implications and recovery		
Provision of tailored* information on lifestyle (e.g., physical activity counselling)		
Provision of tailored* information on local community care arrangements		
Provision of tailored* information on community support groups		
Provision of tailored* information on stroke that is aphasia-friendly		
Provision of tailored* information on stroke that is age-appropriate for young clients		
Provision of tailored* discharge care plan to client		
Referral to a post-discharge self-management programme		
Provision of contact person at site that clients, or caregivers can contact upon discharge from site		
Caregiver training		

Return to work services/training, and/or vocational rehabilitation		
Return to driving training		
Follow-up home visit to client, or caregiver post-discharge		
Others, please specify:		

4. How does your site usually establish client-directed goals/individualised care plan? (Please select as many options as appropriate.)
   - ☐ Initial assessment with client by each discipline
   - ☐ Team meeting session with healthcare professionals to set/review goals
   - ☐ Team meeting session with client and healthcare professionals to set/review goals
   - ☐ Ad hoc - no consistent process used
   - ☐ Others, please specify: ________

5. Does your site use objective measures to review client-directed goals (e.g. Goal Attainment Scale)?
   - ☐ Yes, please specify: ________
   - ☐ No

6. How often does your site review client-directed goals/individualised care plan for clients, using *objective measures, on <u>active</u> rehabilitation? (Please select as many options as appropriate.)
   - ☐ At least once every month
   - ☐ At least once every 3 months
   - ☐ Every time there is a significant change in the client's function
   - ☐ Ad hoc - no consistent process used
   - ☐ Others, please specify: ________

   *objective measures refer to functional outcome measures (e.g. modified Barthel Index) or goal-setting measures (e.g. Goal Attainment Scale)

7. How does your site evaluate client-directed goals/individualised care plan for clients on <u>active</u> rehabilitation? (Please select as many options as appropriate.)
   - ☐ Reviewing change in Modified Barthel Index
   - ☐ Reviewing change using other outcome measures (e.g., gait speed)
   - ☐ Reviewing change in physical function (e.g., x1 moderate to x1 minimal assist with walking)
   - ☐ Interview session with the client
   - ☐ Ad hoc - no consistent process used
   - ☐ Others, please specify: ________

8. How does your site decide if a client is for active or maintenance rehabilitation? (Please select as many options as appropriate.)
   - ☐ If client have completed the maximum period of active rehabilitation
   - ☐ Reviewing change in Modified Barthel Index
   - ☐ Reviewing change using other outcome measures (e.g., gait speed)
   - ☐ Reviewing change in physical function (e.g., x1 moderate to x1 minimal assist with walking)
   - ☐ Interview session with the client
   - ☐ Dependent on therapist's judgement
   - ☐ Others, please specify: ________

9. How often does your site review client-directed goals/individualised care plan for clients, using *objective measures, on <u>maintenance</u> rehabilitation? (Please select as many options as appropriate.)
   - ☐ At least once every month
   - ☐ At least once every 3 months
   - ☐ Every time there is a significant change in the client's function
   - ☐ Ad hoc - no consistent process used
   - ☐ Others, please specify: ________

*objective measures refer to functional outcome measures (e.g. modified Barthel Index) or goal-setting measures (e.g. Goal Attainment Scale

10. How does your site evaluate client-directed goals/individualised care plan for clients on <u>maintenance</u> rehab? (Please select as many options as appropriate.)
    - ☐ Reviewing change in Modified Barthel Index
    - ☐ Reviewing change using other outcome measures
    - ☐ Reviewing change in physical function
    - ☐ Interview session with the client
    - ☐ Ad hoc - no consistent process used
    - ☐ Others, please specify: ________

11. In what capacity are the following medical and allied health professionals involved in the rehabilitation at your site? (NA refers to Not Applicable and is used if your site does not have the medical and allied health professional providing rehabilitation service.) (Please select as many options as appropriate for each row.)

**Medical and Allied Health Professionals**	**Full-time**	**Part-time**	**Locum**	**NA**
Rehabilitation physician				
Geriatrician				
Family medicine physician				
Neurologist				
General practitioner/visiting medical officers				
Medical resident				
Stroke liaison officer/stroke care coordinator				
Rehabilitation nurse				
Advance practice nurse				
Physiotherapist				
Occupational therapist				
Speech and language therapist				
Dietician				
Social worker				
Clinical psychologist				
Neuropsychologist				
Music Therapist				
Art Therapist				
Allied health assistant/therapy assistant				
Others, please specify:				

12. Which of the following protocols are available at your site to guide clinicians in ***identifying*** the specific rehabilitation complication for referral to polyclinic or tertiary institutions?

**Complications**	**Yes**	**No**
Protocol to identify spasticity of upper limb		
Protocol to identify spasticity of lower limb		
Protocol to identify post-stroke shoulder pain		
Protocol to identify post-stroke shoulder subluxation		
Protocol to identify incontinence		

Protocol to identify mood disturbances		
Protocol to identify falls risk		
Protocol to identify frailty		
Protocol to assess new onset of dizziness		
Protocol to refer for diabetic foot care		
Others, please specify:		

13. Which of the following protocols (e.g., organisational-specific clinical pathways or practice guidelines) are available at your site to guide clinicians in ***managing*** the specific rehabilitation complication?

Complications	Yes	No
Protocol to manage spasticity of upper limb		
Protocol to manage spasticity of lower limb		
Protocol to manage post-stroke shoulder pain		
Protocol to manage post-stroke shoulder subluxation		
Protocol to manage incontinence		
Protocol to manage mood disturbances		
Protocol to manage falls risk		
Protocol to manage frailty		
Others, please specify:		

The next question relates to skills-sharing, where practitioners perform clinical tasks that traditionally sit in the scope of practice of another profession. Skills-sharing is synonymous with trans-disciplinary practice. Skill-sharing cannot be used as substitution of a profession in the team as the expertise of the skill-sharing profession is required for management of complex clients

14. a) Does your site practice skills-sharing?
    - ☐ Yes, please proceed to (b).
    - ☐ No, please proceed to Q15.

    b) Which of the following processes are available at your site to enable therapists to skills-share?

**Skills sharing**	**Yes**	**No**
List of tasks that can be skill-shared across professional boundaries		
Competency checklists to standardise how tasks are skill-shared		
Governance processes to support clinicians (e.g., internal audits)		
Structured training and competence assessment for professional skill-sharing and delegation practice		

Competency assessments for professional skill-sharing and delegation practice		
System level processes for sustainability of skills-sharing practice (e.g., part of orientation training for new staff, annual competency check)		
Others, please specify:		

The results of the survey will be disseminated to you in Phase II of this project. We thank you for your time and for your enthusiasm for transforming community rehabilitation in Singapore!
