# Supplementary material for: Transforming Community‐Based Rehabilitation Services: A National Redesign Using Experience‐Based Co‐Design
Source: Health Expect. 2025 Jun 23;28(3):e70330. doi: 10.1111/hex.70330 (PMC12183464; doi:10.1111/hex.70330)
Supplement: Supplementary file 5 — Supporting Information 5. Case note reviews – Template. [file HEX-28-e70330-s007.pdf]

Supplementary material 5. Case note reviews – Template

CASE NOTES REVIEW FOR CENTRE-BASED CARE/DAY REHAB [STROKE]

1. REVIEWER INFORMATION
1.1 Centre name
1.2 Reviewer name
1.3 Reviewer email
1.4 Reviewer discipline PT / OT / SLT / Others

2. CLIENT DEMOGRAPHICS
CLIENT DETAILS
2.1 Client record ID
2.2 Client IC (last 4 digits)
2.3 Date of birth
2.4 Age (Auto-populated from 2.3)
2.5 Gender M / F
2.6 Race Chinese / Malay / Indian / Eurasian / Others

3. ADMISSION INFORMATION
ADMISSION DETAILS
3.1 Date of condition onset
3.2 Date of first session at centre
3.3 Where was the client referred from? Acute hospital / Community hospital / Specialist Outpatient Clinic / Polyclinic / General Practitioner Clinic / Others / Unknown

FUNCTIONAL STATUS ON ADMISSION
3.4 Admission Modified Barthel Index (MBI) [Actual score] / "." if unknown or incomplete
3.5 Admission Modified Rankin Scale (MRS) [Actual score]. (Determined by reviewer using simplified mRS algorithm)

IMPAIRMENTS REQUIRING REHABILITATION
3.6 Does client have motor deficits? Yes / No
3.7 Does client have arm deficits? Yes / No
3.8 Does client have sensory deficits? Yes / No
3.9 Does client have cognitive deficits? Yes / No
3.10 Does client have visual deficits? Yes / No
3.11 Does client have perceptual deficits? Yes / No
3.12 Does client have speech and communication deficits? Yes / No
3.13 Does client have swallowing deficits? Yes / No

ACTIVITY LIMITATIONS REQUIRING REHABILITATION
3.14 Does client require rehabilitation for transfer? Yes / No
3.15 Does client require rehabilitation for standing up? Yes / No
3.16 Does client require rehabilitation for walking? Yes / No
3.17 Does client require rehabilitation for washing/showering self? Yes / No
3.18 Does client require rehabilitation for toileting? Yes / No
3.19 Does client require rehabilitation for dressing? Yes / No
3.20 Does client require rehabilitation for functional use of arm? Yes / No
3.21 Does client require rehabilitation for communication/speech and language? Yes / No
3.22 Does client require rehabilitation for feeding? Yes / No
3.23 Does client require rehabilitation for swallowing? Yes / No

PARTICIPATION RESTRICTIONS REQUIRING REHABILITATION
3.24 Does client require rehabilitation for return to work? Yes / No / Not assessed
3.25 Does client require rehabilitation for return to driving? Yes / No / Not assessed
3.26 Does client require rehabilitation for Instrumental Activities of Daily Living (ADLs)? Yes / No / Not assessed

4. ASSESSMENT AND MANAGEMENT

ALLIED HEALTH SEEN
4.1 Was client seen by the following allied health staff while undergoing rehabilitation at the centre?
Physiotherapist Yes / No / Client declined
Occupational therapist Yes / No / Client declined
Speech and language therapist Yes / No / Client declined
Social worker Yes / No / Client declined
Dietician Yes / No / Client declined
Psychologist Yes / No / Client declined

ASSESSMENT
4.2 Which of the allied health professional(s) did the following assessments during client's rehabilitation at the centre?
(Select from options: PT / OT / SLT / PT and OT / OT and SLT / PT and SLT / PT, OT and SLT / Not Done / Others, please specify:__)

Action Research Arm Test (ARAT)
AusTOMS for Speech Pathology
Berg Balance Scale (BBS)
Boston Diagnostic Aphasia Examination
Comprehensive Aphasia Test
EQ5D-5L
Frenchay Activities Index (FAI)
Fugl Meyer Assessment-Upper Extremity (FMA-UE)
Functional Oral Intake Scale (FOIS)
Gait speed
Hospital Anxiety and Depression Scale (HADS)
IDDSI Functional Diet Scale
Joint range of motion
Mini–Mental State Examination (MMSE)
Mobility Scale for Acute Stroke (MSAS)
Modified Barthel Index
Montreal Cognitive Assessment (MoCA)
Motor Assessment Scale (MAS)
Muscle strength - Grip strength
Muscle strength – Manual Muscle Testing
Nine Hole Peg Test (9HPT)
Patient Health Questionnaire-4 (PHQ-4)
Psycholinguistics Assessments of Language Processing Abilities (PALPA)
Screening for hearing impairments
Screening for malnutrition
Screening for mood problems
Screening for carer strain using standardized questionnaires
Step Test
Timed Up and Go (TUG)
Western Aphasia Battery (WAB)
5 x Sit-to-Stand Test (5 x STS)

4.3 If there are other assessment(s) done, please specify assessment(s) and allied health professional(s) conducting the assessment(s) at the centre.

MANAGEMENT
4.4 Which of the allied health professional(s) did the following treatments during client's rehabilitation at the centre?
(Select from options: PT / OT / SLT / PT and OT / OT and SLT / PT and SLT / PT, OT and SLT / Not Done / Others, please specify:__)

Upper limb
Constraint-induced movement therapy for upper limb activity in those with some active wrist and finger extension

Electrical stimulation for less than antigravity strength in arm
Electromechanical assisted device for upper limb activity (e.g., robotics)
Mental practice with active motor training for upper limb activity in those with mild to moderate arm weakness
Mirror therapy as adjunct to routine therapy for upper limb activity in those with mild to moderate arm weakness and/or neglect
Orthotic devices (upper limb) (e.g., splints) for contracture
Progressive strength training and/or resistance training for arm weakness
Repetitive task-specific practice of upper limb activity
Sensory-specific training for sensory loss
Spasticity management – referral to upstream providers for Botulinum Toxin A
Spasticity management – adjunct therapies for clients who have received Botulinum Toxin A (e.g., electrical stimulation)
Stretching for spasticity and/or contracture
Shoulder pain – referral to upstream providers for shoulder injections and/or Botulinum Toxin A
Shoulder pain – electrical stimulation
Shoulder pain – shoulder strapping
Shoulder subluxation – electrical stimulation for those at risk
Shoulder subluxation – shoulder strapping for those at risk
Virtual reality training for upper limb activity

<u>Lower limb</u>
Cardiorespiratory fitness training (e.g., walking, arm or leg cycling at moderate intensity)
Electrical stimulation for less than antigravity strength in leg
Electromechanical assisted device for standing and/or walking (e.g., body weight support, robotics)
Group circuit class therapy
Orthotic devices (lower limb) for walking
Outdoors mobility training
Progressive strength training and/or resistance training for leg weakness
Repetitive task-specific practice of bed mobility
Repetitive task-specific practice of transfers
Repetitive task-specific practice of sitting
Repetitive task-specific practice of standing up
Repetitive task-specific practice of standing
Repetitive task-specific practice of walking
Sensory-specific training for sensory loss
Spasticity management – referral to upstream providers for Botulinum Toxin A
Spasticity management – adjunct therapies for clients who have received Botulinum Toxin A (e.g., electrical stimulation)
Stretching for spasticity and/or contracture
Stairs practice
Virtual reality training for standing and/or walking

<u>Cognition</u>
Cognitive rehabilitation (remediation pen & paper tasks) for neglect
Cognitive rehabilitation (remediation leveraging on technology apps)
Cognitive rehabilitation (remediation functional tasks training)
Cognitive rehabilitation (compensatory strategies)
Gesture training, strategy training and/or errorless learning for limb apraxia
Meta-cognitive strategy +/- cognitive training for executive function
Visuoperceptual rehabilitation (e.g., eye patching, mental practice, visual scanning training)
Dual-tasking with cognitive task (e.g., standing and doing maths calculation, walking and talking)

<u>Speech</u>
Assistance/education of clients to maintain good oral and dental hygiene, particularly in those with swallowing difficulties
Education of staff and/or carers to maintain good oral and dental hygiene, particularly in those with swallowing difficulties
Dysarthria management – behavioural speech practice for dysarthria

Dysphagia management – behavioural approaches (e.g., swallowing exercises, environmental modifications, safe swallowing advice, and appropriate dietary modifications)
Dysphagia management – recommendation of acupuncture
Dysphagia management – surface Electromyography (sEMG)
Dysphagia management – Neuromuscular Electrical Stimulation (NMES)
Dysphasia management – intensive aphasia therapy (at least 45 minutes of direct language therapy for five days a week) in the first few months after stroke
Non-speech oromotor exercises
Speech apraxia – individually tailored interventions incorporating articulatory-kinematic and rate/rhythm approaches (e.g., use of modelling and visual cueing, PROMPT therapy, self-administered computer programs that use multimodal sensory stimulation)

Others
Activities of daily living - self-care (e.g., showering, dressing, toileting)
Activities of daily living - instrumental (e.g., grocery shopping, paying bills etc.)
Acupuncture – recommendation for pain
Acupuncture – recommendation for activities of daily living and spasticity
Driving – referral to other providers for driving simulation
Falls management – multifactorial interventions (e.g., individually prescribed exercise program and safety advice)
Home modifications (e.g., EASE recommendations)
Non-invasive brain stimulation (transcranial direct current stimulation or repetitive transcranial magnetic stimulation) – referral to other providers
Prescription of equipment (e.g., walking aid, shower chair)
Prescription of personal mobility devices (e.g., electric scooters)
Productivity, social participation, and leisure – targeted OT interventions
Providing education on nutrition
Providing education on foot care
Return to work – assessment and/or assistance for those who wish to return to work

4.5 Please list type of instrumental ADLs done (if any):

4.6 If there are other treatment(s) done, please specify treatment(s) and allied health professional(s) conducting the treatment(s) at the centre.

COMPLICATIONS
4.7 Did the client have any of the following complications while undergoing rehabilitation at the centre?
Contracture Yes / No
Spasticity Yes / No
Altered mood (e.g., anxiety, depression) Yes / No
Shoulder subluxation Yes / No
Shoulder pain Yes / No
Pain in other parts of body (other than shoulder) Yes / No
Fatigue Yes / No

COMMUNICATION AND SUPPORT FOR CLIENT AND FAMILY/CARER
4.8 Did the team meet with the client to discuss management? Yes / No / No but met with family/carer
4.9 Were goals set with input from the team and client? Yes / No / No but met with family/carer
4.10 Was the client/carer provided with any of the following education/information prior to discharge?
Provision of tailored* information on condition, implications and recovery Yes / No
Provision of tailored* information on lifestyle (e.g., physical activity counselling) Yes / No
Provision of tailored* information on self-management Yes / No
Provision of tailored* information on local community care arrangements Yes / No
Provision of tailored* information on community support groups Yes / No
Provision of tailored* information on stroke that is aphasia-friendly Yes / No
Provision of tailored* information on stroke that is age-appropriate for young clients Yes / No
Provision of tailored* discharge care plan to client Yes / No
Provision of contact person at site that clients, or caregivers can contact upon discharge from site Yes / No

Others, please specify:

*Tailored information refers to the provision of customised information according to the specific characteristics of the individual to whom the information is being provided.

4.11 Does the client have a carer? Yes / No / Not required
4.12 If the client has a carer, did the carer receive caregiver training? Yes / No / NA
4.13 If the client has a carer, did the carer receive a support needs assessment (e.g., physical, emotional, and social)? Yes / No / NA
4.14 Was the carer provided with information about carer peer support resources prior to client's discharge? Yes / No / NA

5. DISCHARGE
DISCHARGE DETAILS
5.1 Date of last session at centre

FUNCTIONAL STATUS ON DISCHARGE
5.2 Discharge Modified Barthel Index [Actual score] / "." if unknown or incomplete
5.3 Discharge Modified Rankin Scale [Actual score] (Determined by reviewer using simplified mRS algorithm)

SUPPORT ON DISCHARGE
5.4 What is the level of support required on discharge?
Lives alone (no formal supports) / Lives alone (formal supports) / Lives with others (no formal supports) / Lives with others (formal supports)
5.5 Is this the same level of support as before the stroke?
Yes / No / Unknown
5.6 Is there evidence that a care plan outlining post discharge care in the community was developed with the team and the client (or family alone if client has severe aphasia or cognitive impairments)?
Yes, with client and/or family/carer / No / NA (e.g., transfer back to hospital for acute event)
5.7 Is there evidence that the general practitioner and or community providers were provided with a copy of the discharge summary? Yes / No / NA (e.g., transfer back to hospital for acute event)

FURTHER REHABILITATION AND COMMUNITY RE-INTEGRATION
5.8 Was the client/carer provided with any of the following service post-discharge?
Follow-up telephone call to client, or carer post-discharge Yes / No / NA
Follow-up home visit to client, or carer post-discharge Yes / No / NA
Referral to a post-discharge self-management programme Yes / No / NA
Referral to return to work services/training, and/or vocational rehabilitation Yes / No / NA
Referral to return to driving training Yes / No / NA
Referral to maintenance programme at day rehab Yes / No / NA
Referral to daycare programme Yes / No / NA
Referral to Tier 3 Yes / No / NA
Others, please specify:

CASE NOTES REVIEW FOR CENTRE-BASED CARE/DAY REHAB <u>[DECONDITIONING]</u>

1. REVIEWER INFORMATION
1.1 Centre name
1.2 Reviewer name
1.3 Reviewer email
1.4 Reviewer discipline PT / OT / SLT / Others

2. CLIENT DEMOGRAPHICS
CLIENT DETAILS
2.1 Client record ID
2.2 Client IC (last 4 digits)
2.3 Date of birth
2.4 Age (Auto-populated from 2.3)
2.5 Gender M / F
2.6 Race Chinese / Malay / Indian / Eurasian / Others

3. ADMISSION INFORMATION
ADMISSION DETAILS
3.1 Date of condition onset
3.2 Date of first session at centre
3.3 Where was the client referred from? Acute hospital / Community hospital / Specialist Outpatient Clinic / Polyclinic / General Practitioner Clinic / Others / Unknown

FUNCTIONAL STATUS ON ADMISSION
3.4 Admission Modified Barthel Index [Actual score] / "." if unknown or incomplete
3.5 Any falls in the last 6 months? Yes / No

IMPAIRMENTS REQUIRING REHABILITATION
3.6 Does client have arm deficits? Yes / No
3.7 Does client have leg deficits? Yes / No
3.8 Does client have cognitive deficits? Yes / No
3.9 Does client have visual deficits? Yes / No
3.10 Does client have balance deficits? Yes / No
3.11 Does client have speech and communication deficits? Yes / No
3.12 Does client have swallowing deficits? Yes / No

ACTIVITY LIMITATIONS REQUIRING REHABILITATION
3.13 Does client require rehabilitation for transfer? Yes / No
3.14 Does client require rehabilitation for standing up? Yes / No
3.15 Does client require rehabilitation for walking? Yes / No
3.16 Does client require rehabilitation for washing/showering self? Yes / No
3.17 Does client require rehabilitation for toileting? Yes / No
3.18 Does client require rehabilitation for dressing? Yes / No
3.19 Does client require rehabilitation for communication/speech and language? Yes / No
3.20 Does client require rehabilitation for feeding? Yes / No
3.21 Does client require rehabilitation for swallowing? Yes / No

PARTICIPATION RESTRICTIONS REQUIRING REHABILITATION
3.22 Does client require rehabilitation for return to work? Yes / No / Not assessed
3.23 Does client require rehabilitation for return to driving? Yes / No / Not assessed
3.24 Does client require rehabilitation for Instrumental Activities of Daily Living (ADLs)? Yes / No / Not assessed

4. ASSESSMENT AND MANAGEMENT
ALLIED HEALTH SEEN
4.1 Was client seen by the following allied health staff while undergoing rehabilitation at the centre?
Physiotherapist Yes / No / Client declined
Occupational therapist Yes / No / Client declined
Speech and language therapist Yes / No / Client declined
Social worker Yes / No / Client declined

Dietician Yes / No / Client declined
Psychologist Yes / No / Client declined

ASSESSMENT
4.2 Which of the allied health professional(s) did the following assessments during client's rehabilitation at the centre?
(Select from options: PT / OT / SLT / PT and OT / OT and SLT / PT and SLT / PT, OT and SLT / Not Done / Others, please specify:__)

Berg Balance Scale (BBS)
Clinical Frailty Scale (CFS)
Comprehensive Geriatric Assessment (RGA)
Edmonton Frailty Scale (EFS)
FRAIL scale
Fried's Frailty Phenotype
Functional Oral Intake Scale
Gait speed
IDDSI Functional Diet Scale
Integrated Care for Older People (ICOPE) instrument
Joint range of motion
Kihon checklist
Mini-Metal State Examination (MMSE)
Montreal Cognitive Assessment (MoCA)
Muscle strength - Grip strength
Muscle strength – Manual Muscle Testing
PRISMA-7
Rapid Geriatric Assessment (RGA)
Screening for hearing impairments
Screening for malnutrition
Screening for mood problems
Screening for carer strain using standardized questionnaires
Short Physical Performance Battery (SPPB)
Study of Osteoporotic Fractures (SOF) index
Tilburg Fragility Index (TFI)
Timed-Up-and-Go (TUG)
5 x Sit-to-Stand Test (5 x STS)

4.3 If there are other assessment(s) done, please specify assessment(s) and allied health professional(s) conducting the assessment(s) at the centre.

MANAGEMENT
4.4 Which of the allied health professional(s) did the following treatments during client's rehabilitation at the centre?
(Select from options: PT / OT / SLT / PT and OT / OT and SLT / PT and SLT / PT, OT and SLT / Not Done / Others, please specify:__)
Activities of daily living - self-care (e.g., showering, dressing, toileting)
Activities of daily living - instrumental (e.g., grocery shopping, paying bills etc.)
Aerobic training (e.g., overground/treadmill walking, cycling)
Balance training (e.g., line walking, tandem foot standing, standing on one leg, heel-toe walking)
Dance
Dual-task training (e.g., walking and citing serial numbers)
Flexibility training (e.g., stretching)
Functional cognitive training for mild cognitive impairment (MCI)
Functional activities (e.g., sit-to-stands, transfers, squats, stairs)
Group circuit class therapy
High Intensity Interval Training (HIIT)
Home modifications (e.g., EASE recommendations)
Outdoors mobility training
Power training (e.g., jumping or other form of plyometrics, ballistic training, complex training)

Providing education on nutrition
Providing education on foot care
Prescription of equipment (e.g., walking aid, shower chair)
Prescription of personal mobility devices (e.g., electric scooters)
Referral to medical specialists (if required)
Referral to allied health professionals (e.g., dietician) (if required)
Strengthening/resistance training (e.g., use of body weight, therabands, free weights, machines using weights and/or pneumatic resistance) [Any form of individualised, progressive program w resistance training as per Dent 2017, Dent 2019]
Tai Chi

Speech
Behavioural approaches for dysphagia (e.g., swallowing exercises, environmental modifications, safe swallowing advice, and appropriate dietary modifications)
Assistance/education of clients to maintain good oral and dental hygiene, particularly in those with swallowing difficulties
Education of staff and/or carers to maintain good oral and dental hygiene, particularly in those with swallowing difficulties

4.5 Please list type of instrumental ADLs done (if any):

4.6 If there are other treatment(s) done, please specify treatment(s) and allied health professional(s) conducting the treatment(s) at the centre.

COMPLICATIONS
4.7 Did the client have any of the following complications while undergoing rehabilitation at the centre?
Contracture Yes / No
Altered mood (e.g., anxiety, depression) Yes / No
Pain Yes / No
Fatigue Yes / No

COMMUNICATION AND SUPPORT FOR CLIENT AND FAMILY/CARER
4.8 Did the team meet with the client to discuss management? Yes / No / No but met with family/carer
4.9 Were goals set with input from the team and client? Yes / No / No but met with family/carer
4.10 Was the client/carer provided with any of the following education/information prior to discharge?
Provision of tailored* information on condition, implications and recovery Yes / No
Provision of tailored* information on lifestyle (e.g., physical activity counselling) Yes / No
Provision of tailored* information on self-management Yes / No
Provision of tailored* information on falls prevention and other related topics (e.g., frailty care) Yes / No
Provision of tailored* information on local community care arrangements Yes / No
Provision of tailored* information on community support groups Yes / No
Provision of tailored* discharge care plan to client Yes / No
Provision of contact person at site that clients, or caregivers can contact upon discharge from site Yes / No
Others, please specify:

*Tailored information refers to the provision of customised information according to the specific characteristics of the individual to whom the information is being provided.

4.11 Does the client have a carer? Yes / No / Not required
4.12 If the client has a carer, did the carer receive caregiver training? Yes / No / NA
4.13 If the client has a carer, did the carer receive a support needs assessment (e.g., physical, emotional, and social)? Yes / No / NA
4.14 Was the carer provided with information about carer peer support resources prior to client's discharge? Yes / No / NA

5. DISCHARGE
DISCHARGE DETAILS

5.1 Date of last session at centre

FUNCTIONAL STATUS ON DISCHARGE
5.2 Discharge Modified Barthel Index [Actual score] / "." if unknown or incomplete

SUPPORT ON DISCHARGE
5.3 What is the level of support required on discharge?
Lives alone (no formal supports) / Lives alone (formal supports) / Lives with others (no formal supports) / Lives with others (formal supports)
5.4 Is this the same level of support as before the current deconditioning episode?
Yes / No / Unknown
5.5 Is there evidence that a care plan outlining post discharge care in the community was developed with the team and the client (or family alone if client has severe cognitive impairments)?
Yes, with client and/or family/carer / No / NA (e.g., transfer back to hospital for acute event)
5.6 Is there evidence that the general practitioner and or community providers were provided with a copy of the discharge summary? Yes / No / NA (e.g., transfer back to hospital for acute event)

FURTHER REHABILITATION AND COMMUNITY RE-INTEGRATION
5.7 Was the client/carer provided with any of the following service post-discharge?
Follow-up telephone call to client, or carer post-discharge Yes / No / NA
Follow-up home visit to client, or carer post-discharge Yes / No / NA
Referral to a post-discharge community-based programme Yes / No / NA
Referral to return to work services/training, and/or vocational rehabilitation Yes / No / NA
Referral to return to driving training Yes / No / NA
Referral to maintenance programme at day rehab Yes / No / NA
Referral to daycare programme Yes / No / NA
Referral to Tier 3 Yes / No / NA
Others, please specify:

CASE NOTES REVIEW FOR CENTRE-BASED CARE/DAY REHAB [HIP FRACTURE]

1. REVIEWER INFORMATION
1.1 Centre name
1.2 Reviewer name
1.3 Reviewer email
1.4 Reviewer discipline PT / OT / SLT / Others

2. CLIENT DEMOGRAPHICS
CLIENT DETAILS
2.1 Client record ID
2.2 Client IC (last 4 digits)
2.3 Date of birth
2.4 Age (Auto-populated from 2.3)
2.5 Gender M / F
2.6 Race Chinese / Malay / Indian / Eurasian / Others

3. ADMISSION INFORMATION
ADMISSION DETAILS
3.1 Date of condition onset
3.2 Date of first session at centre
3.3 Where was the client referred from? Acute hospital / Community hospital / Specialist Outpatient Clinic / Polyclinic / General Practitioner Clinic / Others / Unknown

FUNCTIONAL STATUS ON ADMISSION
3.4 Admission Modified Barthel Index [Actual score] / "." if unknown or incomplete
3.5 Any falls in the last 6 months? Yes / No
3.6 Weight bearing status on admission? Not stated / FWB / PWB / Toe touch weight bear / NWB / Others

IMPAIRMENTS REQUIRING REHABILITATION
3.7 Does client have arm deficits? Yes / No
3.8 Does client have leg deficits? Yes / No
3.9 Does client have cognitive deficits? Yes / No
3.10 Does client have visual deficits? Yes / No
3.11 Does client have balance deficits? Yes / No
3.12 Does client have dizziness/vestibular issues? Yes / No

ACTIVITY LIMITATIONS REQUIRING REHABILITATION
3.13 Does client require rehabilitation for transfer? Yes / No
3.14 Does client require rehabilitation for standing up? Yes / No
3.15 Does client require rehabilitation for walking? Yes / No
3.16 Does client require rehabilitation for washing/showering self? Yes / No
3.17 Does client require rehabilitation for toileting? Yes / No
3.18 Does client require rehabilitation for dressing? Yes / No
3.19 Does client require rehabilitation for communication/speech and language? Yes / No
3.20 Does client require rehabilitation for feeding? Yes / No
3.21 Does client require rehabilitation for swallowing? Yes / No

PARTICIPATION RESTRICTIONS REQUIRING REHABILITATION
3.22 Does client require rehabilitation for return to work? Yes / No / Not assessed
3.23 Does client require rehabilitation for return to driving? Yes / No / Not assessed
3.24 Does client require rehabilitation for Instrumental Activities of Daily Living (ADLs)? Yes / No / Not assessed

4. ASSESSMENT AND MANAGEMENT
ALLIED HEALTH SEEN
4.1 Was client seen by the following allied health staff while undergoing rehabilitation at the centre?
Physiotherapist Yes / No / Client declined
Occupational therapist Yes / No / Client declined
Social worker Yes / No / Client declined
Psychologist Yes / No / Client declined

ASSESSMENT
4.2 Which of the allied health professional(s) did the following assessments during client's rehabilitation at the centre?
(Select from options: PT / OT / SLT / PT and OT / OT and SLT / PT and SLT / PT, OT and SLT / Not Done / Others, please specify:__)

Gait speed
Hip muscle strength
Hip joint range of motion
Montreal Cognitive Assessment (MoCA)
Other leg muscle strength
Other joint range of motion
Pain score
Screening for hearing impairments
Screening for malnutrition
Screening for mood problems
Screening for carer strain using standardized questionnaires
Six Minute Walk Test (6MWT)
Short Physical Performance Battery (SPPB)
Stair Climb Test
Timed-Up-and-Go (TUG)

4.3 If there are other assessment(s) done, please specify assessment(s) and allied health professional(s) conducting the assessment(s) at the centre.

MANAGEMENT
4.4 Which of the allied health professional(s) did the following treatments during client's rehabilitation at the centre?
(Select from options: PT / OT / SLT / PT and OT / OT and SLT / PT and SLT / PT, OT and SLT / Not Done / Others, please specify:__)

Activities of daily living - self-care (e.g., showering, dressing, toileting)
Activities of daily living - instrumental (e.g., grocery shopping, paying bills etc.)
Aerobic training (e.g., overground/treadmill walking, cycling)
Balance training (e.g., line walking, tandem foot standing, standing on one leg, heel-toe walking)
Cognitive rehabilitation strategies
Dance
Dual-task training (e.g., walking and citing serial numbers)
Flexibility training (e.g., stretching)
Functional activities (e.g., sit-to-stands, transfers, squats, stairs)
Group circuit class therapy
High Intensity Interval Training (HIIT)
Home modifications (e.g., EASE recommendations)
Outdoors mobility training
Power training (e.g., jumping or other form of plyometrics, ballistic training, complex training)
Prescription of hip protector
Prescription of aids for communication (e.g., eyeglasses, hearing aids)
Prescription of equipment (e.g., walking aid, shower chair)
Prescription of personal mobility devices (e.g., electric scooters)
Providing education on nutrition
Providing education on foot care
Referral to medical specialists (if required)
Referral to allied health professionals (e.g., dietician) (if required)
Strengthening/resistance training (e.g., use of body weight, therabands, free weights, machines using weights and/or pneumatic resistance) [Any form of weight bearing exercise as per ANZ 2014]
Tai Chi

4.5 Please list type of instrumental ADLs done (if any):

4.6 If there are other treatment(s) done, please specify treatment(s) and allied health professional(s) conducting the treatment(s) at the centre.

COMPLICATIONS
4.7 Did the client have any of the following complications while undergoing rehabilitation at the centre? Contracture Yes / No
Altered mood (e.g., anxiety, depression) Yes / No
Pain Yes / No
Fatigue Yes / No

COMMUNICATION AND SUPPORT FOR CLIENT AND FAMILY/CARER
4.8 Did the team meet with the client to discuss management? Yes / No / No but met with family/carer
4.9 Were goals set with input from the team and client? Yes / No / No but met with family/carer
4.10 Was the client/carer provided with any of the following education/information prior to discharge?
Provision of tailored* information on condition, implications and recovery Yes / No
Provision of tailored* information on lifestyle (e.g., physical activity counselling) Yes / No
Provision of tailored* information on self-management Yes / No
Provision of tailored* information on falls prevention and other related topics (e.g., frailty care) Yes / No
Provision of tailored* information on local community care arrangements Yes / No
Provision of tailored* information on community support groups Yes / No
Provision of tailored* discharge care plan to client Yes / No
Provision of contact person at site that clients, or caregivers can contact upon discharge from site Yes / No
Others, please specify:

*Tailored information refers to the provision of customised information according to the specific characteristics of the individual to whom the information is being provided.

4.11 Does the client have a carer? Yes / No / Not required
4.12 If the client has a carer, did the carer receive caregiver training? Yes / No / NA
4.13 If the client has a carer, did the carer receive a support needs assessment (e.g., physical, emotional, and social)? Yes / No / NA
4.14 Was the carer provided with information about carer peer support resources prior to client's discharge? Yes / No / NA

5. DISCHARGE
DISCHARGE DETAILS
5.1 Date of last session at centre

FUNCTIONAL STATUS ON DISCHARGE
5.2 Discharge Modified Barthel Index [Actual score] / "." if unknown or incomplete

SUPPORT ON DISCHARGE
5.3 What is the level of support required on discharge?
Lives alone (no formal supports) / Lives alone (formal supports) / Lives with others (no formal supports) / Lives with others (formal supports)
5.4 Is this the same level of support as before the hip fracture?
Yes / No / Unknown
5.5 Is there evidence that a care plan outlining post discharge care in the community was developed with the team and the client (or family alone if client has severe cognitive impairments)?
Yes, with client and/or family/carer / No / NA (e.g., transfer back to hospital for acute event)
5.6 Is there evidence that the general practitioner and or community providers were provided with a copy of the discharge summary? Yes / No / NA (e.g., transfer back to hospital for acute event)

FURTHER REHABILITATION AND COMMUNITY RE-INTEGRATION
5.7 Was the client/carer provided with any of the following service post-discharge?
Follow-up telephone call to client, or carer post-discharge Yes / No / NA
Follow-up home visit to client, or carer post-discharge Yes / No / NA
Referral to a post-discharge community-based programme Yes / No / NA

CASE NOTES REVIEW FOR CENTRE-BASED CARE/DAY REHAB [HIP FRACTURE]

Referral to return to work services/training, and/or vocational rehabilitation Yes / No / NA
Referral to return to driving training Yes / No / NA
Referral to maintenance programme at day rehab Yes / No / NA
Referral to daycare programme Yes / No / NA
Referral to Tier 3 Yes / No / NA
Others, please specify:
