# Supplementary material for: Transforming Community‐Based Rehabilitation Services: A National Redesign Using Experience‐Based Co‐Design
Source: Health Expect. 2025 Jun 23;28(3):e70330. doi: 10.1111/hex.70330 (PMC12183464; doi:10.1111/hex.70330)
Supplement: Supplementary file 6 — Supporting Information 6. Details of the feedback events and joint workshop. [file HEX-28-e70330-s002.pdf]

Supplementary materials 6. Details of the feedback events and joint workshop

**Event/workshop**	**Agenda and format of event/workshop, and accompanying materials**
Stage 4 of EBCD: Feedback event with staff	<u>Agenda</u><br>a) Overview and recap of results from interviews, time motion studies, surveys and case note reviews (+ Q&A)<br>b) Rating exercise 1: To select the top five priority statements from 15 (via Google form)<br>c) Rating exercise 2: To rate each of the 15 statements in terms of importance on a scale of 1 to 10 (via Google form)<br>d) Rating exercise 3: To identify the most important 10 priority statements and rank them in terms of importance and feasibility using a graph theory-based voting system (via Google form)<br>e) Issue feedback form on event<br><br><u>Format</u><br>• 5 groups with 8-10 staff as participants<br>• Each group is led by 3-5 facilitators from the CRTW, CAHOO secretariat and AIC (all part of the Singapore Ministry of Health)<br>• Lead facilitators and presenters: KLK, ST and KD<br><br><u>Accompanying materials to staff:</u><br>• 40-minute video summarising results (circulated before event)<br>• Event schedule<br>• One A4 sized page of priority statements (see Figure 2)<br><br><u>Accompanying materials to facilitators:</u><br>• Presentation slides of results, and Google form links and QR codes<br>• 18-page document for facilitators stationed at each table (Document contained prompting questions for facilitators to assist participants with the rating exercise, and also linked results to sections of the interviews, time motion studies, surveys and case note reviews)
Stage 5 of EBCD: Feedback event with clients and caregivers*	<u>Agenda</u><br>a) Overview and recap of results from interviews, time motion studies, surveys and case note reviews (+ Q&A)<br>b) Trigger film (+ Q&A)<br>c) Rating exercise 1: To select the top five priority statements from 15 (via A3 sized physical form)<br>d) Issue feedback form on event and vouchers<br><br><u>Format</u><br>• 4 groups with 4 clients and/or caregivers<br>• Each group is led by 3-4 facilitators from the CRTW, CAHOO secretariat and AIC (all part of the Singapore Ministry of Health)<br>• Lead facilitators and presenters: KLK, ST and KD<br><br><u>Accompanying materials to clients and caregivers:</u><br>• Event schedule

	• One A3 sized page of priority statements (see Figure 2)<br>• Stickers to paste on the A3 sized page of priority statements<br><br><u>Accompanying materials to facilitators:</u><br>• Presentation slides of results and trigger film<br>• 18-page document for facilitators stationed at each table (Document contained prompting questions for facilitators to assist participants with the rating exercise, and also linked results to sections of the interviews, time motion studies, surveys and case note reviews.)
Stage 6 of EBCD: Joint workshop	<u>Agenda</u><br>a) Overview and recap of results from rating exercises and comments from trigger film (+ Q&A)<br>b) Breakout group sessions, followed by group presentations<br>: Group 1 – focused on clinical practice guidelines with emphasis on meaningful goals, right help (tailored information) and skill sharing<br>: Group 2 – focused on professional development with emphasis on learning culture and supervision (in terms of supervisory guidance and quality of supervision)<br>: Group 3 – focused on community rehabilitation and support service recommendations with emphasis on easy navigation, flexibility and social options<br>c) Issue feedback form on event and vouchers (vouchers only for clients and caregivers)<br><br><u>Format</u><br>• 3 groups with 14-19 staff, clients and caregivers as participants<br>• Each group is led by 4-5 facilitators from the CRTW, CAHOO secretariat and AIC (all part of the Singapore Ministry of Health)<br>• Lead facilitators and presenters: KLK, ST and KD<br><br><u>Accompanying materials to staff, clients and caregivers:</u><br>• Event schedule<br>• One A4 sized page of priority statements (see Figure 2)<br><br><u>Accompanying materials to facilitators:</u><br>• Presentation slides of results and trigger film<br>• Butcher paper and pens, post it notes<br>• One slide for facilitators stationed at each table (Slide contained problem statements, questions and resources to help the facilitators guide the breakout group sessions)

*Event ran twice to accommodate the availability of clients and caregivers, so one event was on a weekday and another on a weekend.
EBCD, Experience Based Co-Design; Q&A, Question and Answer; CRTW, Community Rehabilitation Transformation Workgroup; CAHOO, Chief Allied Health Officer's Office; AIC, Agency for Integrated Care
