# Supplementary material for: Transforming Community‐Based Rehabilitation Services: A National Redesign Using Experience‐Based Co‐Design
Source: Health Expect. 2025 Jun 23;28(3):e70330. doi: 10.1111/hex.70330 (PMC12183464; doi:10.1111/hex.70330)
Supplement: Supplementary file 7 — Supporting Information 7. Additional data from interviews. [file HEX-28-e70330-s008.pdf]

Supplementary materials 7. Additional data from interviews

Table i. Characteristics of interview participants (n = 88 clients, caregivers and staff)
Table ii. Sample of interview quotes matched to the four broad themes of 1. Best practice care, 2. Person-centred care, 3. Allied Health Professional needs and 4. Service design, and 17 sub-themes

Table i. Characteristics of interview participants (n = 88 clients, caregivers and staff)

**Characteristics**	**Clients (n = 37)**	**Caregivers (n = 17)**	**Staff (n = 34)**
Gender, n (%)			
Female	18 (49%)	15 (88%)	27 (79%)
Male	19 (51%)	2 (12%)	7 (21%)
Age group, n (%)			
26 to 35 years	0	5 (29%)	23 (68%)
36 to 45 years	2 (5%)	1 (6%)	8 (23%)
46 to 55 years	3 (8%)	3 (18%)	2 (6%)
56 to 65 years	11 (30%)	6 (35%)	1 (3%)
66 to 75 years	12 (32%)	2 (12%)	0
76 to 85 years	4 (11%)	0	0
86 to 95 years	5 (14%)	0	0
Ethnicity, n (%)			
Chinese	27 (73%)	9 (52%)	22 (65%)
Eurasian	1 (3%)	2 (12%)	0
Indian	6 (16%)	1 (6%)	4 (12%)
Malay	2 (5%)	4 (24%)	3 (9%)
Others (e.g., Filipino, Myanmar)	1 (3%)	1 (6%)	5 (14%)
Highest educational qualification, n (%)			
O-level and/or A-level	3 (8%)	4 (24%)	-
Diploma	4 (11%)	1 (6%)	2 (6%)
Bachelor's degree	7 (19%)	4 (24%)	26 (76%)
Masters	2 (5%)	1 (6%)	6 (18%)
Others (e.g., Primary school, PhD)	21 (57%)	7 (40%)	0
Language most spoken, n (%)			
English	19 (51%)	13 (76%)	-
Chinese	14 (38%)	1 (6%)	-
Tamil	1 (3%)	0	-
Malay	1 (3%)	2 (12%)	-
Others (e.g., Hokkien dialect)	2 (5%)	1 (6%)	-
Marital status, n (%)			
Single	5 (14%)	4 (24%)	-
Married	28 (75%)	9 (52%)	-
Divorced/Widowed	4 (11%)	4 (24%)	-
Condition requiring rehabilitation, n (%)			
Deconditioning	9 (24%)	-	-
Hip fracture	8 (22%)	-	-
Stroke	20 (54%)	-	-
Housing type, n (%)			
Public housing (e.g., HDB flats)	31 (84%)	-	-
Private housing (e.g., Condominium)	6 (16%)	-	-
Relationship of caregiver to client, n (%)			
Spouse	-	6 (35%)	-
Child (e.g., Daughter, Son)	-	8 (47%)	-

Others (e.g., Niece, Grandchild)	-	3 (18%)	-
Health profession, n (%)			
PT	-	-	18 (53%)
OT	-	-	13 (38%)
SLT	-	-	2 (6%)
Social worker	-	-	1 (3%)
Years of experience, n (%)			
0 to 5	-	-	12 (35%)
6 to 10	-	-	15 (44%)
11 to 15	-	-	4 (12%)
16 to 20	-	-	2 (6%)
21 to 25	-	-	1 (3%)

PT, Physiotherapy; OT, Occupational Therapy; ST, Speech and Language Therapy

Table ii. Sample of interview quotes matched to the four broad themes of 1. Best practice care, 2. Person-centred care, 3. Allied Health Professional needs and 4. Service design, and 17 sub-themes

**Themes [Sub-themes]**	**Sample of interview quotes**
1. Best practice care	
[Evidence-based and person-centred]	*“I don't think we have guidelines though. There are protocols and guidelines, but I think everything is tailored towards the hospital setting. I don't know of any guidelines that's tailored to our community care [after leaving hospital]."* (Staff 38)<br><br>*“…best care for the patient… is giving them autonomy and asking them open-ended question so that they have time, and listening patiently, and not rushing them so that we can listen to what [the] client actually has to say and what's their priority, why they are here with us.”* (Staff 45)<br><br>*“[Best care] has to be definitely evidence-based practice. I think there is also a need to consider, not just the things that I want my clients to achieve, but rather also to discuss with the client, what are the goals that they want to achieve, and what the family wants to achieve.”* (Staff 47)<br><br>*“For my clients, apart from being competent, we also need to be compassionate…in the sense where the practice or care has to be client centric and then working towards their goal. But the underlying part, is always to make sure we keep up to date with the latest evidence.”* (Staff 54)
[Barriers and facilitators]	*"...another thing is to have sufficient time to conduct proper assessments and to schedule properly, to optimize staff-client ratio so we can have enough time during the therapy session itself to chat with the client rather than rushing… Another thing will be if there is incomplete information transfer between the referring centre and the new centre. For example, if they have missing therapy report or summary, it's also very difficult because for therapy report, sometimes it's just a very brief summary."* (Staff 30)<br><br>*“…access to information not only would improve your experience as a therapist, but the patient experience too... Because if the therapist has access to such information, they wouldn't have to keep repeating themselves. So they wouldn't be as drained to communicate…”* (Staff 47)<br><br>*"Training definitely will help because traditionally our training is very clinical focused. A lot of time we are attending courses based on diagnosis, based on more clinical protocol, not so much about training in the management of patient… and often times, we need more than just technical skills. Sometimes we need to learn some soft skills or how to manage patient, how to work with other disciplines, how to problem*

	solve a patient. Probably like workshops, or sometimes case discussions might help." (Staff 49)<br><br>*"We have Special Interest Groups (SIGs) where we can continue to build up on our interest in a specific area. When we have these activities or meet-ups with SIGs, it helps to give an opportunity to expand your clinical understanding. Knowing that there is support out there, there are people out there who are willing to share information and clinical expertise is helpful...sometimes my supervisors are busy. And knowing that this is a place where you meet other therapists who are also working in this community setting that's already done this and then we can connect with them to ask them about their experiences and share resources. Yeah, that's really helpful."* (Staff 52)<br><br>*"Staff are usually expected to do a lot of extra projects beyond clinical work. So sometimes we find it difficult to time manage between seeing patients for eight hours a day and coming up with time to do these extra projects. So there are times that we have to either carve out one or two hours on weekends or after work… it will be good if we have time carved out to do these type of things."* (Staff 61)<br><br>*"I think being aware of the resources out there in the community, like probably more sharing, a consolidated platform where everybody can showcase what are the resources in the community that we can tap onto... being aware of what are the latest updates on clinical care. That would be something that will help us with our treatment, to do something that is more effective and efficient rather than to keep doing the old-school way... Other things that personally restricts us from delivering best care, I think time. Everybody has a lot on their plate and you have to juggle a lot of projects. Manpower allocation as well... Communication between the community partners and the restructured [hospital] partners. In general, a network for a certain type of patient care, which is what One Rehab is trying to achieve, to be able to upskill, to be able to communicate better through a shared platform."* (Staff 68)<br><br>*"For therapists we have inservices every two weeks… Those topics are updated treatments for different types of conditions - that's one of the things we do to update ourselves. We also have WhatsApp group, sometimes we share with each other skills or certain YouTube videos on how we can help neuro patients. Then sometimes for our more senior staff, they have some experience in certain areas so they will call us to observe, e.g., making a splint. There's a mix of theory and practical for us to learn to improve our practice… Different conditions there will be a guideline on how we can help patients, but we have to pick out what's best for them because not everything will work. It can help, but a lot of work need to be done in terms of research to compile. In community we really see every condition, so it may be difficult but it definitely would help if there's a standard guideline for best practice. I think sometimes*

	also for us as therapists, we have a lot of admin to do so sometimes we may overlook the best treatment for them." (Staff 70)
2. Person-centred care	
[Meaningful goals]	*"He said it's also very good. They all teach him on the correct way to do things so that he can be more independent….[But] He said it's better but he has not achieved what he really wanted."* (Caregiver 26)<br><br>*"Therapist should ask what you want to achieve, this is very important. You need to ask the patients what goals they want to achieve, and discuss what can be done."* (Client 19)<br><br>*"It's like the best experiences we can have [is when] we see our patients improve a lot. Yeah, then they really appreciate what we did for them. And then like sometimes they return to work, they're able to walk again. They're able to do gardening again. They're able to swim again. When we see the progress that we did right, it's really very satisfying."* (Staff 62)<br><br>*"The occupational therapy is more on when I was able to walk right, I can go down buy lunch you know, from my flat go down, take the lift, go down, buy lunch with the quad stick and I was able to move around. Then like their exercise, also like tailored like you know, like going shopping, you pick things here and there, reach out all these thing. Yeah, that seems to help me a lot. Because when you go to supermarket, you need to reach for the things right?"* (Staff 65)
[Flexibility]	*"I feel that if I can schedule my own clients, I won't have any restrictions at all. I think that will help me a lot to handle my own schedule or if transportation is every hour kind and we can really schedule very nicely."* (Staff 51)<br><br>*"I do get to see patients and their family or their carers in their environment…So I get to see things realistically. So certain issues like maybe some of the physical challenges that they face like curbs, there are barriers that prevent patients from going into the toilet to shower and do their toileting needs… Because a lot of times maybe in day* rehab *centre even though they say that they are doing certain things at home, when you really go and see, what you suggested may not be realistic."* (Staff 55)<br><br>*"I'm more into management now, we do have a little bit of say into the projects that we want to do, and even like the equipment that we want to use and the system that we want to use. I do see being flexible about what we do being something that is positive. So at least it allows staff to be more proactive and then to come up with programmes or solutions to help to further improve the patient care process."* (Staff 68)<br><br>*"One of my biggest struggle was juggling more than maybe two to three clients at one time slot... I found that to be a big struggle because I think depending on their conditions it can be quite challenging to manage,*

	especially if we want to talk about person-centred care, because if their goals are ready for community integration or if you're talking about stroke, neuro rehab, it has to be very hands on one-on-one facilitation. But because of the way that the sessions are structured, three to one therapist, sometimes [we have] no choice but to get our therapy aides to help to manage another aspect of the session, or worse still, sometimes we have to put them on machines." (Staff 71)
[Right help]	*"What [social worker] did was will sit down and chat with me, not just about [the patient], but about how I'm going through mentally, and am I coping well. These are very important things [because] sometimes we just need somebody to talk to."* (Caregiver 3)<br><br>*"Can I get a psychiatrist to be included in the programme at [organisation]? I had also asked about a geriatrician…[to] look into the total and complete well-being of my mum. Because right now if I need to see a psychiatrist for my mum's mood or attitude and behavioural issues, where could I go to? Other than the rehab, we [need to] include psychiatrists and geriatrician so that I don't have to make visit to different areas… Can you help me refer my mum to psychiatrist because at the end of the day I think maybe I can not cope."* (Caregiver 4)<br><br>*"When I needed to refer my clients with chronic illnesses to specialist doctors, there's no direct access where we can link clients to them… So sometimes there were impressions that client had mini strokes or silent strokes at home or there could be a recurrence of excess fluid in the brain and the specialist had seen these clients more than a year ago. So, I wrote a letter addressed to a specialist doctor about the client's current condition and our impression. Then I asked the client to see GP or go to the polyclinic to pass the letter to the doctor. Only then the client can get the referral to the specialist. And then after that the clients told me that they had to wait a long time as they cannot get earlier appointment. That's one of the problems, that we don't have this direct access to specialists. [Interviewer]: I see so the time taken is very long to get the specialist. [Interviewee]: Yes, a very long time and much inconvenience for the clients and the family members."* (Staff 35)<br><br>*"[We need more] awareness of the resources in the community, and probably more sharing, or a consolidated platform where everybody can showcase the resources in the community that we can tap into… I think we [need] better communication between everybody."* (Staff 68)
[Caregivers]	*"It's time consuming because for me I try to arrange the time to bring him [my dad] there. I think it may not be easy for everyone to arrange this because one week three times. But if something needs to be done, then yes, I highly encourage people to go for rehab… The time for us is okay because I can adjust my time to fit [but others may not be able to]."* (Caregiver 9)<br><br>*"She won't be as motivated [to do rehabilitation with me]. She will say I am full of nonsense. She will say you're always making me do things no matter what I try to do."* (Caregiver 27)

	"Walking and stairs climbing are things that I don't do at home. I did not bother to do it. I respect the staff when I am in the centre, I do whatever they asked me to do. If I am at home, I would be lazy to move." (Client 9 - Translated)<br><br>*"Maybe we have some place we can access some video, how to perform certain exercises. If you cannot remember the final details, maybe this could help… because sometimes I come home, I forget, eh? Then I have to recall, then I have to ask my wife."* (Client 65)<br><br>*"The handover from hospital discharge [could be better]. I was quite thorough, and I made sure that before [my mother] was discharged that there's proper arrangements made. But sometimes, the doctors will say she looks ready for discharge. Now I say huh? But we're not ready on our side. You know, what equipment to get and all that, so that caused some anxiety. So maybe, more communication between the agencies making sure that the handover is better planned with the family members and caregivers."* (Caregiver 44)<br><br>*"There is a lot of stress on the caregivers... If we expect people to take up the role of caregiving, then are we ready to sacrifice some other aspects of stuff or provide resources for them? There are a lot more initiatives now which is great, but it's not necessarily targeted to the [caregiver]."* (Staff 31)
[Relationships]	*"The community service always have frequent changes. For example, the staff, it's never a permanent staff. So today can be somebody, then they [have a] shortage of manpower, then they will have another person. And then on top of that, I personally feel that they were just going through motions. They were not actually completely dedicated staff."* (Caregiver 3)<br><br>*"I think fundamentally whichever centre that attends to the patient, the professionals, play a very very important part. Not only from the skill point of view but in motivating [people]. The soft skills I feel is the one that really turn the patients around. …[My father] said the most memorable [part of therapy] is he and the staff can talk and joke around together, so it make his stay less painful... The staff here are so good. I've never seen this before. I mean they are good but somehow there is that different approach."* (Caregiver 26)<br><br>*"A lot of times we are running between patients, so they feel that our attention is divided. A lot of them [clients] actually do want more explanation. For example, even when we are doing exercises, they want to know why we are doing that… Because if we are struggling with little time, normally we are just doing things, doing the actions, doing the exercises without a lot of explanation such as why we are doing this."* (Staff 49)

	"I think most of the physiotherapists and the occupational therapists and their assistants were very helpful and very sincere…they really take care [of you]… so I feel very comfortable with them…and actually I look forward [to the sessions]…And they talk to you like you as youth; they remember my name… so these are the small things, like they can recognise you, they talk to you, they greet you. So, besides the therapy they give, these are the small things that make you happy, the experience there, so I value all these [things]… I mean you can have all the equipment in the world, but if you cannot connect to the patient, then it defeats the purpose." (Client 65)
3. Allied Health Professional needs	
[Learning culture]	*"Attending training sometimes is a little bit hard. It depends on how different organizations supports the training, whether do they expect the staff to attend it outside of their working hours or do they actually support it."* (Staff 49)<br><br>*"I would like to be updated, even in the clinical areas by attending recent updates or courses, but employer side it's not supportive in this."* (Staff 33)<br><br>*"If I am doing training work and then you see the new staff, being empowered to do their job, then they can sustain for longer in the industry. If not, we see high attrition rates."* (Staff 47)<br><br>*"I think a continued education program will help increase our knowledge and that will help us gain skills not just in handling chronic cases, but also acute cases, most especially when the chronic becomes acute…And then also [have] continued recognition of contributions made because it uplifts the spirit of the staff."* (Staff 35)<br><br>*"I guess the learning is a bit stagnant. Most of it is self-learning. If not, we do have biweekly in services by different colleagues. That's where I'll discuss our cases as well and learn from one another. I thought it could be more but also quite limited because there's only a few of us."* (Staff 56)
[Supervision]	*"Because I use different therapists [in various services]… I notice that young ones [therapists] may not be necessarily be as good as the older ones…So I think it's important for centres to have a good mix of older and younger ones. So that you get the kind of support that is needed. For example, I go to [a hospital and see] a very experience old therapist so that's where I actually spent a lot of time there because they are the ones that can help the most. You can talk to them and can even ask them questions [and] they have a good answers and good advice."* (Client 20)<br><br>*"[I would like] external sources of seniors that I can tap onto...I think having a mentor or a senior can help to improve our experience. So this*

	person may not be provided by your workplace...[and] this person will definitely not be involved in your appraisal, then you know there will not be as much judgment involved." (Staff 61)<br><br>*"For a social worker, smaller organisations can perhaps only can offer 1 or 2 social workers to work in this organisation. So when I started off in [organisation] I'm the only social worker here. But imagine that if without any past experiences, it's very challenging for a fresh graduate to come in and work in this setting, because there's no one that I can ask as the majority of the supervisors are all in hospital. Then it's really, really challenging for lone individual, and then [the service] expects everything to be done perfectly... You are a one-man show and you need to cover most of the things."* (Staff 42)<br><br>*"I not only depend on my supervisors, but there is open communication between other therapists as well. I get to learn from another therapist's perspective...[but] sometimes my supervisors are busy."* (Staff 52)
[Time allocation]	*"We have a lot of admin to do so sometimes we may overlook the best treatment for [our clients]. Sometimes we'll just repeat whatever has been done previously and change a little bit, because we need to meet a certain deadline for this project. Or pass to the therapy assistant to help us see [clients], to come in and out to check on the patient, whether they are doing well."* (Staff 70)<br><br>*"Normally the first 20 minutes I do the bicycle, cycling. After that they bring you to sit up and sit down for leg muscles. The leg press. By the time you finished your leg press, you got no time already [to do anything else]."* (Client 66)<br><br>*"I think all healthcare professionals will struggle with, is having timeout throughout your working hours to really get updated on all these current practices, like even just having half an hour just for you to do research, for you to not be doing work-related things. But then again, it's not practical because to have that half an hour means you are also not doing something else then you still have to stay back after work."* (Staff 20)<br><br>*"Everybody is too busy, no chance to/really no time to even do research in our own setting how to do like cross sector research? Too lofty a thought. I think if we can do rotations, happy already."* (Staff 36)
[Career progression]	*"I think the important thing…[is] the mentoring and progression of the staff. In general, in the hospitals because it's very systemic, it's very structured, so the resources given is also there… When you're in the community you are no longer under MOH and then the resources is based on the different agency resources itself."* (Staff 42)<br><br>*"I do know that at the hospital we have certain edge simply because of opportunities. I think it is very important to level the playing field… I think it should be an actual rotation to different specializations. Specialties, you know, and I think community, sub-acute and acute is*

	actually very different. I worked across, so I know. There should be opportunities to work across." (Staff 36)<br><br>*"Things that might not attract people to the community could be payroll. Maybe professional career progression [as well]."* (Staff 69)<br><br>*"Salaries will be definitely lesser as compared to the acute hospital. So you are just one-man show and then you need to cover most of the things."* (Staff 42)<br><br>*"I find it a bit difficult to get manpower these days…we don't get enough applicants to join the community sector… we find more younger applicants, like those who are still under AHPC supervision, because I think the younger graduates, they have placements, so they are very familiar with what's in the community… But if you want to look for like more experienced therapists, it is very hard…When it gets hard to attract manpower into the organisation then it ends up that the therapist who is there have to juggle between conducting programmes, work, paperwork, training and everything."* (Staff 69)
[Skill sharing]	*"In the community, let's say you are in the smaller organisations, you do not have access to doctors or nurses this kind of thing. So sometimes you would need to transdisciplinary practice a lot. Being in a community sometimes you do not have access to all your other multidisciplinary partners. So, most of the things you have to take on yourself and sometimes is overwhelming for the therapists, because this actually not in their training."* (Staff 68)<br><br>*"I actually don't need counselling support. Firstly I think I don't have such needs, and secondly I think my therapists have provided psychological support. They will check if I can cope with the situation, as well as whether I face any issue with my family and kids. If they realise I am feeling sad/down in conversation, they will try to counsel me. I think their job is not easy, because not only helping you with your function, they will also help you with your life, I can feel how responsible they are in this."* (Client 23- Translated)
4. Service design	
[Funding]	*"Now I am actually paying [for therapy] because I am [considered] a maintenance [client]. I wonder whether I can get a [discount] or something because I am no longer working. I think you should try to help [and make it] no need to pay*." (Client 67)<br><br>*"If we discharge them, where do they go? So we are actually struggling with discharging because a lot of them don't want to get discharged from the service. There are some clients I feel that they don't really need staff to help them actually. They can be discharged but the thing is they don't want to be. So we are trying home exercise and all that, but they still feel the need to want to come and for some of them, it's may be better because then they don't face social isolation. We don't need a*

	therapist or rehab centre to do that, but where else can we actually discharge them to?" (Staff 51)<br><br>*"I think some of the struggles will also be, after some of our clients finish their active rehab phase, then they want to go into that maintenance rehab program. Because that maintenance rehab program is funded by a different fund, so I don't think they can continue paying by Medisave, but it will be by cash."* (Staff 71)<br><br>*"Some of the maintenance cases we cannot discharge, so because we're worried that if we discharge them they might decline. If we have more social enterprise help, we can discharge through them. So we can accept more active cases."* (Staff 53)<br><br>*"Why [patients] want to stay on the rehab is because the rehab service they can pay using Medisave. Whereas if they change to maintenance exercise, which they probably could, but that service does not allow them to use Medisave. So then they will fight to stay on the rehab program for a long time instead of converting to the maintenance exercise… The client will then reject the other services [because of the cost]… I do think maybe this funding can help us a little bit in terms of transitioning patients to the right service."* (Staff 47)<br><br>*"And then the other thing would be after their discharge from one centre, right? Sometimes some of them really still want to have rehab. But they thought they can't afford private. There's so many of these kinds of cases. So then they want to know if there's any way they can still get back into the system…But I tell you, at non-profit organization there are so many of them who are discharged from service somewhere but they actually come and ask us. They say this person really still needs physio or still needs ST, where can I go?"* (Staff 36)<br><br>*"I think number one [challenge] is usually funding cause funding is limited. We have to source our own grants, then with grants you have KPIs to meet. So as a therapist you need to be aware of the financial limitations and try source out for your own financial means [which takes time]. We try in the community to be more integrated in our interventions, but I think the funding structure from [organisation] is a bit segregated [as] there are only certain organisations that are eligible for the home therapy funding. If you were to incorporate home-based interventions in our DRC setting, I think [organisation] are not able to provide their funding for home therapy. So they want us to refer out these patients to the home therapy providers, which I feel is a bit contradicting if you want an integrated therapy process… So, funding structures affect how far you can broaden your programme."* (Staff 69)<br><br>*"I don't feel that it [services] is that integrated you know. Every organisation I was told has their own system and I don't understand why. The community hospital will tell me oh no we are different from acute. Then the current therapy centre will say oh no we are different.*

	So every time it is different. And I will go through the same thing again. If you franchise you have your financial system, a standard format, standard funding. You just got to answer for your consumables. Isn't that easier to administer?" (Caregiver 27)<br><br>*"Sometimes the client has rehab potential but because of financial issues, they cannot come in. We try to help those who really cannot afford rehab by applying for special funding… Because even though they may have 80 percent subsidy, they still need to pay about 20%... I think [some] client are more willing to come [if] they pay cheaper."* (Staff 53)
[Social options]	*"These sessions allow her opportunities to talk to other people about things that she is familiar with. I feel that this will lift her spirits."* (Caregiver 57)<br><br>*"At least you have something to do [by going to the centre]."* (Client 2 - Translated)<br><br>*"Most of the elderly do enjoy you sitting down and talking to them. More than the exercise itself. I feel like they enjoy talking to you more than doing the exercise."* (Staff 51)<br><br>*"70% are very interested to come [to the community service] because there's nothing to do at home."* (Staff 40)<br><br>*"There is no activity centre… for the same type of stroke patients. Allowing them to interact and also share the same interests. My mum loves gardening. My mum loves needlework. My mum loves cooking, so is there such an interest group? Is it not in this [the community organisation] or has she got to go to different places to join in these interest groups?"* (Caregiver 4)<br><br>*"I think the community rehab here is good to help him mingle with the staff here, friendly for him at least he can go out, 1 week 3 times here. Rather in hospital, he lies down right, [when] he is at home also most of the time lie down watch TV or sit down."* (Caregiver 34)<br><br>*"I was also considering a drop in centre or day care for mum. Because she looks forward to rehab [but] it's only an hour three times a week. And if I am working or if I'm [living abroad] then its still a lot for dad and the helper to manage…[but] the drop-in centre usually starts from 9 to about 5 and there is really no place for the elderly to rest, so they are sitting, you know, for so many hours. So, Mum had back ache and I can imagine so do the others, and you know water retention in their feet, if they cannot have a space to lie down in a reclining state..."* (Caregiver 44)<br><br>*"More programmes, more fun programmes… Have more entertainment, let the elderly have fun is good."* (Client 15 - Translated)

	"We recommended was to pay the volunteers. They are not staff but give them some allowance. So, for the active seniors who retire and got nothing to do, instead of walking the streets and get dementia [they could volunteer to support others]." (Caregiver 27)<br><br>*"..doing more home visits to build up their confidence in the actual ground at their home or in the community setting…if we got to bring them out into their house and to their community to practice all those activities, then it will definitely need more than one session [but] it would be ideal to do that. Especially when we are at the end of their rehab, and to maybe build up their confidence to self-manage."* (Staff 49)<br><br>*"I think another struggle that some of us ….will be the discharge planning…the goals have already been achieved but because sometimes our clients or their family members may be very anxious about discharging them from our healthcare system completely….there are not as many other community resources that we can refer them on to. I don't know whether it's a lack of knowledge on our own end, because sometimes when we research not all the services may pop up, we are not too sure. We do know that [organisation] also does have a list of comm resources."* (Staff 71)
[Easy navigation]	*"There must be somebody who will guide us along the way. And there wasn't anybody. I went to hospital, they gave me a couple of brochures, please go through. You know when this thing happens, you're not in your right frame of mind to sit down and read through these brochures."* (Caregiver 3)<br><br>*"There are many different people involved in the whole process. There's also a medical social worker that is apparently different for different hospitals... Same thing with the therapist. So sometimes when she was transferred not all information was passed and I was talking to different people asking the same information. So perhaps better transfer of information between the institutions."* (Caregiver 44)<br><br>*"A lot time wastage during the initial assessment [because] you go in and try to ask everything from scratch again [from the] patients or the family. If such information is given to us that'll be easier for us to follow through… It's easier for patients too, as they don't have to answer so many questions."* (Staff 55)<br><br>*"Sometimes the [patients and caregivers] are quite lost... That means they are entirely dependent on the goodwill and the dedication of the healthcare professionals to know what services are out there. [They are] very passive and just wait for the medical social worker to refer through [organisation] then they just wait. I think caregivers, patients do want to understand more of what to expect and what is to be expected in each part of the journey."* (Staff 39)

	"[We need more] awareness of the resources in the community, and probably more sharing, or a consolidated platform where everybody can showcase the resources in the community that we can tap into… I think we [need] better communication between everybody." (Staff 68)
[Transport]	*"Transport might be issue…because she's on a walking frame, we need someone to bring her up to our house. ..they don't have the manpower to bring my mom up [to the flat]. They told us there are some of the elderlies, they will just wait at the void deck…and wait for the children to come back."* (Caregiver 1)<br><br>*"Sometimes got the time management (issue) because we provide transport service. Sometimes the transport delay so the next session clients also need to delay. So we also have to extend our time for them because nowadays we deliver just one hour service only, so the transport delay is not the client issue, we have to extend the time to complete one hour, so the next scheduled client needs to wait, sometimes the clients angry."* (Staff 48)<br><br>*"For Community Organisation rehab, I think mostly [people attend] because of the transportation that we provide. That time slot is just more popular with clients and because they have nobody to bring them to rehab, that is the only time slot that they're available."* (Staff 51)
[Centre design]	*"Everything is good, facilities are clean and comfortable looking."* (Caregiver 57)<br><br>*"My first impression didn't feel good. I wasn't sure whether the equipment was effective. …So actually the whole centre, it was quite depressing for me when I first went. .And my mother is very affected. And also the toilet for example, I think that place need help to refurbish."* (Caregiver 27)
