# Supplementary material for: Transforming Community‐Based Rehabilitation Services: A National Redesign Using Experience‐Based Co‐Design
Source: Health Expect. 2025 Jun 23;28(3):e70330. doi: 10.1111/hex.70330 (PMC12183464; doi:10.1111/hex.70330)

Supplementary materials 8. Additional data from surveys and case note reviews

Clinical practice (Data from clinical survey, n = 95 allied health professionals)
Table i. Characteristics of clinical survey respondents
Table ii. Frequency of stroke assessments reported by allied health professionals
Table iii. Frequency of frailty assessments reported by allied health professionals
Table iv. Frequency of hip fracture assessments reported by allied health professionals
Table v. Frequency of stroke treatments reported by allied health professionals
Table vi. Frequency of frailty treatments reported by allied health professionals
Table vii. Frequency of hip fracture treatments reported by allied health professionals

Organisational practice (Data from organizational survey, n = 29 day rehabilitation centres)
Table viii. Caseload across day rehabilitation centres
Figure i. Manpower allocation across day rehabilitation centres
Figure ii. Practices surrounding active and maintenance rehabilitation

Skills sharing (Data from clinical survey, n = 95 allied health professionals and organizational survey, n = 29 day rehabilitation centres)
Figure iii. Practice of skills-sharing reported by A) allied health professionals (n = 95) and B) rehabilitation leaders (n = 29)
Figure iv. Clinical areas where skills sharing is perceived to be possible by A) allied health professionals (n = 95), B) physiotherapists (n = 59), C) occupational therapists (n = 25) and D) speech and language therapists (n = 11)
Figure v. Availability of processes at site for skills-sharing reported by rehabilitation leaders (n = 29)

Table i. Characteristics of clinical survey respondents

Characteristics	AHPs who completed survey (n = 95)	AHPs who did not complete survey (n = 22)
Health profession, n (%)		
PT	59 (62%)	11 (50%)
OT	25 (26%)	10 (45%)
ST	11 (12%)	1 (5%)
Years of experience, n (%)		
Less than 5	26 (27%)	11 (50%)
5 to 10	40 (42%)	3 (14%)
More than 10	29 (31%)	8 (36%)
Awareness of stroke CPGs, n (%)		
Yes	86 (91%)	17 (77%)
No	9 (9%)	5 (23%)
Frequency of adherence to stroke CPGs, n (%)		
Often/Always	62 (65%)	12 (55%)
Sometimes	26 (27%)	6 (27%)
Rarely/Never	7 (7%)	4 (18%)
Awareness of frailty CPGs, n (%)		
Yes	49 (52%)	10 (45%)
No	46 (48%)	12 (55%)
Frequency of adherence to frailty CPGs, n (%)		
Often/Always	44 (46%)	6 (27%)
Sometimes	18 (19%)	8 (36%)
Rarely/Never	33 (35%)	8 (36%)
Awareness of hip fracture CPGs, n (%)		
Yes	69 (73%)	15 (68%)
No	26 (27%)	7 (32%)
Frequency of adherence to hip fracture CPGs, n (%)		
Often/Always	56 (59%)	11 (50%)
Sometimes	13 (14%)	5 (23%)
Rarely/Never	26 (27%)	6 (27%)

AHP, Allied Health Professionals; PT, Physiotherapy; OT, Occupational Therapy; ST, Speech and Language Therapy; CPGs, Clinical Practice Guidelines

Table ii. Frequency of stroke assessments reported by allied health professionals*

Assessments	Profession	Often/Always	Sometimes	Rarely/Never
MMSE	PT	4 (7%)	5 (8%)	50 (85%)
MoCA	PT	3 (5%)	1 (2%)	55 (93%)
HADS	PT	1 (2%)	2 (3%)	56 (95%)
PHQ-4	PT	1 (2%)	1 (2%)	57 (97%)
MSAS	PT	4 (7%)	3 (5%)	52 (88%)
MAS	PT	17 (29%)	6 (10%)	36 (61%)
BBS	PT	44 (75%)	13 (22%)	2 (3%)
TUG	PT	53 (90%)	5 (8%)	1 (2%)
5 x STS	PT	43 (73%)	11 (19%)	5 (8%)
ST	PT	11 (19%)	13 (22%)	35 (59%)
MMSE	OT	9 (36%)	10 (40%)	6 (24%)
MoCA	OT	12 (48%)	5 (20%)	8 (32%)
HADS	OT	0 (0%)	0 (0%)	25 (100%)
PHQ-4	OT	0 (0%)	0 (0%)	25 (100%)
ARAT	OT	0 (0%)	0 (0%)	25 (100%)
FMA-UE	OT	13 (52%)	3 (12%)	9 (36%)
9HPT	OT	4 (16%)	3 (12%)	18 (72%)
MMSE	ST	0 (0%)	0 (0%)	11 (100%)
MoCA	ST	1 (9%)	2 (18%)	8 (73%)
HADS	ST	0 (0%)	0 (0%)	11 (100%)
PHQ-4	ST	0 (0%)	0 (0%)	11 (100%)
BDAE	ST	1 (9%)	2 (18%)	8 (73%)
CAT	ST	1 (9%)	2 (18%)	8 (73%)
WAB	ST	5 (45%)	2 (18%)	4 (36%)
PALPA	ST	2 (18%)	2 (18%)	7 (64%)
IDDSI	ST	9 (82%)	2 (18%)	0 (0%)

ARAT, Action Research Arm Test; BDAE, Boston Diagnostic Aphasia Examination; BBS, Berg Balance Scale; CAT, Comprehensive Aphasia Test; FMA-UE, Fugl Meyer Assessment-Upper Extremity; HADS, Hospital Anxiety and Depression Scale; IDDSI, IDDSI Functional Diet Scale; PALPA, Psycholinguistics Assessments of Language Processing Abilities; PHQ-4, Patient Health Questionnaire-4; MSAS, Mobility Scale for Acute Stroke; MAS, Motor Assessment Scale; MMSE, Mini–Mental State Examination; MoCA, Montreal Cognitive Assessment; 9HPT, Nine Hole Peg Test; ST, Step Test; TUG, Timed Up and Go; WAB, Western Aphasia Battery; 5 x STS, 5 x Sit-to-Stand Test; PT, Physiotherapy; OT, Occupational Therapy; ST, Speech and Language Therapy

*Allied health professionals include PT (n = 59), OT (n = 25) and ST (n = 11).

Table iii. Frequency of frailty assessments reported by allied health professionals*

Assessments	Profession	Often/Always	Sometimes	Rarely/Never
FFP	PT	5 (8%)	3 (5%)	51 (86%)
CFS	PT	8 (14%)	7 (12%)	44 (75%)
FRAIL	PT	5 (8%)	7 (12%)	47 (80%)
PRISMA-7	PT	1 (2%)	0 (0%)	58 (98%)
TFI	PT	1 (2%)	0 (0%)	58 (98%)
EFS	PT	1 (2%)	0 (0%)	58 (98%)
SOF	PT	1 (2%)	0 (0%)	58 (98%)
RGA	PT	1 (2%)	0 (0%)	58 (98%)
CGA	PT	1 (2%)	0 (0%)	58 (98%)
ICOPE	PT	1 (2%)	0 (0%)	58 (98%)
Kihon	PT	1 (2%)	0 (0%)	58 (98%)
MMSE	PT	7 (12%)	8 (14%)	44 (75%)
MoCA	PT	4 (7%)	4 (7%)	51 (86%)
SPPB	PT	22 (37%)	7 (12%)	30 (51%)
TUG	PT	51 (86%)	7 (12%)	1 (2%)
Grip strength	PT	20 (34%)	9 (15%)	30 (51%)
IDDSI	PT	2 (3%)	0 (0%)	57 (97%)
FOIS	PT	2 (3%)	0 (0%)	57 (97%)
FFP	OT	0 (0%)	0 (0%)	25 (100%)
CFS	OT	1 (4%)	3 (12%)	21 (84%)
FRAIL	OT	0 (0%)	0 (0%)	25 (100%)
PRISMA-7	OT	0 (0%)	0 (0%)	25 (100%)
TFI	OT	0 (0%)	0 (0%)	25 (100%)
EFS	OT	0 (0%)	0 (0%)	25 (100%)
SOF	OT	0 (0%)	0 (0%)	25 (100%)
RGA	OT	0 (0%)	0 (0%)	25 (100%)
CGA	OT	0 (0%)	0 (0%)	25 (100%)
ICOPE	OT	0 (0%)	0 (0%)	25 (100%)
Kihon	OT	0 (0%)	0 (0%)	25 (100%)
MMSE	OT	10 (40%)	8 (32%)	7 (28%)
MoCA	OT	6 (24%)	10 (40%)	9 (36%)
SPPB	OT	3 (12%)	1 (4%)	21 (84%)
TUG	OT	5 (20%)	0 (0%)	20 (80%)
Grip strength	OT	9 (36%)	7 (28%)	9 (36%)
IDDSI	OT	0 (0%)	0 (0%)	25 (100%)
FOIS	OT	0 (0%)	0 (0%)	25 (100%)
FFP	ST	0 (0%)	0 (0%)	11 (100%)
CFS	ST	0 (0%)	0 (0%)	11 (100%)
FRAIL	ST	0 (0%)	0 (0%)	11 (100%)
PRISMA-7	ST	0 (0%)	0 (0%)	11 (100%)
TFI	ST	0 (0%)	0 (0%)	11 (100%)
EFS	ST	0 (0%)	0 (0%)	11 (100%)
SOF	ST	0 (0%)	0 (0%)	11 (100%)
RGA	ST	0 (0%)	0 (0%)	11 (100%)
CGA	ST	0 (0%)	0 (0%)	11 (100%)

ICOPE	ST	0 (0%)	0 (0%)	11 (100%)
Kihon	ST	0 (0%)	0 (0%)	11 (100%)
MMSE	ST	0 (0%)	0 (0%)	11 (100%)
MoCA	ST	1 (9%)	0 (0%)	10 (91%)
SPPB	ST	0 (0%)	0 (0%)	11 (100%)
TUG	ST	0 (0%)	0 (0%)	11 (100%)
Grip strength	ST	0 (0%)	0 (0%)	11 (100%)
IDDSI	ST	8 (73%)	2 (18%)	1 (9%)
FOIS	ST	9 (82%)	2 (18%)	0 (0%)

FFP, Fried's Frailty Phenotype; CFS, Clinical Frailty Scale; FRAIL, Fatigue, Resistance, Ambulation, Illness, and Loss of weight scale; PRISMA-7, Program of Research to Integrate Services for the Maintenance of Autonomy-7; TFI, Tilburg Fragility Index; EFS, Edmonton Frailty Scale; SOF, Study of Osteoporotic Fractures index; RGA, Rapid Geriatric Assessment; CGA, Comprehensive Geriatric Assessment; ICOPE, Integrated Care for Older People instrument; Kihon, Kihon checklist; MMSE, Mini-Metal State Examination; MoCA, Montreal Cognitive Assessment; SPPB, Short Physical Performance Battery; TUG, Timed-Up-and-Go; Grip strength; IDDSI, IDDSI Functional Diet Scale; FOIS, Functional Oral Intake Scale; PT, Physiotherapy; OT, Occupational Therapy; ST, Speech and Language Therapy

*Allied health professionals include PT (n = 59), OT (n = 25) and ST (n = 11).

Table iv. Frequency of hip fracture assessments reported by allied health professionals*

Assessments	Profession	Often/Always	Sometimes	Rarely/Never
Hip mm strength	PT	57 (97%)	0 (0%)	2 (3%)
Other LL mm strength	PT	57 (97%)	0 (0%)	2 (3%)
SPPB	PT	25 (42%)	6 (10%)	28 (47%)
TUG	PT	52 (88%)	5 (8%)	2 (3%)
SCT	PT	36 (61%)	10 (17%)	13 (22%)
6MWT	PT	37 (63%)	13 (22%)	9 (15%)
Hip mm strength	OT	7 (28%)	4 (16%)	14 (56%)
Other LL mm strength	OT	6 (24%)	5 (20%)	14 (56%)
SPPB	OT	1 (4%)	2 (8%)	22 (88%)
TUG	OT	3 (12%)	1 (4%)	21 (84%)
SCT	OT	1 (4%)	1 (4%)	23 (92%)
6MWT	OT	3 (12%)	2 (8%)	20 (80%)

Hip mm strength, Hip muscle strength; Other LL mm strength, Other lower leg muscle strength; SPPB, Short Physical Performance Battery; TUG, Timed-Up-and-Go; SCT, Stair Climb Test; 6MWT, Six Minute Walk Test; PT, Physiotherapy; OT, Occupational Therapy
*Allied health professionals include PT (n = 59), OT (n = 25) and ST (n = 11).

Table v. Frequency of stroke treatments reported by allied health professionals*

Treatments	Profession	Often/ Always	Sometimes	Rarely/ Never
Education on neuroplasticity and rehabilitation journey	PT	40 (68%)	14 (24%)	5 (8%)
Education on discharge planning and transfer of care	PT	50 (85%)	6 (10%)	3 (5%)
Caregiver training	PT	53 (90%)	5 (8%)	1 (2%)
Education on self-management	PT	50 (85%)	7 (12%)	2 (3%)
Provision of information re: peer support to client and/or family (e.g., local stroke support group)	PT	23 (39%)	14 (24%)	22 (37%)
Goal-setting	PT	58 (98%)	1 (2%)	0 (0%)
Strength training and/or progressive resistance training for arm/leg weakness	PT	56 (95%)	2 (3%)	1 (2%)
Electrical stimulation for less than antigravity strength in leg	PT	14 (24%)	19 (32%)	26 (44%)
Electrical stimulation for less than antigravity strength in arm	PT	7 (12%)	14 (24%)	38 (64%)
Sensory-specific training for sensory loss	PT	11 (19%)	16 (27%)	32 (54%)
Cardiorespiratory fitness training (e.g., walking, arm or leg cycling at moderate intensity)	PT	54 (92%)	5 (8%)	0 (0%)
Repetitive task-specific practice of sitting, standing up, standing and/or walking	PT	58 (98%)	1 (2%)	0 (0%)
Repetitive task-specific practice of upper limb activity	PT	29 (49%)	12 (20%)	18 (31%)
Use of virtual reality training for standing and/or walking	PT	12 (20%)	9 (15%)	38 (64%)
Use of force platform for standing balance training	PT	15 (25%)	9 (15%)	35 (59%)
Use of electromechanical assisted device for standing and/or walking (e.g., body weight support, robotics)	PT	10 (17%)	18 (31%)	31 (53%)
Lower limb orthoses for walking	PT	24 (41%)	21 (36%)	14 (24%)
Use of virtual reality training for upper limb activity	PT	4 (7%)	6 (10%)	49 (83%)
Use of electromechanical assisted device for upper limb activity (e.g., robotics)	PT	3 (5%)	4 (7%)	52 (88%)
Upper limb orthoses/splints for contracture	PT	5 (8%)	13 (22%)	41 (69%)
Constraint-induced movement therapy for upper limb activity	PT	4 (7%)	8 (14%)	47 (80%)

in those with some active wrist and finger extension				
Mental practice with active motor training for upper limb activity in those with mild to moderate arm weakness	PT	6 (10%)	9 (15%)	44 (75%)
Mirror therapy as adjunct to routine therapy for upper limb activity in those with mild to moderate arm weakness and/or neglect	PT	7 (12%)	9 (15%)	43 (73%)
Recommendation of acupuncture for pain	PT	3 (5%)	7 (12%)	49 (83%)
Recommendation of acupuncture for activities of daily living and spasticity	PT	2 (3%)	9 (15%)	48 (81%)
Referral for non-invasive brain stimulation (transcranial direct current stimulation or repetitive transcranial magnetic stimulation)	PT	1 (2%)	1 (2%)	57 (97%)
Referral to upstream providers for Botulinum Toxin A for spasticity	PT	6 (10%)	7 (12%)	46 (78%)
Adjunct therapies for clients who have received Botulinum Toxin A (e.g., electrical stimulation)	PT	6 (10%)	9 (15%)	44 (75%)
Routine use of stretch for spasticity and/or contracture	PT	39 (66%)	11 (19%)	9 (15%)
Electrical stimulation for those at risk of shoulder subluxation	PT	7 (12%)	12 (20%)	40 (68%)
Shoulder strapping for those at risk of shoulder subluxation	PT	18 (31%)	9 (15%)	32 (54%)
Electrical stimulation for those with shoulder pain	PT	7 (12%)	9 (15%)	43 (73%)
Shoulder strapping for those with shoulder pain	PT	10 (17%)	10 (17%)	39 (66%)
Referral to upstream providers for shoulder injections and/or Botulinum Toxin A for those with shoulder pain	PT	3 (5%)	5 (8%)	51 (86%)
Multifactorial interventions, such as, an individually prescribed exercise program and advice on safety for falls	PT	52 (88%)	4 (7%)	3 (5%)
Outdoors mobility training	PT	30 (51%)	19 (32%)	10 (17%)

Visuoperceptual rehabilitation (e.g., eye patching, mental practice, visual scanning training)	PT	9 (15%)	15 (25%)	35 (59%)
Gesture training, strategy training and/or errorless learning for limb apraxia	PT	4 (7%)	8 (14%)	47 (80%)
Referral to other providers for driving simulation	PT	8 (14%)	4 (7%)	47 (80%)
Assessment and/or assistance with return to work for those who wish to return to work	PT	14 (24%)	9 (15%)	36 (61%)
Education on neuroplasticity and rehabilitation journey	OT	18 (72%)	3 (12%)	4 (16%)
Education on discharge planning and transfer of care	OT	22 (88%)	0 (0%)	3 (12%)
Caregiver training	OT	21 (84%)	4 (16%)	0 (0%)
Education on self-management	OT	22 (88%)	3 (12%)	0 (0%)
Provision of information re: peer support to client and/or family (e.g., local stroke support group)	OT	4 (16%)	15 (60%)	6 (24%)
Goal-setting	OT	25 (100%)	0 (0%)	0 (0%)
Strength training and/or progressive resistance training for arm/leg weakness	OT	23 (92%)	2 (8%)	0 (0%)
Electrical stimulation for less than antigravity strength in leg	OT	1 (4%)	4 (16%)	20 (80%)
Electrical stimulation for less than antigravity strength in arm	OT	13 (52%)	7 (28%)	5 (20%)
Sensory-specific training for sensory loss	OT	8 (32%)	11 (44%)	6 (24%)
Cardiorespiratory fitness training (e.g., walking, arm or leg cycling at moderate intensity)	OT	17 (68%)	4 (16%)	4 (16%)
Repetitive task-specific practice of sitting, standing up, standing and/or walking	OT	20 (80%)	4 (16%)	1 (4%)
Repetitive task-specific practice of upper limb activity	OT	23 (92%)	2 (8%)	0 (0%)
Use of virtual reality training for standing and/or walking	OT	2 (8%)	7 (28%)	16 (64%)
Use of force platform for standing balance training	OT	2 (8%)	1 (4%)	22 (88%)
Use of electromechanical assisted device for standing and/or walking (e.g., body weight support, robotics)	OT	1 (4%)	3 (12%)	21 (84%)
Lower limb orthoses for walking	OT	2 (8%)	3 (12%)	20 (80%)

Use of virtual reality training for upper limb activity	OT	1 (4%)	8 (32%)	16 (64%)
Use of electromechanical assisted device for upper limb activity (e.g., robotics)	OT	0 (0%)	3 (12%)	22 (88%)
Upper limb orthoses/splints for contracture	OT	2 (8%)	8 (32%)	15 (60%)
Constraint-induced movement therapy for upper limb activity in those with some active wrist and finger extension	OT	2 (8%)	6 (24%)	17 (68%)
Mental practice with active motor training for upper limb activity in those with mild to moderate arm weakness	OT	7 (28%)	8 (32%)	10 (40%)
Mirror therapy as adjunct to routine therapy for upper limb activity in those with mild to moderate arm weakness and/or neglect	OT	1 (4%)	8 (32%)	16 (64%)
Recommendation of acupuncture for pain	OT	1 (4%)	2 (8%)	22 (88%)
Recommendation of acupuncture for activities of daily living and spasticity	OT	1 (4%)	0 (0%)	24 (96%)
Referral for non-invasive brain stimulation (transcranial direct current stimulation or repetitive transcranial magnetic stimulation)	OT	0 (0%)	0 (0%)	25 (100%)
Referral to upstream providers for Botulinum Toxin A for spasticity	OT	1 (4%)	7 (28%)	17 (68%)
Adjunct therapies for clients who have received Botulinum Toxin A (e.g., electrical stimulation)	OT	3 (12%)	5 (20%)	17 (68%)
Routine use of stretch for spasticity and/or contracture	OT	21 (84%)	4 (16%)	0 (0%)
Electrical stimulation for those at risk of shoulder subluxation	OT	15 (60%)	5 (20%)	5 (20%)
Shoulder strapping for those at risk of shoulder subluxation	OT	4 (16%)	3 (12%)	18 (72%)
Electrical stimulation for those with shoulder pain	OT	6 (24%)	5 (20%)	14 (56%)
Shoulder strapping for those with shoulder pain	OT	2 (8%)	2 (8%)	21 (84%)
Referral to upstream providers for shoulder injections and/or	OT	2 (8%)	1 (4%)	22 (88%)

Botulinum Toxin A for those with shoulder pain				
Multifactorial interventions, such as, an individually prescribed exercise program and advice on safety for falls	OT	19 (76%)	2 (8%)	4 (16%)
Outdoors mobility training	OT	13 (52%)	10 (40%)	2 (8%)
Visuoperceptual rehabilitation (e.g., eye patching, mental practice, visual scanning training)	OT	7 (28%)	12 (48%)	6 (24%)
Gesture training, strategy training and/or errorless learning for limb apraxia	OT	5 (20%)	5 (20%)	15 (60%)
Referral to other providers for driving simulation	OT	7 (28%)	8 (32%)	10 (40%)
Assessment and/or assistance with return to work for those who wish to return to work	OT	8 (32%)	9 (36%)	8 (32%)
Meta-cognitive strategy +/- cognitive training for executive function	OT	9 (36%)	11 (44%)	5 (20%)
Cognitive rehabilitation (remediation pen & paper tasks) for neglect	OT	10 (40%)	9 (36%)	6 (24%)
Cognitive rehabilitation (remediation leveraging on technology apps)	OT	8 (32%)	6 (24%)	11 (44%)
Cognitive rehabilitation (remediation functional tasks training)	OT	13 (52%)	9 (36%)	3 (12%)
Cognitive rehabilitation (compensatory strategies)	OT	14 (56%)	7 (28%)	4 (16%)
Provide targeted OT interventions in the areas of self-care and instrumental ADLs (e.g., grocery shopping, paying bills etc.)	OT	20 (80%)	5 (20%)	0 (0%)
Provide targeted OT interventions in the areas of productivity, social participation, and leisure	OT	18 (72%)	7 (28%)	0 (0%)
Education on neuroplasticity and rehabilitation journey	ST	8 (73%)	3 (27%)	0 (0%)
Education on discharge planning and transfer of care	ST	10 (91%)	1 (9%)	0 (0%)
Caregiver training	ST	11 (100%)	0 (0%)	0 (0%)
Education on self-management	ST	8 (73%)	2 (18%)	1 (9%)

Provision of information re: peer support to client and/or family (e.g., local stroke support group)	ST	7 (64%)	1 (9%)	3 (27%)
Goal-setting	ST	11 (100%)	0 (0%)	0 (0%)
Meta-cognitive strategy +/- cognitive training for executive function	ST	6 (55%)	5 (45%)	0 (0%)
Cognitive rehabilitation (remediation pen & paper tasks) for neglect	ST	3 (27%)	5 (45%)	3 (27%)
Cognitive rehabilitation (remediation leveraging on technology apps)	ST	4 (36%)	5 (45%)	2 (18%)
Cognitive rehabilitation (remediation functional tasks training)	ST	7 (64%)	3 (27%)	1 (9%)
Cognitive rehabilitation (compensatory strategies)	ST	6 (55%)	5 (45%)	0 (0%)
Behavioural approaches for dysphagia (e.g., swallowing exercises, environmental modifications, safe swallowing advice, and appropriate dietary modifications)	ST	10 (91%)	1 (9%)	0 (0%)
Recommendation of acupuncture for dysphagia	ST	0 (0%)	0 (0%)	11 (100%)
Surface Electromyography (sEMG) for dysphagia	ST	0 (0%)	3 (27%)	8 (73%)
Routine use of Neuromuscular Electrical Stimulation (NMES) for dysphagia	ST	0 (0%)	0 (0%)	11 (100%)
Intensive aphasia therapy (at least 45 minutes of direct language therapy for five days a week) in the first few months after stroke for dysphasia	ST	2 (18%)	1 (9%)	8 (73%)
Individually tailored interventions incorporating articulatory-kinematic and rate/rhythm approaches for speech apraxia (e.g., use of modelling and visual cueing, PROMPT therapy, self-administered computer programs that use multimodal sensory stimulation)	ST	5 (45%)	5 (45%)	1 (9%)
Non-speech oromotor exercises	ST	3 (27%)	7 (64%)	1 (9%)
Behavioural speech practice for dysarthria	ST	9 (82%)	2 (18%)	0 (0%)

Assistance/education of clients to maintain good oral and dental hygiene, particularly in those with swallowing difficulties	ST	11 (100%)	0 (0%)	0 (0%)
Education of staff and/or carers to maintain good oral and dental hygiene, particularly in those with swallowing difficulties	ST	10 (91%)	1 (9%)	0 (0%)

PT, Physiotherapy; OT, Occupational Therapy; ST, Speech and Language Therapy
*Allied health professionals include PT (n = 59), OT (n = 25) and ST (n = 11).

Table vi. Frequency of frailty treatments reported by allied health professionals*

Treatments	Profession	Often/ Always	Sometimes	Rarely/ Never
Referral to medical specialists (if required)	PT	23 (39%)	19 (32%)	17 (29%)
Referral to allied health professionals (e.g., dietician) (if required)	PT	18 (31%)	17 (29%)	24 (41%)
Education/Provision of information re: frailty care (can include physical activity counselling)	PT	33 (56%)	16 (27%)	10 (17%)
Caregiver training	PT	51 (86%)	5 (8%)	3 (5%)
Strengthening/resistance training (e.g., use of body weight, therabands, free weights, machines using weights and/or pneumatic resistance)	PT	58 (98%)	1 (2%)	0 (0%)
Power training (e.g., jumping or other form of plyometrics, ballistic training, complex training)	PT	12 (20%)	13 (22%)	34 (58%)
High Intensity Interval Training (HIIT)	PT	5 (8%)	8 (14%)	46 (78%)
Functional activities (e.g., sit-to-stands, transfers, squats, stairs)	PT	58 (98%)	1 (2%)	0 (0%)
Balance training (e.g., line walking, tandem foot standing, standing on one leg, heel-toe walking)	PT	55 (93%)	4 (7%)	0 (0%)
Aerobic training (e.g., overground/treadmill walking, cycling)	PT	55 (93%)	1 (2%)	3 (5%)
Flexibility training (e.g., stretching)	PT	46 (78%)	7 (12%)	6 (10%)
Dual-task training (e.g., walking and citing serial numbers)	PT	32 (54%)	15 (25%)	12 (20%)
Tai Chi	PT	3 (5%)	9 (15%)	47 (80%)
Dance	PT	4 (7%)	5 (8%)	50 (85%)
Functional cognitive training for mild cognitive impairment (MCI)	PT	7 (12%)	5 (8%)	47 (80%)
Home modifications (e.g.,EASE recommendations)	PT	12 (20%)	10 (17%)	37 (63%)
Prescription of equipment (e.g., walking aid, shower chair)	PT	39 (66%)	11 (19%)	9 (15%)
Referral to medical specialists (if required)	OT	5 (20%)	12 (48%)	8 (32%)

Referral to allied health professionals (e.g., dietician) (if required)	OT	3 (12%)	12 (48%)	10 (40%)
Education/Provision of information re: frailty care (can include physical activity counselling)	OT	11 (44%)	8 (32%)	6 (24%)
Caregiver training	OT	17 (68%)	7 (28%)	1 (4%)
Strengthening/resistance training (e.g., use of body weight, therabands, free weights, machines using weights and/or pneumatic resistance)	OT	20 (80%)	2 (8%)	3 (12%)
Power training (e.g., jumping or other form of plyometrics, ballistic training, complex training)	OT	2 (8%)	2 (8%)	21 (84%)
High Intensity Interval Training (HIIT)	OT	0 (0%)	2 (8%)	23 (92%)
Functional activities (e.g., sit-to-stands, transfers, squats, stairs)	OT	20 (80%)	3 (12%)	2 (8%)
Balance training (e.g., line walking, tandem foot standing, standing on one leg, heel-toe walking)	OT	8 (32%)	5 (20%)	12 (48%)
Aerobic training (e.g., overground/treadmill walking, cycling)	OT	7 (28%)	4 (16%)	14 (56%)
Flexibility training (e.g., stretching)	OT	9 (36%)	9 (36%)	7 (28%)
Dual-task training (e.g., walking and citing serial numbers)	OT	7 (28%)	7 (28%)	11 (44%)
Tai Chi	OT	0 (0%)	6 (24%)	19 (76%)
Dance	OT	0 (0%)	3 (12%)	22 (88%)
Functional cognitive training for mild cognitive impairment (MCI)	OT	10 (40%)	9 (36%)	6 (24%)
Home modifications (e.g.,EASE recommendations)	OT	15 (60%)	9 (36%)	1 (4%)
Prescription of equipment (e.g., walking aid, shower chair)	OT	15 (60%)	9 (36%)	1 (4%)
Referral to medical specialists (if required)	ST	7 (64%)	1 (9%)	3 (27%)
Referral to allied health professionals (e.g., dietician) (if required)	ST	8 (73%)	2 (18%)	1 (9%)
Education/Provision of information re: frailty care (can include physical activity counselling)	ST	5 (45%)	2 (18%)	4 (36%)

Caregiver training	ST	7 (64%)	1 (9%)	3 (27%)
Strengthening/resistance training (e.g., use of body weight, therabands, free weights, machines using weights and/or pneumatic resistance)	ST	0 (0%)	0 (0%)	11 (100%)
Power training (e.g., jumping or other form of plyometrics, ballistic training, complex training)	ST	0 (0%)	0 (0%)	11 (100%)
High Intensity Interval Training (HIIT)	ST	0 (0%)	0 (0%)	11 (100%)
Functional activities (e.g., sit-to-stands, transfers, squats, stairs)	ST	0 (0%)	1 (9%)	10 (91%)
Balance training (e.g., line walking, tandem foot standing, standing on one leg, heel-toe walking)	ST	(0%)	(0%)	11 (100%)
Aerobic training (e.g., overground/treadmill walking, cycling)	ST	(0%)	(0%)	11 (100%)
Flexibility training (e.g., stretching)	ST	0 (0%)	0 (0%)	11 (100%)
Dual-task training (e.g., walking and citing serial numbers)	ST	0 (0%)	1 (9%)	10 (91%)
Tai Chi	ST	0 (0%)	0 (0%)	11 (100%)
Dance	ST	0 (0%)	0 (0%)	11 (100%)
Functional cognitive training for mild cognitive impairment (MCI)	ST	1 (9%)	1 (9%)	9 (82%)
Home modifications (e.g.,EASE recommendations)	ST	0 (0%)	0 (0%)	11 (100%)
Prescription of equipment (e.g., walking aid, shower chair)	ST	0 (0%)	0 (0%)	11 (100%)
Behavioural approaches for dysphagia (e.g., swallowing exercises, environmental modifications, safe swallowing advice, and appropriate dietary modifications)	ST	10 (91%)	1 (9%)	0 (0%)
Assistance/education of clients to maintain good oral and dental hygiene, particularly in those with swallowing difficulties	ST	10 (91%)	1 (9%)	0 (0%)
Education of staff and/or carers to maintain good oral and dental hygiene, particularly in those with swallowing difficulties	ST	9 (82%)	2 (18%)	0 (0%)

PT, Physiotherapy; OT, Occupational Therapy; ST, Speech and Language Therapy
*Allied health professionals include PT (n = 59), OT (n = 25) and ST (n = 11).

Table vii. Frequency of hip fracture treatments reported by allied health professionals*

**Treatments**	**Profession**	**Often/ Always**	**Sometimes**	**Rarely/ Never**
Referral to medical specialists (if required)	PT	23 (39%)	14 (24%)	22 (37%)
Referral to allied health professionals (e.g., dietician) (if required)	PT	22 (37%)	10 (17%)	27 (46%)
Education/Provision of information re: hip fracture (can include falls prevention)	PT	53 (90%)	3 (5%)	(5%)
Caregiver training	PT	52 (88%)	4 (7%)	3 (5%)
Group circuit class therapy	PT	16 (%)	12 (20%)	31 (53%)
Strengthening/resistance training (e.g., use of body weight, therabands, free weights, machines using weights and/or pneumatic resistance)	PT	57 (97%)	1 (2%)	1 (2%)
Power training (e.g., jumping or other form of plyometrics, ballistic training, complex training)	PT	12 (20%)	9 (15%)	38 (64%)
High Intensity Interval Training (HIIT)	PT	8 (14%)	11 (19%)	40 (68%)
Functional activities (e.g., sit-to-stands, transfers, squats, stairs)	PT	57 (97%)	0 (0%)	2 (3%)
Balance training (e.g., line walking, tandem foot standing, standing on one leg, heel-toe walking)	PT	55 (93%)	2 (3%)	2 (3%)
Aerobic training (e.g., overground/treadmill walking, cycling)	PT	53 (90%)	3 (5%)	3 (5%)
Flexibility training (e.g., stretching)	PT	43 (73%)	11 (19%)	5 (8%)
Dual-task training (e.g., walking and citing serial numbers)	PT	29 (49%)	10 (17%)	20 (34%)
Tai Chi	PT	4 (7%)	5 (8%)	50 (85%)
Dance	PT	4 (7%)	4 (7%)	51 (86%)
Home modifications (e.g., rails)	PT	21 (36%)	11 (19%)	27 (46%)
Prescription of equipment (e.g., walking aid, shower chair)	PT	35 (59%)	12 (20%)	12 (20%)
Prescription of hip protector	PT	6 (10%)	5 (8%)	48 (81%)
Prescription of aids for communication (e.g., eyeglasses, hearing aids)	PT	5 (8%)	7 (12%)	47 (80%)
Referral to medical specialists (if required)	OT	4 (16%)	10 (40%)	11 (44%)

Referral to allied health professionals (e.g., dietician) (if required)	OT	2 (8%)	13 (52%)	10 (40%)
Education/Provision of information re: hip fracture (can include falls prevention)	OT	19 (76%)	2 (8%)	4 (16%)
Caregiver training	OT	18 (72%)	4 (16%)	3 (12%)
Group circuit class therapy	OT	0 (0%)	3 (12%)	22 (88%)
Strengthening/resistance training (e.g., use of body weight, therabands, free weights, machines using weights and/or pneumatic resistance)	OT	16 (64%)	2 (8%)	7 (28%)
Power training (e.g., jumping or other form of plyometrics, ballistic training, complex training)	OT	1 (4%)	3 (12%)	21 (84%)
High Intensity Interval Training (HIIT)	OT	0 (0%)	2 (8%)	23 (92%)
Functional activities (e.g., sit-to-stands, transfers, squats, stairs)	OT	16 (64%)	6 (24%)	3 (12%)
Balance training (e.g., line walking, tandem foot standing, standing on one leg, heel-toe walking)	OT	9 (36%)	4 (16%)	12 (48%)
Aerobic training (e.g., overground/treadmill walking, cycling)	OT	10 (40%)	4 (16%)	11 (44%)
Flexibility training (e.g., stretching)	OT	8 (32%)	6 (24%)	11 (44%)
Dual-task training (e.g., walking and citing serial numbers)	OT	6 (24%)	9 (36%)	10 (40%)
Tai Chi	OT	0 (0%)	5 (20%)	20 (80%)
Dance	OT	0 (0%)	1 (4%)	24 (96%)
Home modifications (e.g., rails)	OT	15 (60%)	6 (24%)	4 (16%)
Prescription of equipment (e.g., walking aid, shower chair)	OT	15 (60%)	6 (24%)	4 (16%)
Prescription of hip protector	OT	1 (4%)	1 (4%)	23 (92%)
Prescription of aids for communication (e.g., eyeglasses, hearing aids)	OT	1 (4%)	8 (32%)	16 (64%)

PT, Physiotherapy; OT, Occupational Therapy; ST, Speech and Language Therapy
*Allied health professionals include PT (n = 59), OT (n = 25) and ST (n = 11).

Table viii. Caseload across day rehabilitation centres.

A) Target number of clients that allied health professionals are expected to see per day as reported by rehabilitation leaders (n = 29), and

**Allied Health Professional**	**Mean target number of clients seen per day**	**Range**
Physiotherapist	11	0 to 25
Occupational therapist	10	0 to 18
Speech and Language therapist	4	0 to 12

B) Estimated number of clients with stroke deconditioning or hip fracture that allied health professionals are expected to see per month by rehabilitation leaders (n = 29)

**Conditions**	**Mean target number seen per month**	**Range**
Clients with Stroke	25	3 to 80
Clients with Deconditioning	19	0 to 100
Clients with Hip fracture	8	0 to 40

Figure i. Manpower allocation across day rehabilitation centres. A) Proportion of sites reporting availability of manpower at day rehabilitation centres, and B) Proportion of sites reporting manpower capacity at day rehabilitation centres, as reported by rehabilitation leaders (n = 29).

A)

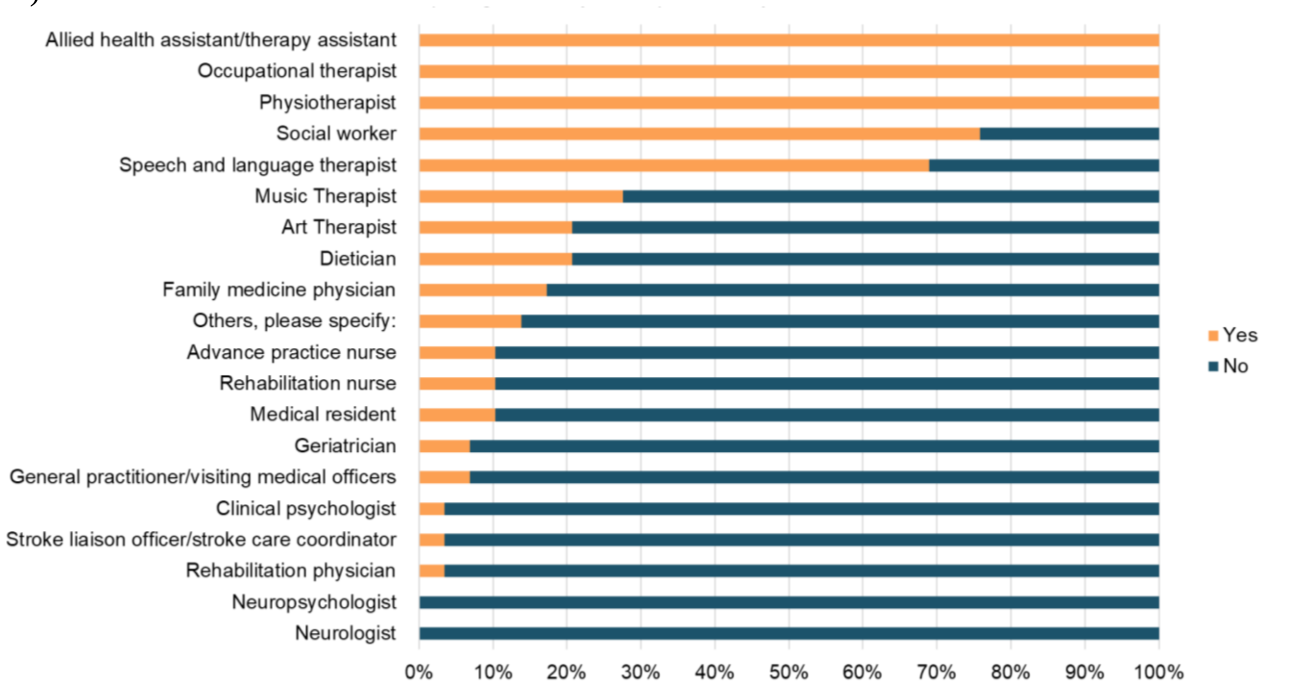

B)

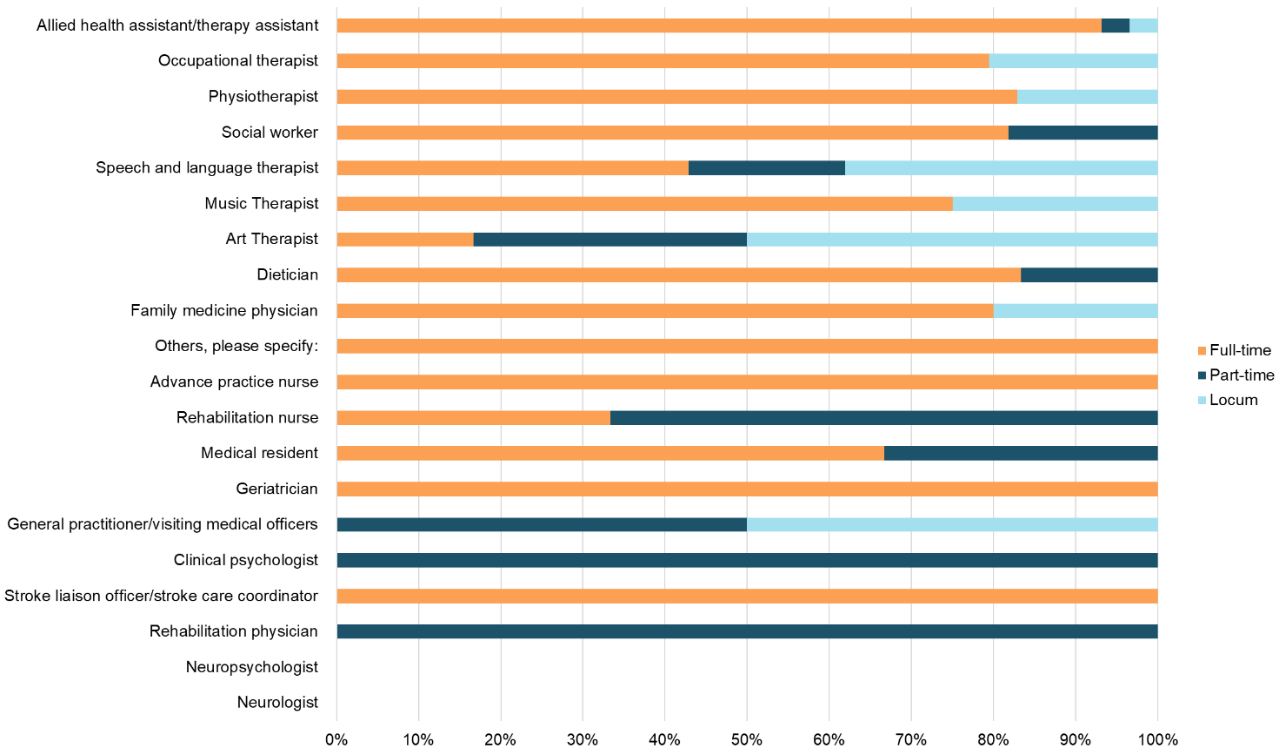

Figure ii. Practices surrounding active and maintenance rehabilitation. A) Proportion of sites reporting use of factor to guide decision making between active and maintenance rehabilitation; B) Proportion of sites reporting frequency of goals/care plan for active and maintenance rehabilitation; and C) Proportion of sites reporting evaluation method of goals/care plan for active and maintenance rehabilitation.

A)

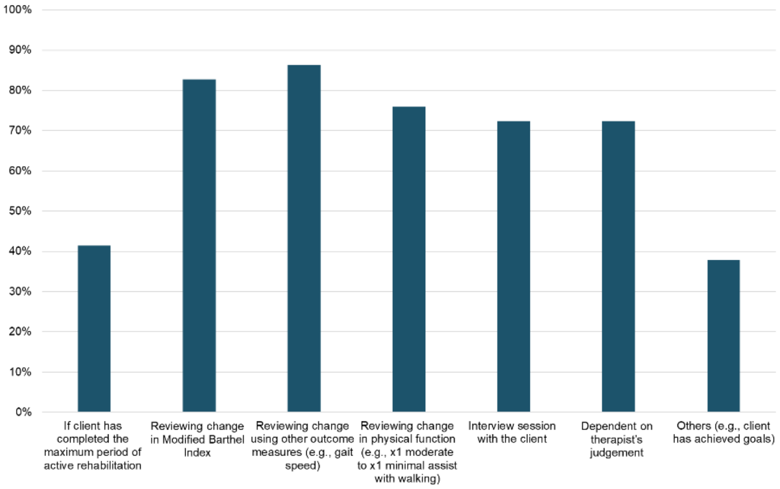

B)

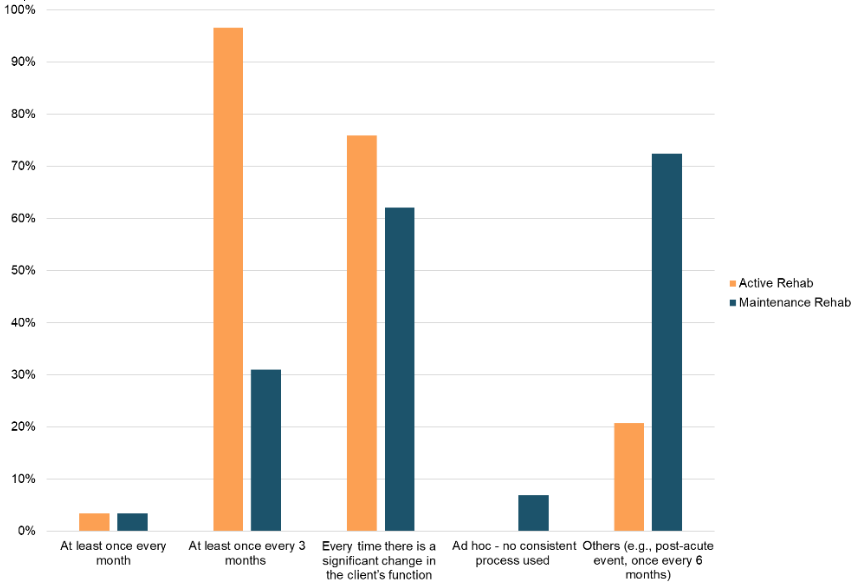

C)

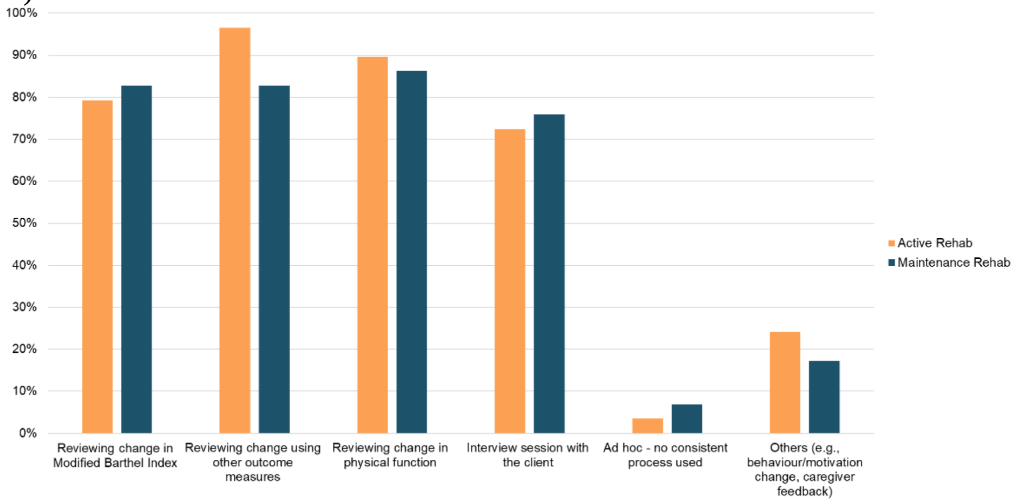

Figure iii. Practice of skills-sharing reported by A) allied health professionals (n = 95) and B) rehabilitation leaders (n = 29)

A)

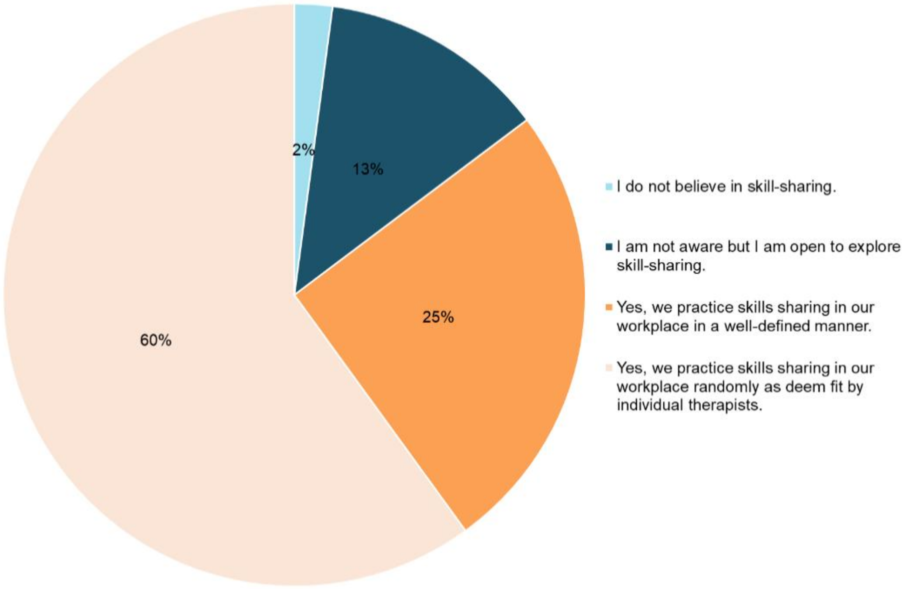

B)

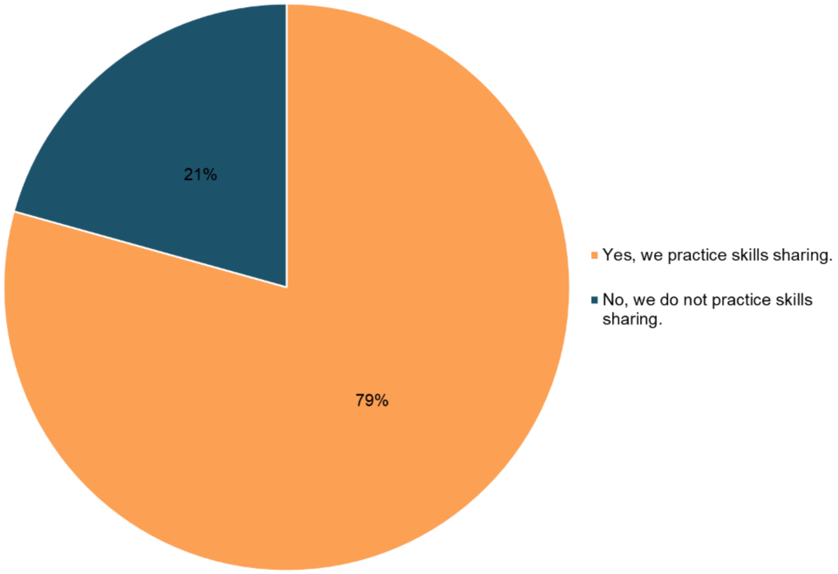

Figure iv. Clinical areas where skills sharing is perceived to be possible by A) allied health professionals (n = 95), B) physiotherapists (n = 59), C) occupational therapists (n = 25) and D) speech and language therapists (n = 11)

A)

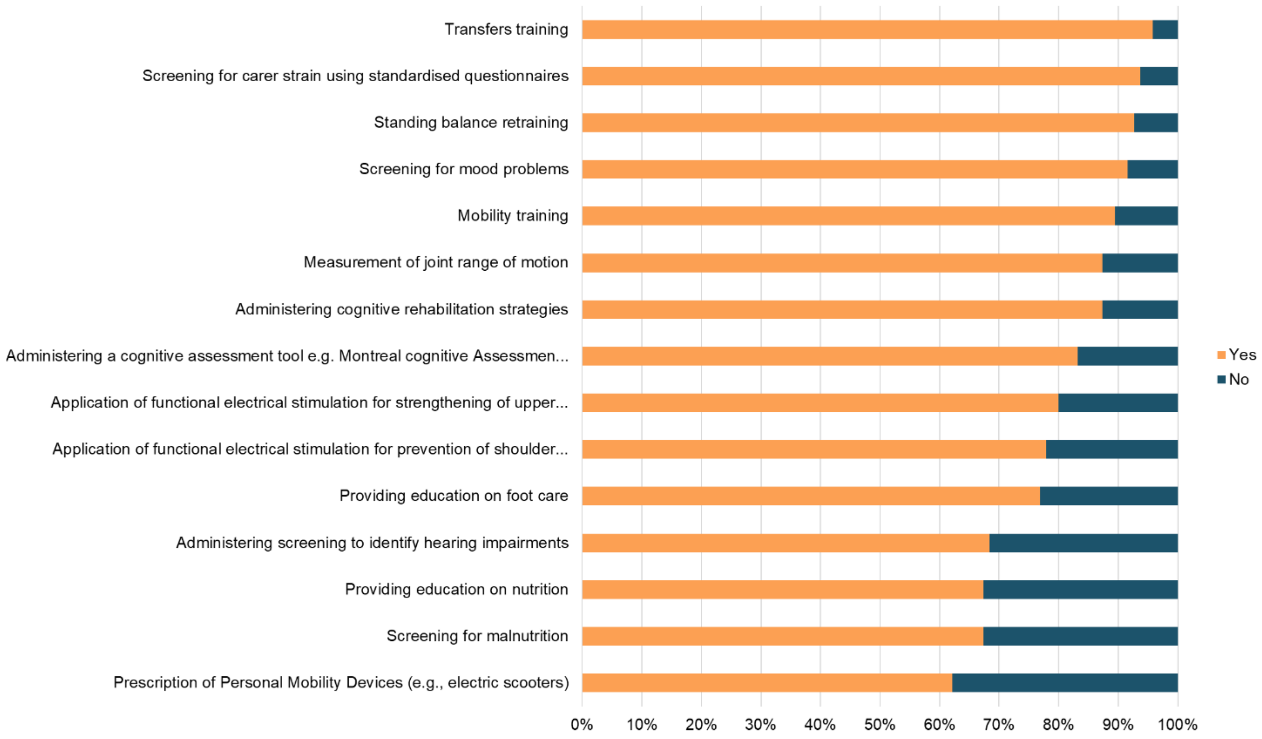

B)

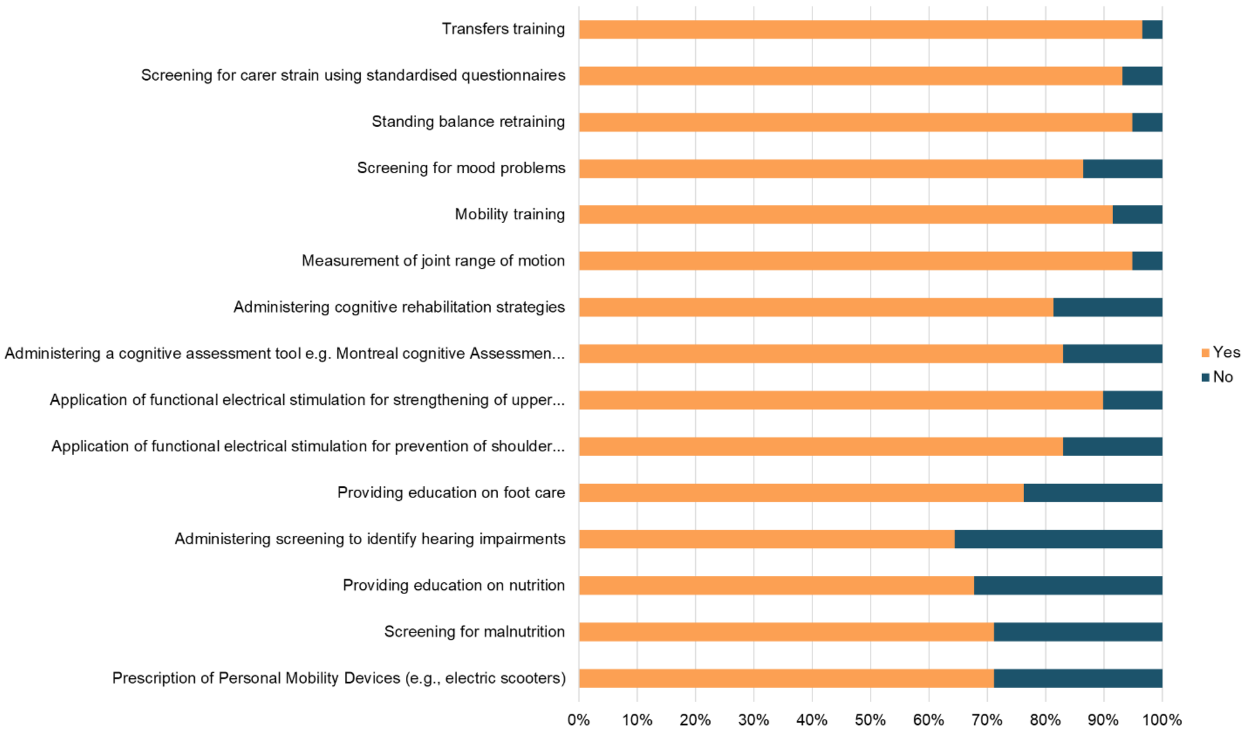

C)

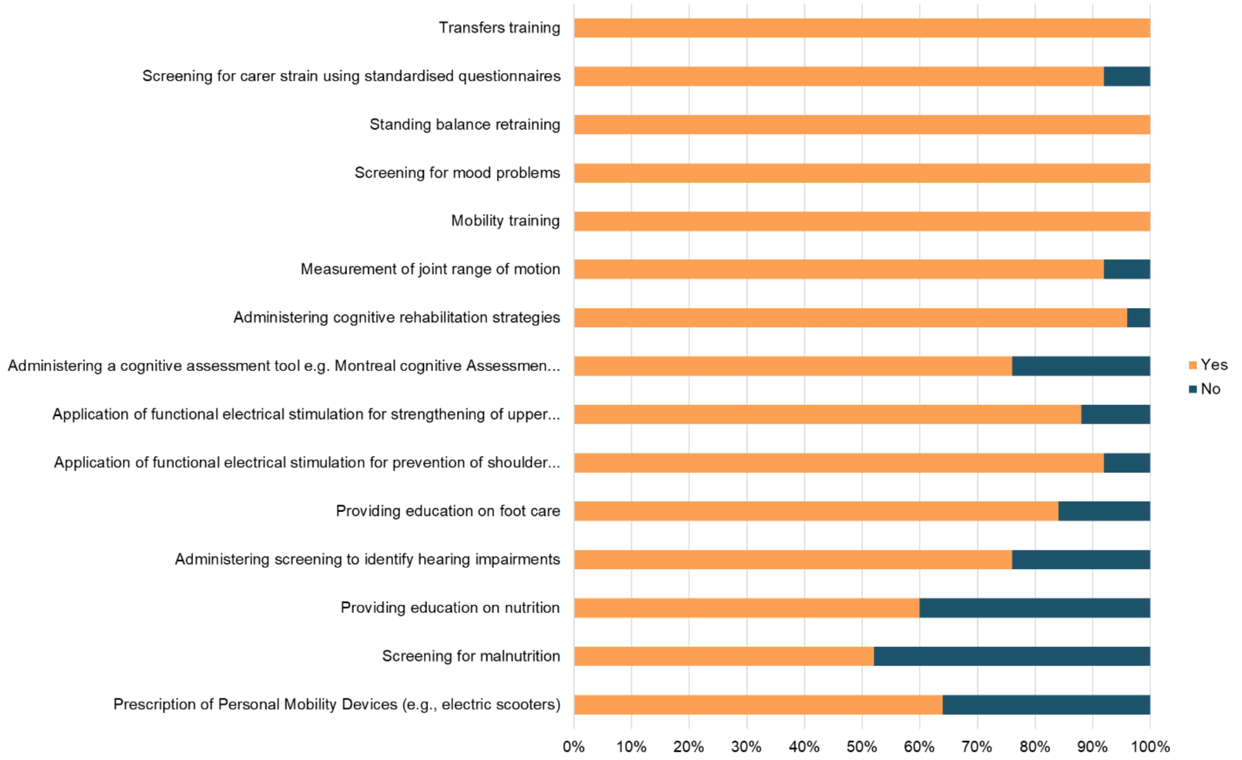
Transfers training
Screening for carer strain using standardised questionnaires
Standing balance retraining
Screening for mood problems
Mobility training
Measurement of joint range of motion
Administering cognitive rehabilitation strategies
Administering a cognitive assessment tool e.g. Montreal cognitive Assessmen...
Application of functional electrical stimulation for strengthening of upper...
Application of functional electrical stimulation for prevention of shoulder...
Providing education on foot care
Administering screening to identify hearing impairments
Providing education on nutrition
Screening for malnutrition
Prescription of Personal Mobility Devices (e.g., electric scooters)
0%
10%
20%
30%
40%
50%
60%
70%
80%
90%
100%
Yes
No

D)

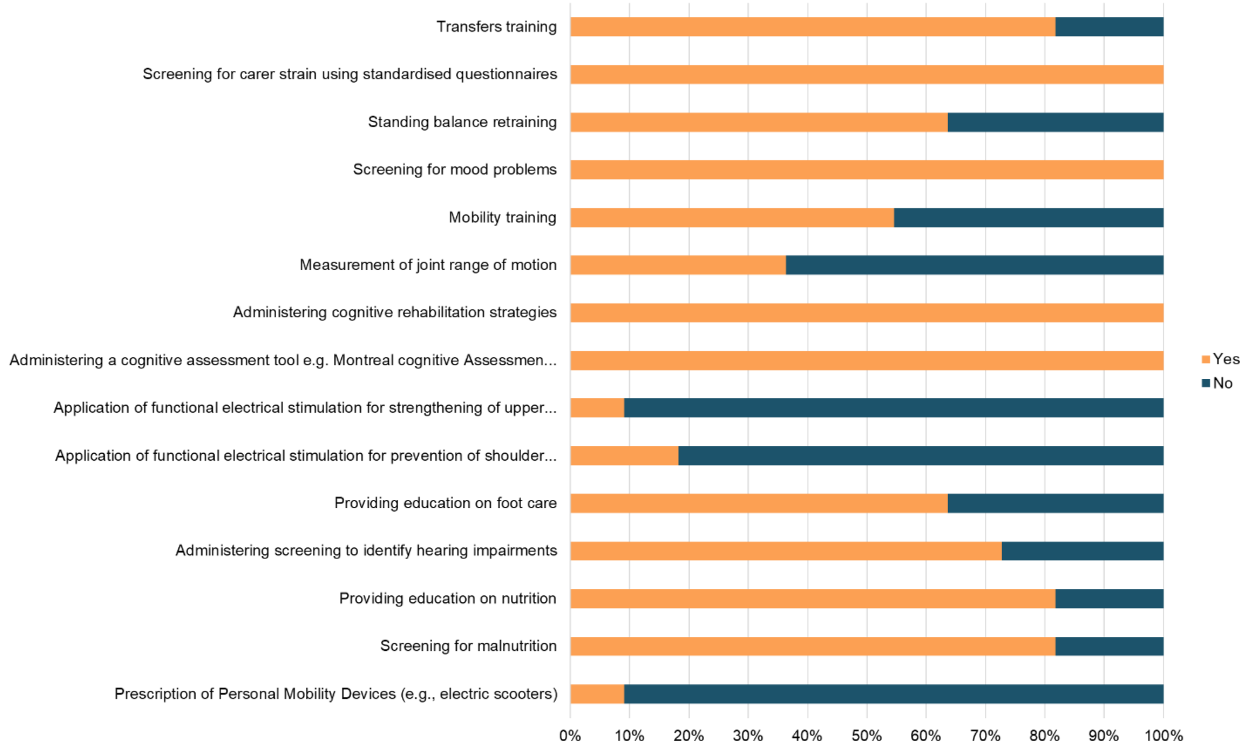
Transfers training
Screening for carer strain using standardised questionnaires
Standing balance retraining
Screening for mood problems
Mobility training
Measurement of joint range of motion
Administering cognitive rehabilitation strategies
Administering a cognitive assessment tool e.g. Montreal cognitive Assessmen...
Application of functional electrical stimulation for strengthening of upper...
Application of functional electrical stimulation for prevention of shoulder...
Providing education on foot care
Administering screening to identify hearing impairments
Providing education on nutrition
Screening for malnutrition
Prescription of Personal Mobility Devices (e.g., electric scooters)
0%
10%
20%
30%
40%
50%
60%
70%
80%
90%
100%
Yes
No

Figure v. Availability of processes at site for skills-sharing reported by rehabilitation leaders (n = 29)

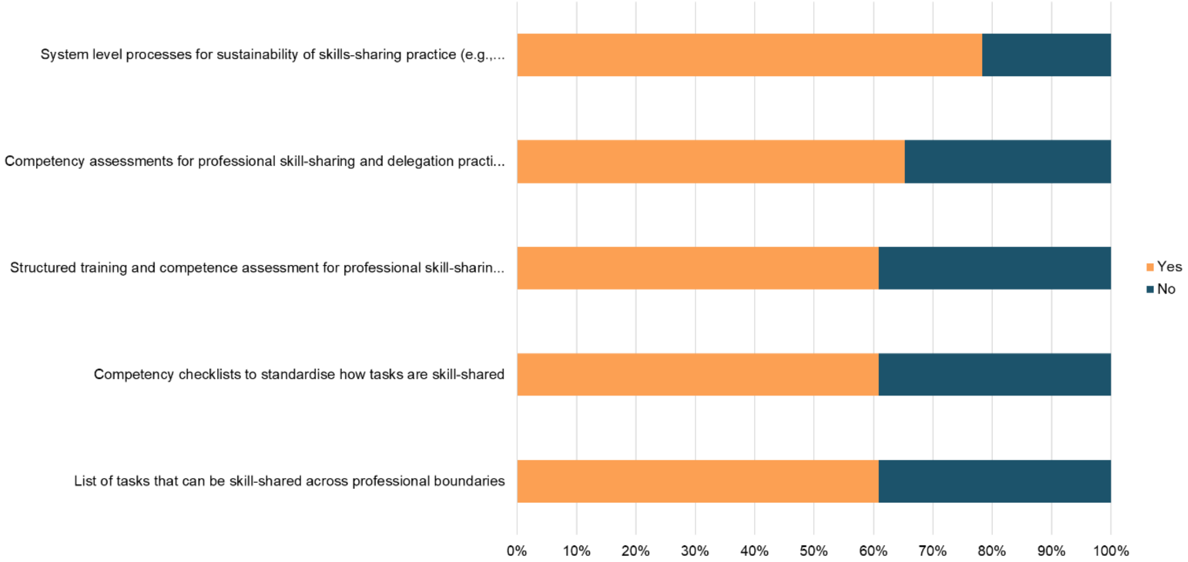

Supplement: Supplementary file 8 — Supporting Information 8. Additional data from surveys and case note reviews. [file HEX-28-e70330-s005.pdf]
